# Copper-catalyzed cascade annulation of unsaturated α-bromocarbonyls with enynals: a facile access to ketones from aldehydes†

**DOI:** 10.1039/c5sc04980f

**Published:** 2016-03-04

**Authors:** Chao Che, Qianwen Huang, Hanliang Zheng, Gangguo Zhu

**Affiliations:** a Department of Chemistry, Zhejiang Normal University 688 Yingbin Road Jinhua 321004 China gangguo@zjnu.cn

## Abstract

A Cu-catalyzed cascade annulation of enynals with alkenyl or alkynyl α-bromocarbonyls for the synthesis of various cyclohexenone-fused polycyclic compounds is described. Up to six new C–C bonds and four new carbocycles can be established in a single reaction, highlighting the high efficiency and step-economics of this protocol. This reaction offers a novel and straightforward entry to the synthesis of ketones featuring the addition of carbon radicals to aldehydes.

## Abstract 2

A Cu-catalyzed cascade annulation of enynals with alkenyl or alkynyl α-bromocarbonyls for the synthesis of various cyclohexenone-fused polycyclic compounds is described.

## Graphical abstract

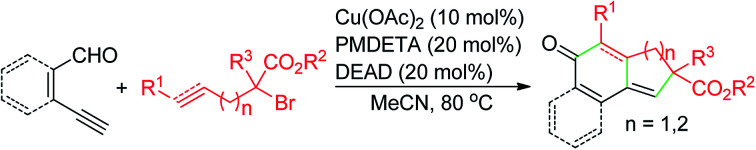

Ketones are ubiquitous chemical entities in bioactive molecules, drugs and materials (Scheme 1).^1^ Typically, they are prepared by the addition of organometallic compounds to aldehydes, followed by oxidation, which requires the utilization of stoichiometric organometallic reagents and oxidants. Alternatively, the aldehydic C–H bond functionalization^2–7^ has become a powerful strategy for assembling ketones because of its outstanding advantages in atom and step efficiency. Among these, the radical reactions have attracted more and more attention.^4–7^ For example, a *N*-hydroxyphthalimide catalyzed radical hydroacylation of simple alkenes with aldehydes has been achieved by the Ishii group.^4^ More recently, Lei and co-workers reported an elegant synthesis of α,β-unsaturated ketones *via* the Cu-catalyzed oxidative coupling of terminal alkenes with aldehydes.^5^ It should be noted that most of these reactions depend on the generation and transformation of acyl radical A (type I) (Scheme 2a).^7^ However, the type II version, with aldehydes as acceptors for the addition of carbon radicals,^8^ has never been realized for the access of ketones (type II) (Scheme 2b). This may be ascribed to the higher dissociation energy of C–H bonds as compared to that of C–C bonds, and consequently, the alkoxyl radical B strongly prefers to proceed *via* the C–C β-scission, instead of the C–H β-scission.^9^ As such, the intermediate B is in favor of transforming back to aldehydes.

**Scheme 1:**
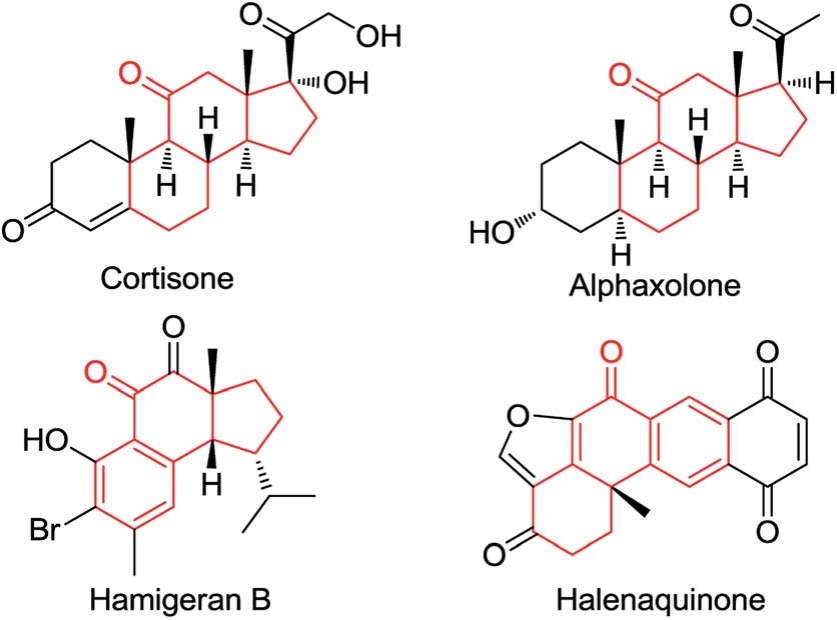
Examples of bioactive ketones.

**Scheme 2:**
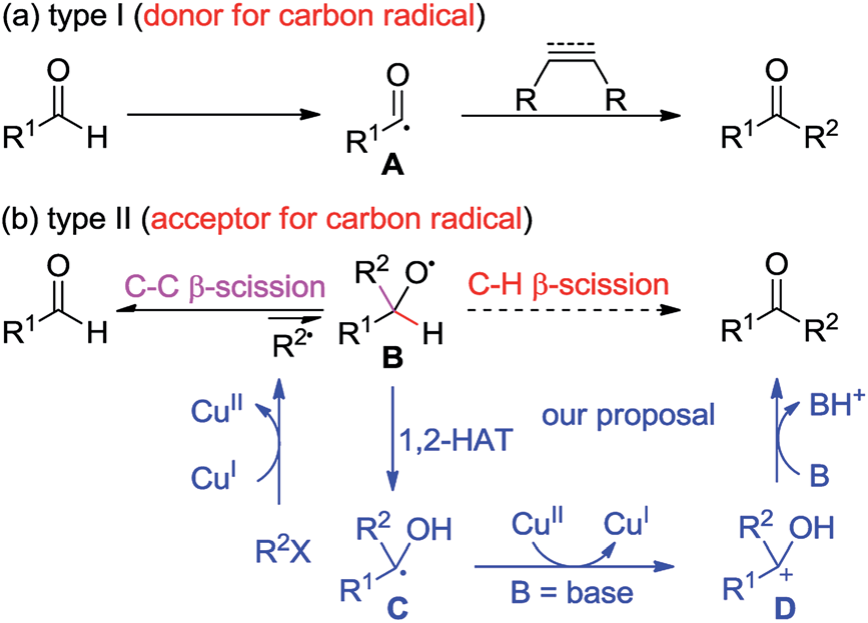
Radical approaches to ketones from aldehydes and our proposal.

Given the high efficiency of this transformation (type II), we decided to explore the feasibility. While pursuing our recent work on the Cu-catalyzed atom-transfer radical addition (ATRA) of alkynes,^10–12^ we envisaged that the direct conversion of aldehydes into ketones might be accomplished *via* a Cu-catalyzed redox-neutral pathway, which consists of the following steps: (1) a single-electron transfer (SET) between the Cu(i) catalyst and organohalides (R^2^X) produces a radical R^2^˙, together with the formation of Cu(ii), (2) the alkoxyl radical B, resulting from the addition of R^2^˙ to R^1^CHO, undergoes a formal 1,2-H atom shift^13^ to afford the carbon-centered radical C, (3) another SET between C and Cu(ii) species delivers a cationic intermediate D accompanied by the regeneration of the Cu(i) catalyst, and (4) deprotonation of D gives ketones as the final products. Herein, we describe a Cu-catalyzed cascade annulation of alkenyl or alkynyl α-bromocarbonyls with enynals, providing a variety of polycyclic ketones in moderate to excellent yields under mild reaction conditions. In this reaction, up to six new C–C bonds and four new rings can be assembled from the readily attained starting materials, highlighting the high efficiency and step-economics of this method.

To test this hypothesis, the reaction between 2-ethynylbenzaldehyde (1a) and diethyl α-allyl-α-bromomalonate (2a) was conducted in MeCN. Using 10 mol% of CuBr as the catalyst, 20 mol% of pentamethyldiethylenetriamine (L1) as the ligand, and 1 equivalent of K_2_CO_3_ as the base, tricyclic ketone 3aa was isolated in 42% yield, after being heated at 80 °C for 10 h (Table 1, entry 1). Encouraged by this result, we further screened the reaction parameters. To our satisfaction, using diethylazodicarboxylate (DEAD) as the reducing reagent for *in situ* generation of the Cu(i) catalyst, the reaction afforded 3aa in 86% yield (entry 4). Employment of other ligands such as L2–L4 and L5 resulted in decreased yields (entries 6–9). Replacing DEAD with either azodiisobutyrodinitrile (AIBN) or 2,2′-azobis(2,4-dimethylvaleronitrile) (V65) led to inferior results (entries 10 and 11). As for the solvent, MeCN demonstrated better performance than other solvents such as THF, toluene and DMF (entries 14–16).

**Table 1:** Optimization of the reaction conditions^a^

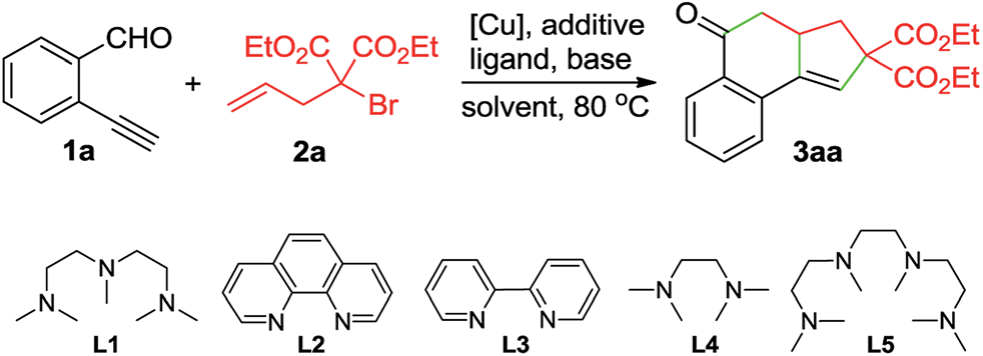						
Entry	[Cu]	Ligand	Additive	Base	Solvent	Yield (%)
1	CuBr	L1	—	K_2_CO_3_	MeCN	42
2	CuBr_2_	L1	DEAD	K_2_CO_3_	MeCN	36
3	Cu(acac)_2_	L1	DEAD	K_2_CO_3_	MeCN	83
4	Cu(OAc)_2_	L1	DEAD	K_2_CO_3_	MeCN	86
5	Cu(OAc)_2_	L1	—	K_2_CO_3_	MeCN	61
6	Cu(OAc)_2_	L2	DEAD	K_2_CO_3_	MeCN	52
7	Cu(OAc)_2_	L3	DEAD	K_2_CO_3_	MeCN	34
8	Cu(OAc)_2_	L4	DEAD	K_2_CO_3_	MeCN	75
9	Cu(OAc)_2_	L5	DEAD	K_2_CO_3_	MeCN	80
10	Cu(OAc)_2_	L1	AIBN	K_2_CO_3_	MeCN	62
11	Cu(OAc)_2_	L1	V65	K_2_CO_3_	MeCN	65
12	Cu(OAc)_2_	L1	DEAD	Cs_2_CO_3_	MeCN	47
13	Cu(OAc)_2_	L1	DEAD	DBU	MeCN	21
14	Cu(OAc)_2_	L1	DEAD	K_2_CO_3_	THF	Trace
15	Cu(OAc)_2_	L1	DEAD	K_2_CO_3_	PhMe	Trace
16	Cu(OAc)_2_	L1	DEAD	K_2_CO_3_	DMF	14

a *Reaction conditions*: 1a (0.25 mmol), 2a (0.30 mmol), [Cu] (10 mol%), ligand (20 mol%), additive (20 mol%), base (0.25 mmol), solvent (3 mL), under N_2_ 80 °C, 10 h. Yields of the isolated products are given.

With the optimized reaction conditions in hand, we investigated the scope of this Cu-catalyzed domino annulation by varying enynals 1 and α-bromocarbonyls 2. As shown in Table 2, the standard conditions were well compatible with a variety of enynals, including 2-ethynylbenzaldehydes and pent-2-en-4-ynal derivatives. Substrates with different substituents on the aryl ring of 1 were successfully converted into polycyclic ketones in good to excellent yields, regardless of the electronic effects of the substituents (3ba–3ia). Halogen atoms such as F and Cl were well tolerated under the reaction conditions (3da–3fa), giving ample opportunities for further elaboration by the transition-metal-catalyzed coupling reactions. Intriguingly, the reaction of 1m–1o with 2a occurred uneventfully to provide tetracyclic ketones 3ma–3oa in high yields. Aldehyde 1p with the 2-thienyl group was transformed into the corresponding ketone 3pa in 68% yield. The process was extended to substrate 1q, bearing an amide group, providing 3qa in a good yield. Moreover, 2-ethynylcyclohex-1-enecarbaldehyde (1r) was also a competent substrate, and 3ra was synthesized without erosion of the reaction yield.

**Table 2:** Scope of enynals^a^

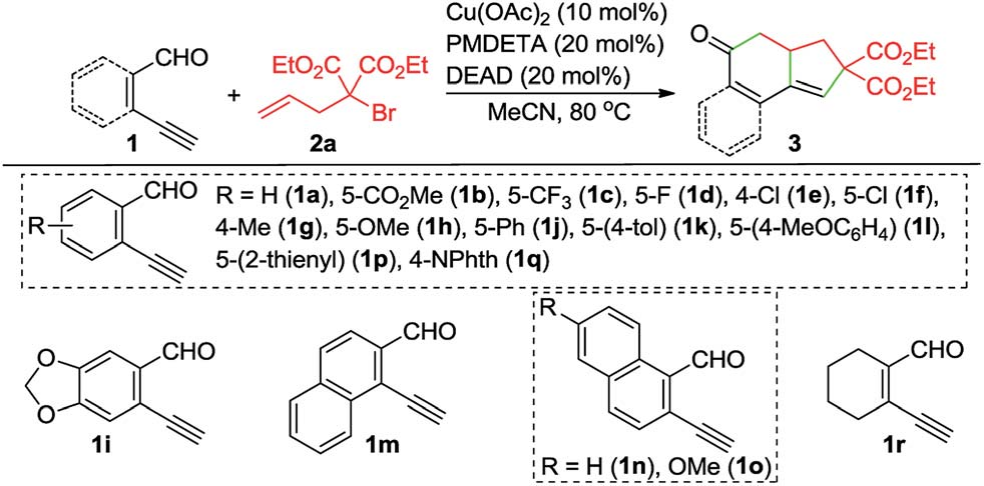
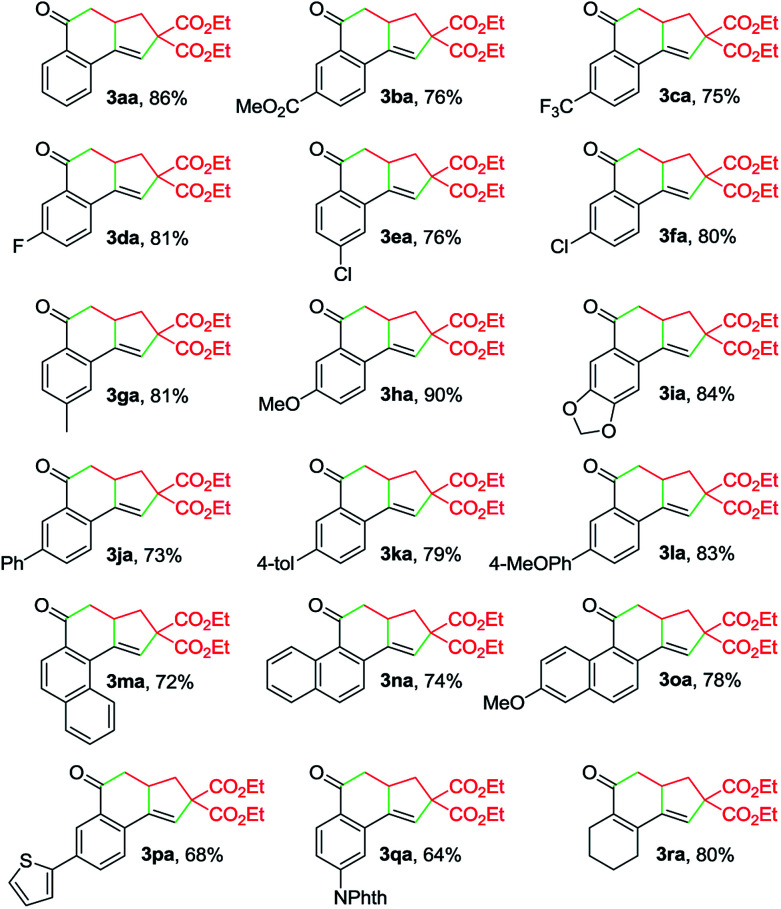

a *Reaction conditions*: 1 (0.25 mmol), 2a (0.30 mmol), Cu(OAc)_2_ (10 mol%), L1 (20 mol%), DEAD (20 mol%), K_2_CO_3_ (0.25 mmol), MeCN (3 mL), under N_2_, 80 °C, 10 h. Yields of the isolated products are given. NPhth = phthalimidyl.

By varying α-bromo γ,δ-unsaturated carbonyl compounds 2 with 1a as the coupling partner, further examples of tricyclic ketones (3ab–3ai) were synthesized (Table 3). The product 3ab, containing a *gem*-dimethyl subunit, was isolated in an excellent yield. Substitution of the terminal C–C double bond of 2 with a methyl group resulted in the production of 3ac in 80% yield and good diastereoselectivity (dr = 88 : 12). In contrast, the Ph-substituted analogue 2d was not suitable for this Cu-catalyzed domino process (3ad). In the case of β-branched substrate 2f, the reaction produced 3af in a moderate yield. The reaction covered other activated organobromides, as exemplified by the construction of 3ag and 3ah. Compound 2i, a weakly activated substrate, was effective for the transformation, while no detectable product was observed when secondary bromide 2j was used as the coupling partner (3ai and 3aj). This reaction was well amenable to propargyl α-bromocarbonyls. For example, the coupling of 1a with 2k also took place, affording 3ak in 74% yield. Substitution of the terminal alkynyl carbon by primary alkyl groups led to the facile generation of tricyclic ketones (3ak–3am), whereas the cyclopropane-substituted counterpart 2n delivered the corresponding product in a lower yield (3an), potentially due to the increased steric hindrance. Meanwhile, an α-bromo δ,ε-unsaturated carbonyl such as 2p performed well in this Cu-catalyzed cascade annulation reaction, giving a direct and convenient access to the 6-6-6-tricyclic ketone 3ap. The structure of polycyclic ketones 3fa and 3ap was determined by the X-ray diffraction analysis.^14^

**Table 3:** Scope of α-bromocarbonyl compounds^a^

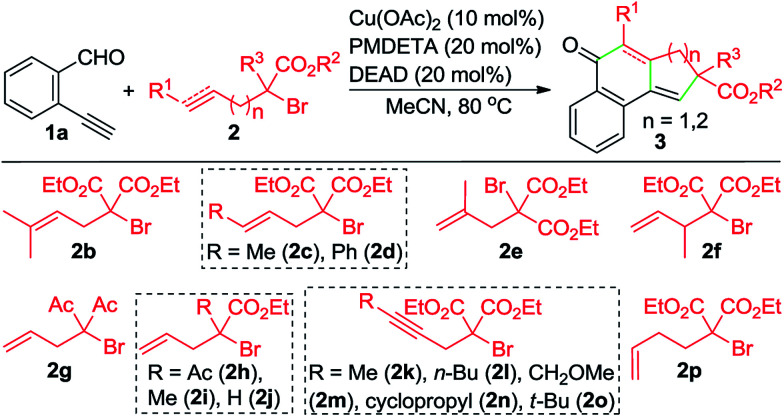
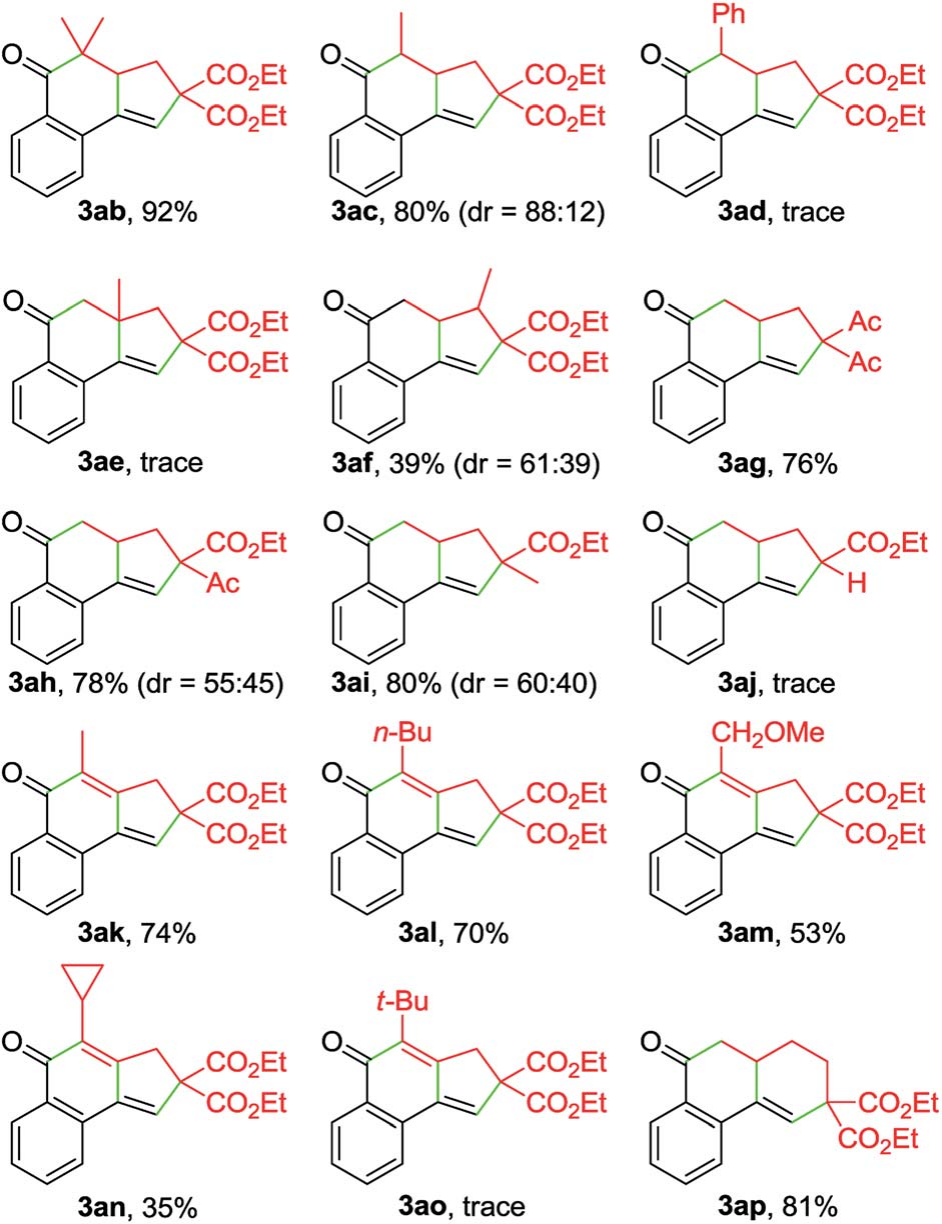

a *Reaction conditions*: 1a (0.25 mmol), 2 (0.30 mmol), Cu(OAc)_2_ (10 mol%), L1 (20 mol%), DEAD (20 mol%), K_2_CO_3_ (0.25 mmol), MeCN (3 mL), under N_2_, 80 °C, 10 h. Yields of the isolated products are given.

Remarkably, the one-pot construction of pentacyclic diketones 3sa and 3sp was achieved by reacting enynal 1s with 2a and 2p, respectively (Scheme 3). Although the yield appears to be moderate, considering the formation of six new C–C bonds and four new rings in a single reaction, it still represents a highly attractive method for the synthesis of polycyclic ketones from readily accessible starting materials.

**Scheme 3:**
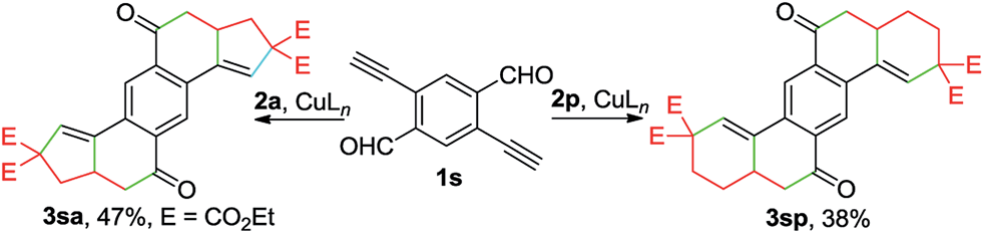
Cu-catalyzed double cascade annulation.

To gain insights into the reaction mechanism, a series of experiments were performed. First, the reaction between 1a and 2a was inhibited by adding 2 equivalents of 2,2,6,6-tetramethylpiperidinooxy (TEMPO), and instead, 4a was formed in 51% yield (eqn (1)). In the presence of butylated hydroxytoluene (BHT), no detectable 3aa was observed, and 4b was obtained in 35% yield (eqn (2)). Likewise, the addition of 1,1-diphenylethylene hindered the reaction between 1a and 2b and provided the Cu-catalyzed atom-transfer radical cyclization^12*a*^ product 4c in 68% yield (eqn (3)). These results indicated that the Cu-catalyzed cascade annulation reaction might proceed *via* a radical mechanism. Furthermore, when compound 5 was employed as the starting material, alcohol 6a was obtained in 62% yield (eqn (4)), implying that the aldehydic hydrogen atom of 1 is essential for the ketone synthesis. Alcohol 6b, generated by the reduction of 3aa with NaBH_4_, was subjected to the optimized reaction conditions, and as a result, no formation of 3aa was observed (eqn (5)). It indicated that the formation of alcohol intermediate 6b followed by oxidation with Cu(ii) reagents is less likely in this case.1
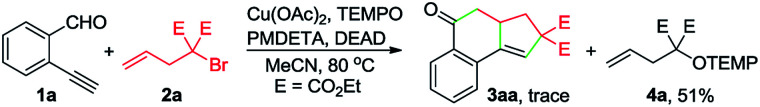
2
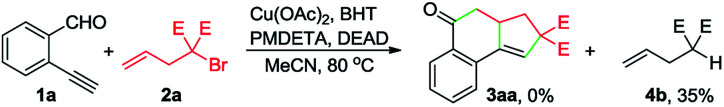
3
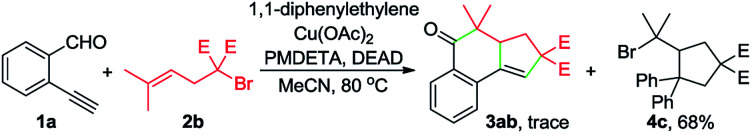
4
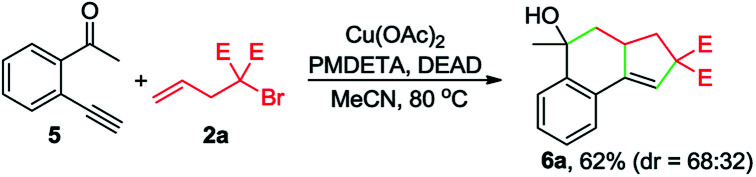
5
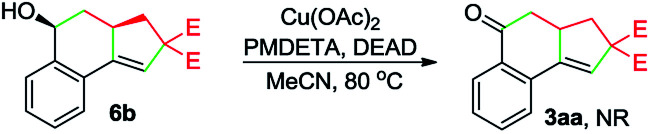


Whereas the full mechanistic features of this Cu-catalyzed domino annulation are still under investigation, a working mechanism is proposed in Scheme 4, using 1a and 2a as representative starting materials. Initially, a radical I is formed by a SET process from 2a and Cu(i) catalyst, which is generated *in situ* by the reduction of Cu(OAc)_2_ with DEAD. The isolation of adduct 4a confirmed the formation of radical I. The radical I adds to the C–C triple bond of 1a to deliver an alkenyl radical II, which is converted to the alkyl radical species III*via* a 5-*exo*-trig cyclization. Then, an intramolecular addition of carbon radical to the aldehyde group generates the alkoxy radical IV, followed by a formal 1,2-H shift^13^ to give the benzyl radical V. Subsequently, a second SET between V and Cu(ii) produces the cationic intermediate VI with the regeneration of Cu(i) catalyst. Finally, VI is deprotonated to afford the tricyclic ketone 3aa with the aid of K_2_CO_3_.

**Scheme 4:**
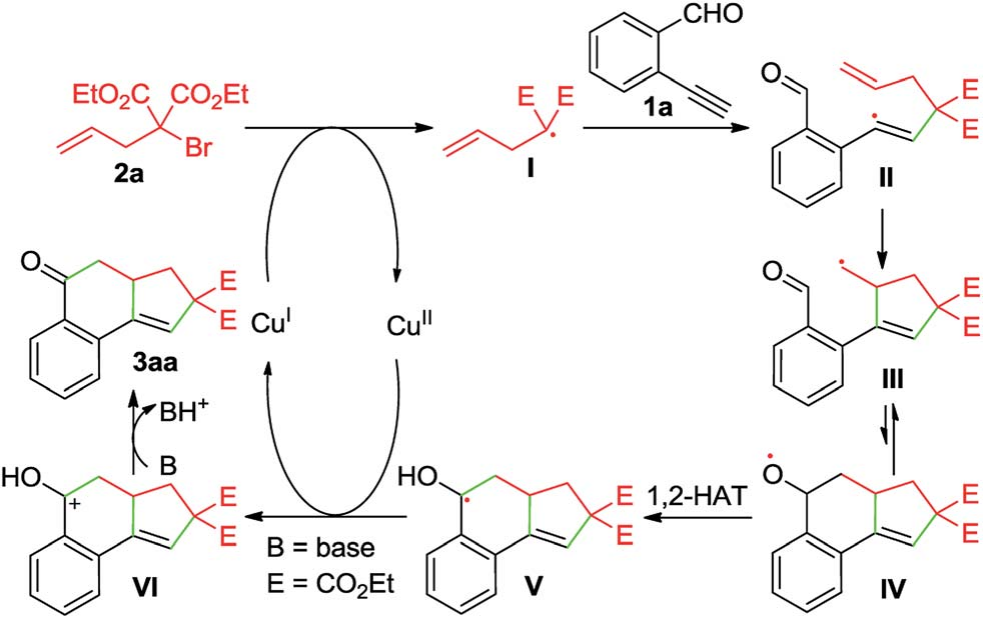
A possible mechanism.

The synthetic utility of this reaction was also explored (Scheme 5). Treatment of 3aa with LiCl and H_2_O in DMSO at reflux^15^ resulted in the production of 80% yield of 3aj, a product that was not able to be synthesized *via* the Cu-catalyzed cascade annulation (Table 3, 3aj). Obviously, the decarbalkoxylation procedure offered a good complementary method to the domino annulation. Epoxidation of 3fa with *meta*-chloroperbenzoic acid (*m*-CPBA) gave rise to a single diastereoisomer, 7b, in 76% yield.^14^ Furthermore, the one-pot synthesis of α-bromo diketone 7c could be accomplished through the exposure of 3aa to a combination of *N*-bromosuccinimide (NBS) and NH_4_OAc in Et_2_O.^16^ By treating 3ak with NaBH_4_ in a 1 : 1 mixture of MeOH and THF, the 1,6-addition product 7d was obtained in 92% yield, which constitutes a new efficient access to polysubstituted 1-naphthols.^17^

**Scheme 5:**
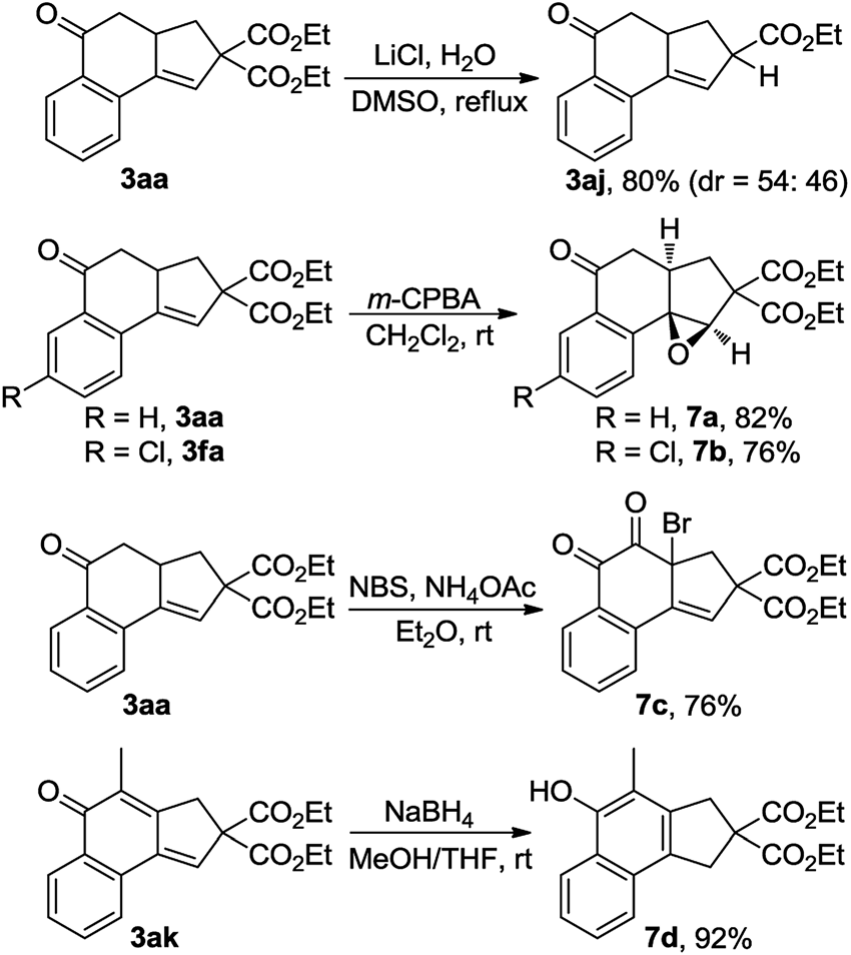
Synthetic utility of cascade annulation.

## Conclusions

We have developed a Cu-catalyzed cascade annulation of enynals with alkenyl or alkynyl α-bromocarbonyls, yielding various cyclohexenone-fused polycyclic compounds under mild reaction conditions. Up to six new C–C bonds and four new rings can be established in a single reaction, highlighting the high efficiency of this protocol. A wide range of functional groups such as F, Cl, OMe, CF_3_, CO_2_Et, Ac, amide, thienyl and alkyl substituents are well tolerated. This reaction represents a novel method for the one-step synthesis of ketones featuring the addition of carbon radicals to aldehydes. Further investigations on the reaction mechanism and application to bioactive ketones are currently underway in our laboratory.

## Supplementary Material

SC-007-C5SC04980F-s001

SC-007-C5SC04980F-s002
