# Supplementary material for: Copper-catalyzed cascade annulation of unsaturated α-bromocarbonyls with enynals: a facile access to ketones from aldehydes
Source: Chem Sci. 2016 Mar 4;7(7):4134–9. doi: 10.1039/c5sc04980f (PMC6014112; doi:10.1039/c5sc04980f)
Supplement: SC-007-C5SC04980F-s001 [file SC-007-C5SC04980F-s001.pdf]

Electronic Supplementary Material (ESI) for Chemical Science.
This journal is © The Royal Society of Chemistry 2016

Supporting Information to

Copper-catalyzed cascade annulation of unsaturated α-bromocarbonyls with enynals: a facile access to ketones from aldehydes

Chao Che, Qianwen Huang, Hanliang Zheng and Gangguo Zhu*

Department of Chemistry, ZhejiangNormalUniversity, 688 Yingbin Road, Jinhua 321004, China

gangguo@zjnu.cn

Table of Contents

General……..……………………...…………………………..…...……………..……..…...….. S2

General Procedure for Experiments and Analytical Data………………….....……..….. S2-S18

NMR Spectra……………………...………………………….....…...……...…...…...…....…..…S19-S62

General. Unless otherwise noted, materials obtained from commercial suppliers were used directly without further purification. ^{1}H, ^{13}C, and ^{19}F NMR spectra were measured on a 600 MHz or 400 MHz NMR spectrometer using $CDCl_3$ as the solvent with tetramethylsilane (TMS) as the internal standard. Chemical shifts (δ) are given in parts per million relative to TMS, and the coupling constants are given in hertz. High-resolution mass spectrometry (HRMS) analyses were carried out using a TOF MS instrument with APCI or ESI source. Column chromatography was performed using silica gel (100−200 mesh).

General Procedure for the Copper-Catalyzed Cascade Annulation of Unsaturated α-Bromocarbonyls with Enynals:

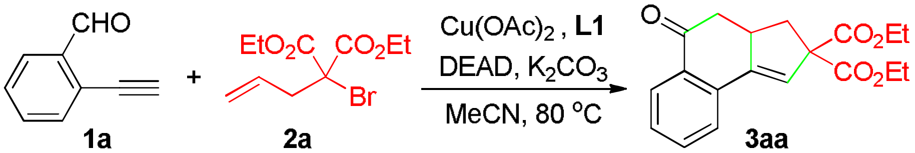

To a mixture of $Cu(OAc)_2$ (4.5 mg, 0.025 mmol), pentamethyldiethylenetriamine (**L1**) (8.7 mg, 0.050 mmol), DEAD (8.7 mg, 0.050 mmol) and K_2CO_3 (34.6 mg, 0.25 mmol) was added a solution of **1a** (32.5 mg, 0.25 mmol) and **2a** (83.4 mg, 0.30 mmol) in 3 mL of MeCN under a nitrogen atmosphere. After stirring at 80 °C for 10 h, the reaction mixture was quenched with water, extracted with EtOAc, washed with brine, dried over anhydrous Na_2SO_4, and concentrated. Column chromatography on silica gel (EtOAc/petroleum ether = 1:10) gave 71 mg of **3aa** (yield: 86%) as a colorless oil. ^{1}H NMR (400 MHz, $CDCl_3$) δ 1.25−1.33 (m, 6H), 2.12 (dd, J = 8.4, 13.3 Hz, 1H), 2.50 (dd, J = 14.0, 15.6 Hz, 1H), 2.96−3.10 (m, 2H), 3.49−3.61 (m, 1H), 4.15−4.30 (m, 4H), 6.37 (d, J = 1.9 Hz, 1H), 7.38−7.45 (m, 1H), 7.53−7.59 (m, 1H), 7.67−7.71 (m, 1H), 7.90−8.10 (m, 1H); ^{13}C NMR (100 MHz, $CDCl_3$) δ 14.0, 14.0, 39.2, 42.3, 45.6, 61.8, 61.8, 66.3, 122.4, 125.5, 127.3, 129.0, 131.1, 133.7, 135.1, 143.7, 167.0, 171.0, 196.9; HRMS (ESI) calcd for $C_{19}H_{21}O_5$ $(M + H)^+$ 329.1389, found 329.1392.

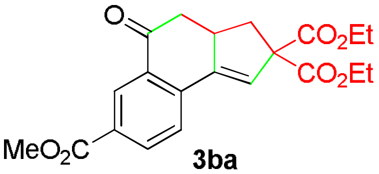

3ba

Compound ***3ba***. 76% yield (73 mg); colorless oil; ^{1}H NMR (600 MHz, $CDCl_3$) δ 1.24−1.30 (m, 6H), 2.12 (dd, J = 8.4, 13.3 Hz, 1H), 2.49 (dd, J = 13.9, 15.8 Hz, 1H), 2.90−3.10 (m, 2H),

3.42−3.59 (m, 1H), 3.91 (s, 3H), 4.13−4.26 (m, 4H), 6.48 (d, J = 2.3 Hz, 1H), 7.72−7.80 (m, 1H), 8.14−8.18 (m, 1H), 8.58−8.61 (m, 1H); ^{13}C NMR (151 MHz, $CDCl_3$) δ 13.9, 13.9, 39.1, 42.0, 45.3, 52.2, 61.8, 61.9, 66.3, 125.0, 125.7, 128.8, 130.5, 130.9, 133.9, 138.6, 142.8, 165.8, 169.5, 170.5, 195.8; HRMS (ESI) calcd for $C_{21}H_{23}O_7$ $(M + H)^+$ 387.1444, found 387.1437.

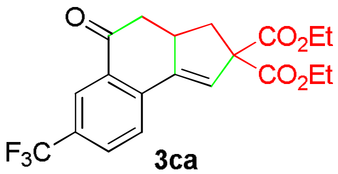

3ca

Compound ***3ca***.75% yield (74 mg); white solid, mp 97–99°C; ^{1}H NMR (600 MHz, $CDCl_3$) δ 1.26−1.33 (m, 6H), 2.16 (dd, J = 8.3, 13.3 Hz, 1H), 2.53 (dd, J = 13.9, 15.9 Hz, 1H), 3.04–3.12 (m, 2H), 3.51–3.59 (m, 1H), 4.18–4.30 (m, 4H), 6.51 (d, J = 2.4 Hz, 1H), 7.75–7.85 (m, 2H), 8.28 (s, 1H); ^{13}C NMR (151 MHz, $CDCl_3$) δ 14.0, 14.0, 39.1, 42.1, 45.3, 62.0,62.0, 66.4, 123.5 (q, J = 272.6 Hz), 124.7 (q, J = 3.9 Hz), 125.2, 126.2, 129.8 (q, J = 3.4 Hz), 131.1 (q, J = 33.2 Hz), 131.2, 138.0, 142.4, 169.6, 170.6, 195.5;^{19}F NMR (565 MHz, $CDCl_3$) δ –63.0;HRMS (ESI) calcd for $C_{20}H_{20}F_3O_5$ $(M + H)^+$ 397.1263, found 397.1255.

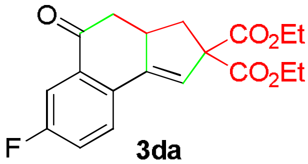

3da

Compound ***3da***. 81% yield (70 mg); white solid, mp 96–98°C; ^{1}H NMR (600 MHz, $CDCl_3$) δ 1.26–1.33 (m, 6H), 2.13 (dd, J = 8.4, 13.3 Hz, 1H), 2.50 (dd, J = 13.9, 15.8 Hz, 1H), 3.01−3.10 (m, 2H), 3.49−3.58 (m, 1H), 4.19−4.30 (m, 4H), 6.33 (d, J = 2.2 Hz, 1H), 7.26−7.30 (m, 1H), 7.60−7.74 (m, 2H); ^{13}C NMR (151 MHz, $CDCl_3$) δ 14.0, 14.0, 39.1, 42.3, 45.3, 61.8, 61.9, 66.2, 113.3 (d, J = 22.4 Hz), 121.3 (d, J = 22.9 Hz), 122.2 (d, J = 2.1 Hz), 127.8 (d, J = 7.6 Hz), 131.5 (d, J = 3.2 Hz), 133.0 (d, J = 6.5 Hz), 142.6 (d, J = 1.0 Hz), 162.9 (d, J = 251.1 Hz),169.9, 170.9, 195.8; ^{19}F NMR (565 MHz, $CDCl_3$) δ–110.2; HRMS (ESI) calcd for $C_{19}H_{20}FO_5$ $(M + H)^+$ 347.1295, found 347.1293.

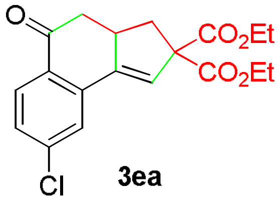

3ea

Compound ***3ea***. 76% yield (69 mg); white solid, mp 141–143°C; ^{1}H NMR (600 MHz, $CDCl_3$) δ

1.27−1.32 (m, 6H), 2.12 (dd, J = 8.3, 13.3 Hz, 1H), 2.48 (dd, J = 13.9, 15.8 Hz, 1H), 2.95−3.08 (m, 2H), 3.46−3.57 (m, 1H), 4.17−4.29 (m, 4H), 6.40 (d, J = 2.3 Hz, 1H), 7.37 (dd, J = 1.9, 8.4 Hz, 1H), 7.67 (d, J = 1.9 Hz, 1H), 7.95 (d, J = 8.4 Hz, 1H); ^{13}C NMR (151 MHz, $CDCl_3$) δ 14.0, 14.0, 39.2, 42.3, 45.4, 61.9, 61.9, 66.3, 123.9, 125.3, 129.1, 129.3, 129.4, 136.5, 140.1, 142.6, 169.8, 170.7, 195.8; HRMS (ESI) calcd for $C_{19}H_{20}ClO_5$ $(M + H)^+$ 363.0999, found 363.0995.

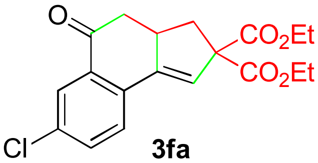

3fa

Compound ***3fa***. 80% yield (73 mg); white solid, mp 136–138°C; ^{1}H NMR (600 MHz, $CDCl_3$) δ 1.26−1.32 (m, 6H), 2.12 (dd, J = 8.4, 13.3 Hz, 1H), 2.48 (dd, J = 13.9, 15.8 Hz, 1H), 2.98−3.09 (m, 2H), 3.46−3.56 (m, 1H), 4.15−4.30 (m, 4H), 6.37 (d, J = 2.3 Hz, 1H), 7.51 (dd, J = 2.3, 8.4 Hz, 1H), 7.64 (d, J = 8.4 Hz, 1H), 7.96 (d, J = 2.2 Hz, 1H); ^{13}C NMR (151 MHz, $CDCl_3$) δ 14.0, 14.0, 39.1, 42.2, 45.3, 61.8, 61.9, 66.3, 123.1, 127.1, 127.1, 132.1, 133.4, 133.6, 135.3, 142.6, 169.8, 170.7, 195.6; HRMS (ESI) calcd for $C_{19}H_{20}ClO_5$ $(M + H)^+$ 363.0999, found 363.0988.

Crystal data for **3fa** ($C_{19}H_{19}ClO_5$, 362.79): triclinic, space group P–1, a = 7.9475(16) Å, b = 10.528(2) Å, c = 11.586(3) Å, U = 911.0(3) Å^3, Z = 2, T = 296(2) K, absorption coefficient 0.235 mm^{-1}, reflections collected 30570, independent reflections 3197 [R(int) = 0.047], refinement by full-matrix least-squares on F^2, data/restraints/parameters 4195/0/214, goodness-of-fit on F^2 = 1.042, final R indices [I>2σ(I)] R_1 = 0.0619, wR_2 = 0.1631, R indices (all data) R_1 = 0.0832, wR_2 = 0.1874, largest diff. peak and hole 0.376 and –0.358e$\cdot$Å^{-3}. Crystallographic data for the structure **3fa** have been deposited with the Cambridge Crystallographic Data Centre as supplementary publication No. CCDC1439849.

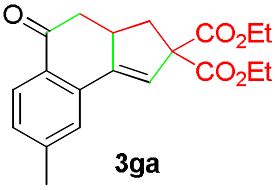

3ga

Compound ***3ga***. 81% yield (69 mg); white solid, mp 91–93°C; ^{1}H NMR (400 MHz, $CDCl_3$) δ 1.26−1.33 (m, 6H), 2.11 (dd, J = 8.4, 13.3 Hz, 1H), 2.40−2.51 (m, 4H), 2.91−3.10 (m, 2H), 3.40−3.60 (m, 1H), 4.15−4.30 (m, 4H), 6.35 (d, J = 2.3 Hz, 1H), 7.22 (d, J = 8.0 Hz, 1H), 7.50 (s, 1H), 7.91 (d, J = 8.0 Hz, 1H); ^{13}C NMR (100 MHz, $CDCl_3$) δ 14.0, 14.0, 21.7, 39.2, 42.4, 45.6,

61.7, 61.8, 66.2, 122.1, 125.7, 127.4, 128.9, 130.1, 135.1, 143.8, 144.5, 170.0, 171.0, 196.7; HRMS (ESI) calcd for $C_{20}H_{23}O_5$ $(M + H)^+$ 343.1545, found 343.1538.

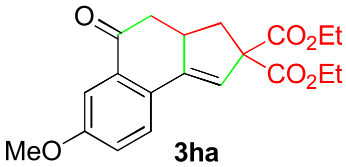

3ha

Compound ***3ha***.90% yield (81 mg); white solid, mp 101–103°C; ^{1}H NMR (600 MHz, $CDCl_3$) δ 1.25−1.32 (m, 6H), 2.09 (dd, J = 8.6, 13.2 Hz, 1H), 2.48 (dd, J = 13.8, 15.7 Hz, 1H), 2.95−3.10 (m, 2H), 3.46−3.55 (m, 1H), 3.87 (s, 3H), 4.13−4.30 (m, 4H), 6.23 (d, J = 2.3 Hz, 1H), 7.13 (dd, J = 2.8, 8.6 Hz, 1H), 7.46 (d, J = 2.8 Hz, 1H), 7.62 (d, J = 8.6 Hz, 1H); ^{13}C NMR (151 MHz, $CDCl_3$) δ 14.0, 14.0, 39.2, 42.4, 45.5, 55.5, 61.7, 61.7, 66.2, 108.9, 120.3, 122.2, 127.1, 128.5, 132.3, 143.3, 160.2, 170.2, 171.1, 196.9; HRMS (ESI) calcd for $C_{20}H_{23}O_6$ $(M + H)^+$ 359.1495, found 359.1489.

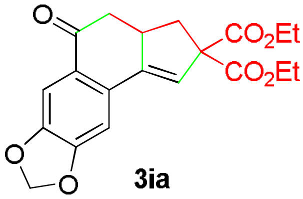

3ia

Compound ***3ia***. 84% yield (78 mg); white solid, mp 135–137°C; ^{1}H NMR (400 MHz, $CDCl_3$) δ 1.24−1.31 (m, 6H), 2.08 (dd, J = 8.5, 13.3 Hz, 1H), 2.43 (dd, J = 13.7, 15.8 Hz, 1H), 2.90−3.10 (m, 2H), 3.40−3.53 (m, 1H), 4.15−4.30 (m, 4H), 6.04 (s, 2H), 6.22 (d, J = 2.3 Hz, 1H), 7.05 (s, 1H), 7.41 (s, 1H); ^{13}C NMR (100 MHz, $CDCl_3$) δ 14.0, 14.0, 39.2, 42.4, 45.2, 61.7, 61.8, 66.1, 101.9, 104.4, 106.2, 121.4, 127.0, 132.1, 143.7, 148.9, 152.3, 170.0, 171.0, 195.3; HRMS (ESI) calcd for $C_{20}H_{21}O_7$ $(M + H)^+$ 373.1287, found 373.1293.

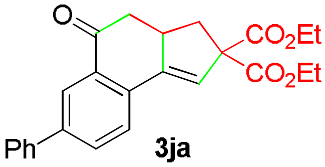

3ja

Compound ***3ja***. 73% yield (74 mg); white solid, mp 81–83°C; ^{1}H NMR (600 MHz, $CDCl_3$) δ 1.26−1.33 (m, 6H), 2.14 (dd, J = 8.5, 13.2 Hz, 1H), 2.54 (dd, J = 14.0, 15.5 Hz, 1H), 3.02−3.10 (m, 2H), 3.52−3.59 (m, 1H), 4.19−4.29 (m, 4H), 6.41 (d, J = 2.3 Hz, 1H), 7.39 (d, J = 7.3 Hz, 1H), 7.46 (t, J = 7.7 Hz, 2H), 7.64 (d, J = 7.4 Hz, 2H), 7.75−7.83 (m, 2H), 8.26 (d, J = 1.8 Hz, 1H); ^{13}C NMR (151 MHz, $CDCl_3$) δ 14.0, 14.1, 39.3, 42.3, 45.7, 61.8, 61.9, 66.3, 122.4, 125.6, 126.1,

127.0, 128.0, 128.9, 131.4, 132.2, 133.9, 139.5, 141.8, 143.4, 170.0, 171.0, 197.0; HRMS (ESI) calcd for $C_{25}H_{25}O_5$ $(M + H)^+$ 405.1702, found 405.1693.

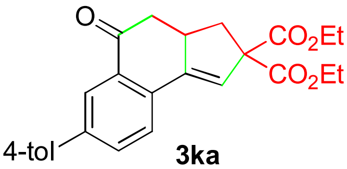

3ka

Compound ***3ka***. 79% yield (83 mg); white solid, mp 87–89°C; ^{1}H NMR (600 MHz, $CDCl_3$) δ 1.26−1.33 (m, 6H), 2.13 (dd, J = 8.5, 13.2 Hz, 1H), 2.40 (s, 3H), 2.50−2.56 (m, 1H), 3.00−3.10 (m, 2H), 3.52−3.59 (m, 1H), 4.17−4.29 (m, 4H), 6.39 (d, J = 2.0 Hz, 1H), 7.27 (d, J = 6.9 Hz, 2H), 7.54 (d, J = 8.0 Hz, 2H), 7.72−7.82 (m, 2H), 8.24 (d, J = 1.5 Hz, 1H); ^{13}C NMR (151 MHz, $CDCl_3$) δ 14.0, 14.1, 21.1, 39.2, 42.3, 45.7, 61.8, 61.8, 66.3, 122.2, 125.2, 126.1, 126.8, 129.6, 131.4, 131.9, 133.7, 136.6, 137.9, 141.7, 143.5, 170.0, 171.0, 197.0; HRMS (ESI) calcd for $C_{26}H_{27}O_5$ $(M + H)^+$ 419.1858, found 419.1850.

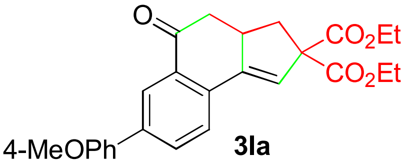

3la

Compound ***3la***. 83% yield (90 mg); white solid, mp 99–101°C; ^{1}H NMR (600 MHz, $CDCl_3$) δ 1.26−1.33 (m, 6H), 2.13 (dd, J = 8.5, 13.2 Hz, 1H), 2.53 (dd, J = 13.9, 15.6 Hz, 1H), 3.00−3.09 (m, 2H), 3.52−3.59 (m, 1H), 3.86 (s, 3H), 4.16−4.29 (m, 4H), 6.38 (d, J = 2.3 Hz, 1H), 6.90−7.01 (m, 2H), 7.57−7.60 (m, 2H), 7.70−7.80 (m, 2H), 8.20−8.22 (m, 1H); ^{13}C NMR (151 MHz, $CDCl_3$) δ 14.0, 14.0, 39.3, 42.4, 45.7, 55.4, 61.8, 61.8, 66.3, 114.4, 122.1, 124.9, 126.1, 128.1, 131.4, 131.7, 132.0, 133.4, 141.5, 143.5, 159.7, 170.1, 171.0, 197.1; HRMS (ESI) calcd for $C_{26}H_{27}O_6$ $(M + H)^+$ 435.1808, found 435.1799.

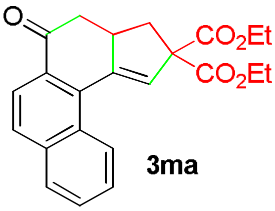

3ma

Compound ***3ma***. 72% yield (68 mg); white solid, mp 133–135°C; ^{1}H NMR (600 MHz, $CDCl_3$) δ 1.27 (t, J = 7.1 Hz, 3H), 1.37 (t, J = 7.1 Hz, 3H), 2.49 (dd, J = 4.3, 14.2 Hz, 1H), 2.72 (dd, J = 13.4, 16.5 Hz, 1H), 2.90 (dd, J = 8.7, 14.2 Hz, 1H), 3.15 (dd, J = 5.8, 16.5 Hz, 1H), 3.60−3.68 (m,

1H), 4.18−4.40 (m, 4H), 6.50 (d, J = 1.5 Hz, 1H), 7.60−7.65 (m, 2H), 7.80−7.89 (m, 2H), 8.00−8.10 (m, 1H), 8.56−8.60 (m, 1H); ^{13}C NMR (151 MHz, CDCl$_3$) δ 14.0, 14.1, 36.4, 45.8, 47.4, 61.9, 62.0, 67.8, 122.7, 127.0, 127.4, 127.7, 128.6, 128.7, 128.9, 129.6, 129.6, 136.0, 136.3, 142.3, 170.8, 171.1, 197.0; HRMS (ESI) calcd for $C_{23}H_{23}O_5$ (M + H)$^+$ 379.1545, found 379.1547.

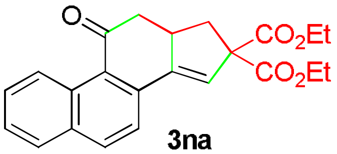

3na

Compound ***3na***. 74% yield (70 mg); white solid, mp 151–153°C; ^{1}H NMR (600 MHz, CDCl$_3$) δ 1.27−1.33 (m, 6H), 2.16 (dd, J = 8.5, 13.1 Hz, 1H), 2.72 (t, J = 14.1 Hz, 1H), 3.01−3.10 (m, 2H), 3.60−3.70 (m, 1H), 4.15−4.30 (m, 4H), 6.48 (d, J = 2.2 Hz, 1H), 7.50−7.53 (m, 1H), 7.61−7.64 (m, 1H), 7.72−7.81 (m, 2H), 7.95−7.97 (m, 1H), 9.30−9.33 (m, 1H); ^{13}C NMR (151 MHz, CDCl$_3$) δ 14.0, 14.0, 39.1, 42.8, 47.9, 61.8, 61.9, 66.3, 122.6, 123.7, 126.1, 126.8, 127.1, 128.3, 129.1, 130.7, 133.9, 134.4, 135.9, 145.1, 169.9, 170.9, 199.7; HRMS (ESI) calcd for $C_{23}H_{23}O_5$ (M + H)$^+$ 379.1545, found 379.1548.

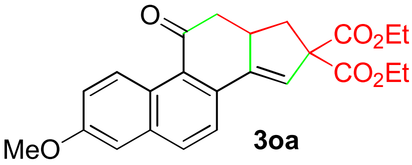

3oa

Compound ***3oa***. 78% yield (80 mg); white solid, mp 176–178°C; ^{1}H NMR (600 MHz, CDCl$_3$) δ 1.27−1.33 (m, 6H), 2.14 (dd, J = 8.5, 13.1 Hz, 1H), 2.71 (t, J = 14.1 Hz, 1H), 3.00−3.08 (m, 2H), 3.60−3.67 (m, 1H), 3.93 (s, 3H), 4.17−4.29 (m, 4H), 6.42 (d, J = 2.2 Hz, 1H), 7.11 (d, J = 2.7 Hz, 1H), 7.26−7.29 (m, 1H), 7.72 (d, J = 8.6 Hz, 1H), 7.87 (d, J = 8.6 Hz, 1H), 9.25 (d, J = 9.5 Hz, 1H); ^{13}C NMR (151 MHz, CDCl$_3$) δ 14.0, 14.1, 39.1, 42.8, 48.0, 55.2, 61.8, 61.8, 66.3, 106.9, 121.0, 122.6, 123.3, 125.9, 126.2, 128.8, 133.2, 133.8, 135.7, 145.1, 158.0, 170.0, 171.0, 199.9; HRMS (ESI) calcd for $C_{24}H_{25}O_6$ (M + H)$^+$ 409.1651, found 409.1642.

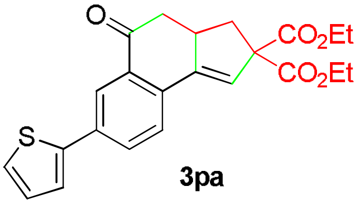

3pa

Compound ***3pa***. 68% yield (70 mg); white solid, mp 133–135°C; ^{1}H NMR (600 MHz, CDCl$_3$) δ 1.26−1.33 (m, 6H), 2.12 (dd, J = 8.5, 13.2 Hz, 1H), 2.49−2.55 (m, 1H), 3.00−3.09 (m, 2H), 3.50−3.57 (m, 1H), 4.16−4.30 (m, 4H), 6.38 (d, J = 2.1 Hz, 1H), 7.11 (dd, J = 3.7, 5.0 Hz, 1H),

7.34 (d, J = 5.1 Hz, 1H), 7.42 (d, J = 3.6 Hz, 1H), 7.70 (d, J = 8.2 Hz, 1H), 7.79 (dd, J = 1.8, 8.2 Hz, 1H), 8.23 (d, J = 1.8 Hz, 1H); ^{13}C NMR (151 MHz, $CDCl_3$) δ 14.0, 14.1, 39.2, 42.3, 45.6, 61.8; 61.9, 66.3, 122.4, 124.0, 124.1, 125.8, 126.2, 128.3, 130.7, 131.4, 133.8, 135.2, 142.8, 143.3, 167.0, 171.0, 196.8; HRMS (ESI) calcd for $C_{23}H_{23}O_5S$ $(M + H)^+$ 411.1266, found 411.1258.

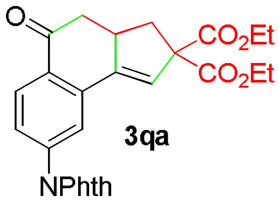

3qa

Compound ***3qa***. 64% yield (76 mg); white solid, mp 64–66°C; ^1H NMR (600 MHz, $CDCl_3$) δ 1.26−1.34 (m, 6H), 2.16 (dd, J = 8.3, 13.3 Hz, 1H), 2.55 (dd, J = 13.9, 15.8 Hz, 1H), 2.99−3.13 (m, 2H), 3.54−3.62 (m, 1H), 4.16–4.31 (m, 4H), 6.44 (d, J = 2.3 Hz, 1H), 7.58 (dd, J = 2.0, 8.4 Hz, 1H), 7.86 (dd, J = 3.1, 5.3 Hz, 3H), 8.01 (dd, J = 3.0, 5.4 Hz, 2H), 8.17 (d, J = 8.4 Hz, 1H); ^{13}C NMR (151 MHz, $CDCl_3$) δ 14.0, 14.1, 39.2, 42.3, 45.5, 61.9, 61.9, 66.4, 122.9, 123.7, 124.0, 126.5, 128.5, 130.0, 131.5, 134.8, 136.1, 136.5, 142.9, 166.7, 169.8, 170.8, 196.0; HRMS (ESI) calcd for $C_{27}H_{24}NO_7$ $(M + H)^+$ 474.1553, found 474.1552.

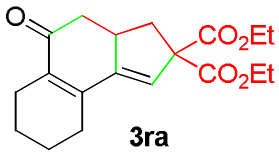

3ra

Compound ***3ra***. 80% yield (66 mg); colorless oil; ^1H NMR (600 MHz, $CDCl_3$) δ 1.24−1.30 (m, 6H), 1.51−1.63 (m, 2H), 1.70−1.80 (m, 2H), 1.96 (dd, J = 9.2, 13.1 Hz, 1H), 2.16–2.21 (m, 1H), 2.28 (dd, J = 13.6, 15.7 Hz, 1H), 2.30−2.50 (m, 2H), 2.52−2.57 (m, 1H), 2.77 (dd, J = 5.9, 15.7 Hz, 1H), 2.93 (dd, J = 7.1, 13.1 Hz, 1H), 3.29−3.36 (m, 1H), 4.13−4.27 (m, 4H), 5.98 (d, J = 1.7 Hz, 1H); ^{13}C NMR (151 MHz, $CDCl_3$) δ 14.0, 14.0, 21.5, 21.6, 22.9, 26.9, 39.3, 41.6, 44.6, 61.7, 61.8, 66.0, 123.3, 135.5, 145.2, 145.7, 169.9, 171.0,198.1; HRMS (ESI) calcd for $C_{19}H_{25}O_5$ $(M + H)^+$ 333.1702, found 333.1697.

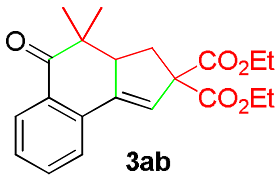

3ab

Compound ***3ab***. 92% yield (82 mg); white solid, mp 86–88°C; ^1H NMR (400 MHz, $CDCl_3$) δ 0.94 (s, 3H), 1.24 (s, 3H), 1.26−1.33 (m, 6H), 2.35 (dd, J = 8.2, 13.8 Hz, 1H), 2.80 (dd, J = 8.2, 13.8

Hz, 1H), 3.34−3.39 (m, 1H), 4.16−4.31 (m, 4H), 6.39 (d, J = 2.4 Hz, 1H), 7.38−7.44 (m, 1H), 7.51−7.56 (m, 1H), 7.66−7.70 (m, 1H), 7.97−8.01 (m, 1H); ^{13}C NMR (100 MHz, $CDCl_3$) δ 14.0, 14.0, 18.9, 21.8, 31.9, 45.9, 51.9, 61.7, 61.8, 66.3, 122.8, 125.0, 128.2, 129.2, 129.9, 133.1, 134.3, 142.4, 170.3, 171.0, 202.5; HRMS (ESI) calcd for $C_{21}H_{25}O_5$ $(M + H)^+$ 357.1702, found 357.1697.

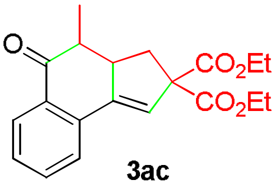

3ac

Compound ***3ac***. 80% yield (69 mg); colorless oil; dr = 88:12; ^{1}H NMR (600 MHz, $CDCl_3$) date of the major isomerδ 1.25−1.33 (m, 9H), 2.14 (dd, J = 8.5, 13.1 Hz, 1H), 2.44−2.52 (m, 1H), 3.07 (dd, J = 7.2, 13.1 Hz, 1H), 3.15−3,21 (m, 1H), 4.15−4.31 (m, 4H), 6.39 (d, J = 2.4 Hz, 1H), 7.38−7.44 (m, 1H), 7.53−7.56 (m, 1H), 7.67−7.71 (m, 1H), 7.97−8.01 (m, 1H); ^{13}C NMR (151 MHz, $CDCl_3$)date of the major isomerδ 12.4, 14.0, 14.0, 38.5, 49.0, 49.1, 61.8, 61.8, 66.0, 122.2, 125.3, 127.5, 129.0, 131.3, 133.3, 134.7, 143.6, 170.0, 171.0, 199.1; HRMS (ESI) calcd for $C_{20}H_{23}O_5$ $(M + H)^+$ 343.1545, found 343.1540.

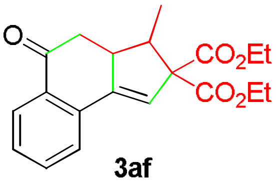

3af

Compound ***3af***. 39% yield (33 mg); colorless oil; dr = 61:39; ^{1}H NMR (600 MHz, $CDCl_3$) date of the major isomerδ 1.22−1.36 (m, 9H), 2.45 (dd, J = 13.8, 15.4 Hz, 1H), 2.62−2.78 (m, 1H), 3.02 (dd, J = 5.5, 15.5 Hz, 1H), 3.08−3.17 (m, 1H), 4.13−4.35 (m, 4H), 6.40 (d, J = 2.2 Hz, 1H), 7.37−7.43 (m, 1H), 7.53−7.58 (m, 1H), 7.71 (t, J = 8.5 Hz, 1H), 7.96−8.01 (m, 1H); ^{13}C NMR (151 MHz, $CDCl_3$) date of the major isomer δ 13.9, 14.1, 14.2, 44.1, 47.9, 48.8, 61.6, 61.6, 69.5, 123.1, 125.2, 127.3, 129.0, 131.1, 133.7, 135.1, 143.4, 169.3, 170.5, 197.2; HRMS (ESI) calcd for $C_{20}H_{22}NaO_5$ $(M + Na)^+$ 365.1365, found 365.1358.

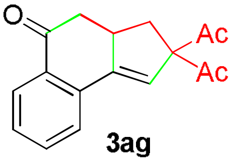

3ag

Compound ***3ag***.76% yield (51 mg); colorless oil; ^{1}H NMR (600 MHz, $CDCl_3$) δ 1.92 (dd, J = 8.5, 13.0 Hz, 1H), 2.22 (s, 3H), 2.23 (s, 3H), 2.46 (dd, J = 13.8, 15.7 Hz, 1H), 3.02 (dd, J = 5.5, 15.7

Hz, 1H), 3.14 (dd, *J* = 7.3, 13.0 Hz, 1H), 3.37−3.44 (m, 1H), 6.56 (d, *J* = 2.4 Hz, 1H), 7.42−7.45 (m, 1H), 7.55−7.60 (m, 1H), 7.70 (d, *J* = 7.5 Hz, 1H), 8.02 (dd, *J* = 1.0, 7.9 Hz, 1H); ^{13}C NMR (151 MHz, $CDCl_3$) *δ* 27.0, 27.1, 37.1, 42.1, 45.7, 80.9, 122.0, 125.3, 127.5, 129.3, 131.2, 133.8, 134.8, 144.3, 196.6, 202.8, 205.2; HRMS (ESI) calcd for $C_{17}H_{17}O_3$ $(M + H)^+$ 269.1178, found 269.1172.

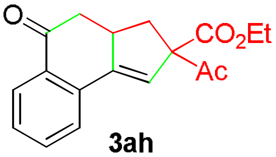

3ah

Compound ***3ah***. 78% yield (58 mg); colorless oil; dr = 55:45; ^{1}H NMR (600 MHz, $CDCl_3$) date of the major isomer *δ* 1.27−1.33 (m, 6H), 2.05−2.11 (m, 1H), 2.27 (s, 3H), 2.43−2.53 (m, 1H), 2.98−3.08 (m, 2H), 3.37−3,46 (m, 1H), 4.20−4.29 (m, 2H), 6.42 (d, *J* = 2.4 Hz, 1H), 7.42 (t, *J* = 7.6 Hz, 1H), 7.57 (t, *J* = 7.5 Hz, 1H), 7.70 (t, *J* = 8.0 Hz, 1H), 8.01 (d, *J* = 7.9 Hz, 1H); ^{13}C NMR (151 MHz, $CDCl_3$) date of the major isomer *δ* 14.1, 26.6, 37.8, 42.2, 45.7, 61.9, 73.2, 122.2, 125.3, 127.4, 129.2, 131.1, 133.7, 135.0, 144.4, 170.4, 196.8, 201.3; HRMS (ESI) calcd for $C_{18}H_{19}O_4$ $(M + H)^+$ 299.1283, found 299.1277.

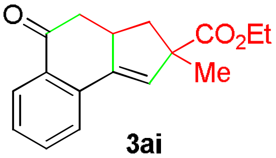

3ai

Compound ***3ai***. 80% yield (54 mg); colorless oil; dr = 60:40; ^{1}H NMR (600 MHz, $CDCl_3$) date of the major isomer *δ* 1.26 (t, *J* = 7.1 Hz, 3H), 1.53 (s, 3H), 2.21 (dd, *J* = 8.7, 12.8 Hz, 1H), 2.42−2.54 (m, 1H), 2.95−3.02 (m, 2H), 3.48−3.55 (m, 1H), 4.15 (q, *J* = 7.1 Hz, 2H), 6.20 (d, *J* = 2.3 Hz, 1H), 7.36−7.41 (m, 1H), 7.53−7.57 (m, 1H), 7.65−7.68 (m, 1H), 8.01 (d, *J* = 7.9 Hz, 1H); ^{13}C NMR (151 MHz, $CDCl_3$) date of the major isomer *δ* 14.1, 25.5, 42.8, 43.6, 46.3, 55.5, 60.8, 125.2, 127.2, 128.4, 129.1, 131.0, 133.5, 135.8, 140.8, 175.6, 197.6; HRMS (ESI) calcd for $C_{17}H_{19}O_3$ $(M + H)^+$ 271.1334, found 271.1327.

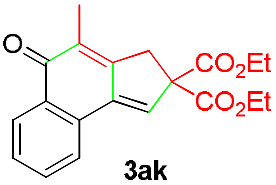

3ak

Compound ***3ak***.74% yield (63 mg); colorless oil; ^{1}H NMR (600 MHz, $CDCl_3$) *δ* 1.32 (t, *J* = 7.1 Hz, 6H), 2.10 (s, 3H), 3.55 (s, 2H), 4.22−4.33 (m, 4H), 7.14 (s, 1H), 7.52−7.61 (m, 2H), 7.88 (d, *J*

= 7.6 Hz, 1H), 8.23 (d, J = 7.7 Hz, 1H); ^{13}C NMR (151 MHz, $CDCl_3$) δ 12.2, 14.0, 36.5, 62.5, 65.5, 124.3, 127.0, 128.7, 129.5, 130.4, 130.7, 131.4, 135.0, 138.4, 154.0, 169.1, 184.5; HRMS (ESI) calcd for $C_{20}H_{21}O_5$$(M + H)^+$ 341.1389, found 341.1383.

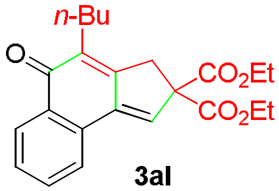

3al

Compound ***3al***.70% yield (67 mg); colorless oil; ^{1}H NMR (600 MHz, $CDCl_3$) δ 0.95 (t, J = 7.3 Hz, 3H), 1.33 (t, J = 7.1 Hz, 6H),1.38−1.46 (m, 2H), 1.49−1.56(m, 2H), 2.53−2.58 (m, 2H), 3.58 (s, 2H), 4.24−4.32 (m, 4H), 7.16 (s, 1H), 7.52−7.62 (m, 2H), 7.89 (d, J = 7.7 Hz, 1H), 8.23 (d, J = 7.8 Hz, 1H); ^{13}C NMR (151 MHz, $CDCl_3$) δ 14.0, 14.0, 22.9, 26.9, 30.6, 36.2, 62.4, 65.5, 124.3, 127.0, 129.5, 130.4, 130.9, 131.4, 133.3, 134.9, 138.6, 153.9, 169.1, 184.0; HRMS (ESI) calcd for $C_{23}H_{27}O_5$ $(M + H)^+$ 383.1858, found 383.1840.

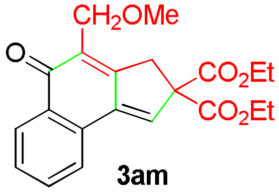

3am

Compound ***3am***. 53% yield (49 mg); colorless oil; ^{1}H NMR (600 MHz, $CDCl_3$) δ 1.31 (t, J = 7.1 Hz, 6H), 3.42 (s, 3H), 3.73 (s, 2H), 4.24− 4.30 (m, 4H), 4.52 (s, 2H), 7.26 (s, 1H), 7.56 (t, J = 7.5 Hz, 1H), 7.53−7.64 (m, 1H), 7.89 (d, J = 7.7 Hz, 1H), 8.24 (d, J = 7.8 Hz, 1H); ^{13}C NMR (151 MHz, $CDCl_3$) δ 14.0, 36.4, 58.6, 62.5, 65.8, 66.3, 124.4, 127.2, 128.4, 129.7, 130.3, 130.6, 131.8, 137.2, 138.7, 158.0, 168.9, 183.5; HRMS (ESI) calcd for $C_{21}H_{23}O_6$ $(M + H)^+$ 371.1495, found 371.1486.

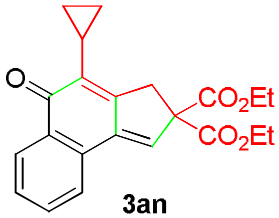

3an

Compound ***3an***. 35% yield (32 mg); colorless oil; ^{1}H NMR (600 MHz, $CDCl_3$) δ 0.90−0.95 (m, 2H), 1.03−1.07 (m, 2H), 1.32 (t, J = 7.1 Hz, 6H), 1.65−1.71 (m, 1H), 3.68 (s, 2H), 4.24−4.32 (m, 4H), 7.13 (s, 1H), 7.52 (t, J = 7.6 Hz, 1H), 7.57 (t, J = 7.5 Hz, 1H), 7.85 (d, J = 7.7 Hz, 1H), 8.17 (d, J = 7.8 Hz, 1H); ^{13}C NMR (151 MHz, $CDCl_3$) δ 6.0, 10.5, 14.0, 36.5, 62.4, 65.5, 124.2, 126.8,

129.5, 130.1, 131.3, 131.3, 132.3, 134.5, 138.7, 154.9, 169.2, 184.3; HRMS (ESI) calcd for $C_{22}H_{22}O_5$ $(M + H)^+$ 367.1545, found 367.1537.

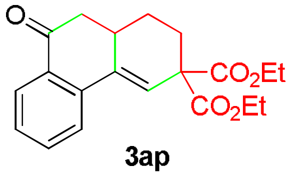

3ap

Compound ***3ap***. 81% yield (69 mg); white solid, mp 158–161°C; ^{1}H NMR (600 MHz, $CDCl_3$) δ 1.27−1.34 (m, 6H), 1.61−1.69 (m, 1H), 1.88−1.94(m, 1H), 2.00−2.14 (m, 1H), 2.39−2.46(m, 1H), 2.52−2.56 (m, 1H), 2.80−2.89 (m, 2H), 4.16−4.32 (m, 4H), 6.69 (s, 1H), 7.42 (t, J = 7.5 Hz, 1H), 7.55−7.62 (m, 1H), 7.77 (d, J = 8.0 Hz, 1H), 8.06 (d, J = 7.8 Hz, 1H); ^{13}C NMR (151 MHz, $CDCl_3$) δ 14.0, 14.1, 27.2, 27.2, 34.7, 45.4, 55.6, 61.8, 61.9, 121.8, 124.8, 126.9, 128.5, 131.3, 133.8, 138.0, 140.1, 170.0, 170.8, 197.0; HRMS (ESI) calcd for $C_{20}H_{23}O_5$ $(M + H)^+$ 343.1545, found 343.1542.

Crystal data for **3ap** ($C_{20}H_{22}O_5$, 342.38): triclinic, space group P–1, a = 8.1720(19) Å, b = 9.085(2) Å, c = 13.302(3) Å, U = 867.4(4) Å^3, Z = 2, T = 296(2) K, absorption coefficient mm^{-1}, reflections collected 5137, independent reflections 2385 [R(int) = 0.039], refinement by full-matrix least-squares on F^2, data/restraints/parameters 3166/13/238, goodness-of-fit on F^2 = 1.076, final R indices [I>2σ(I)] R_1 = 0.0687, wR_2 = 0.1835, R indices (all data) R_1 = 0.0787, wR_2 = 0.1891, largest diff. peak and hole 0.284 and –0.306e$^{\cdot}$ Å^{-3}. Crystallographic data for the structure **3ap** have been deposited with the Cambridge Crystallographic Data Centre as supplementary publication No. CCDC1439850.

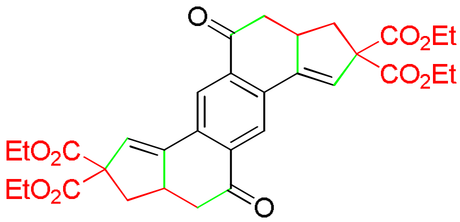

3sa

Compound ***3sa***. It was prepared from **1s** and **2a** in 47% yield (68 mg) using the general procedure except that 6 equivalents of K_2CO_3 was used; pale yellow solid, mp 59–61°C; ^{1}H NMR (600 MHz, $CDCl_3$) δ 1.28−1.33 (m, 12H), 2.09−2.15 (m, 2H), 2.48−2.56 (m, 2H), 3.00−3.11 (m, 4H), 3.48−3.55 (m, 2H), 4.15−4.29 (m, 8H), 6.53 (dd, J = 2.3, 6.3 Hz, 2H), 8.32 (s, 2H); ^{13}C NMR (151 MHz, $CDCl_3$) δ 14.0, 14.1, 14.4, 39.2, 39.2, 42.0, 42.0, 45.5, 45.6, 61.9, 61.9, 62.0, 62.0, 66.4,

66.4, 124.4, 124.5, 125.0, 125.1, 133.9, 134.0, 135.0, 135.0, 142.4, 169.7, 169.7, 170.7, 170.7, 196.4, 196.4; HRMS (ESI) calcd for $C_{32}H_{35}O_{10}$ $(M + H)^+$ 579.2230, found 579.2249.

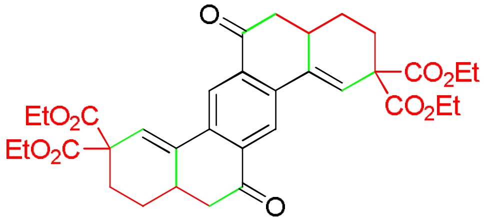

3sp

Compound ***3sp***. It was prepared from **1s** and **2p** in 38% yield (58 mg) using the general procedure except that 6 equivalents of K_2CO_3 was used; pale yellow solid, mp 66–68°C; ^{1}H NMR (600 MHz, $CDCl_3$) δ 1.27−1.33 (m, 12H), 1.62−1.70 (m, 4H), 1.92−2.00 (m, 2H), 2.10−2.16 (m, 2H), 2.43−2.56 (m, 2H), 2.83−2.94 (m, 4H), 4.23 (d, J = 7.1 Hz, 2H), 4.30 (m, 6H), 6.75−2.86 (m, 2H), 8.42 (d, J = 5.9 Hz, 2H); ^{13}C NMR (151 MHz, $CDCl_3$) δ 14.0, 14.0, 14.4, 27.2, 27.2, 34.4, 34.4, 45.3, 45.4, 55.8, 55.9, 61.9, 61.9, 62.0, 62.3, 123.0, 123.1, 123.8, 123.9, 134.3, 134.3, 136.7, 136.9, 139.2, 139.4, 167.0, 167.0, 170.5, 170.5, 196.7, 196.7; HRMS (ESI) calcd for $C_{32}H_{39}O_{10}$ $(M + H)^+$ 607.2543, found 607.2540.

Experimental Procedure for the Synthesis of 4a from 1a and 2a:

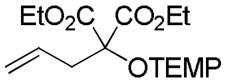

4a

Compound ***4a***. It was obtained from **1a** and **2a** under the reaction conditions in the presence of 2 equivalents of TEMPO in 51% yield (55 mg) as a colorless oil; ^{1}H NMR (600 MHz, $CDCl_3$) δ 1.15 (d, J = 2.0 Hz, 6H), 1.21 (d, J = 1.9 Hz, 6H), 1.29 (td, J = 2.2, 7.1 Hz, 6H), 1.31−1.62 (m, 6H), 2.99 (d, J = 7.2 Hz, 2H), 4.16−4.25 (m, 4H), 5.05 (dd, J = 13.6, 22.2 Hz, 2H), 5.86−5.97 (m, 1H); ^{13}C NMR (151 MHz, $CDCl_3$) δ 13.5, 13.5, 16.4, 20.2, 20.2, 32.7, 32.7, 38.3, 40.6, 40.6, 60.3, 60.6, 60.6, 88.1, 117.3, 132.8, 168.6; HRMS (ESI) calcd for $C_{19}H_{34}NO_5$ $(M + H)^+$ 356.2437, found 356.2433.

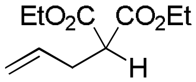

4b

*Compound **4b***[1]. It was obtained from **1a** and **2a** under the reaction conditions in the presence of 2 equivalents of BHT in 35% yield (18 mg) as a colorless oil; ^{1}H NMR (600 MHz, $CDCl_3$) δ1.27 (t, J = 7.1 Hz, 6H), 2.65 (t, J = 7.2 Hz, 2H), 3.43 (t, J = 7.6 Hz, 1H), 4.16−4.24 (m, 4H), 5.03−5.17 (m, 2H), 5.72−5.83 (m, 1H); ^{13}C NMR (151 MHz, $CDCl_3$) δ 14.0, 32.8, 51.6, 61.4, 117.5, 134.0, 168.9.

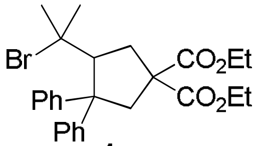

4c

*Compound **4c***. It was obtained from **1a** and **2b** under the reaction conditions in the presence of 2 equivalents of 1,1-diphenylethylene in 68% yield (83 mg) as a colorless oil; ^{1}H NMR (600 MHz, $CDCl_3$) δ 0.78 (s, 3H), 1.15 (t, J = 7.1 Hz, 3H), 1.25−1.32 (m, 6H), 2.05 (dd, J = 10.4, 13.3 Hz, 1H), 2.56 (dd, J = 7.6, 13.4 Hz, 1H), 2.95−3.01 (m, 2H), 3.21 (d, J = 14.7 Hz, 1H), 3.97 (q, J = 7.1 Hz, 2H), 4.23−4.33 (m, 2H), 7.08−7.38 (m, 10H); ^{13}C NMR (151 MHz, $CDCl_3$) δ 13.9, 14.0, 23.9, 30.8, 37.8, 45.1, 50.5, 61.3, 61.4, 61.6, 62.7, 65.3, 123.0, 125.7, 126.6, 126.7, 127.0, 127.7, 128.0, 146.1, 148.8, 150.9, 171.3, 172.6; HRMS (ESI) calcd for $C_{26}H_{31}O_4$ $(M - Br)^+$ 407.2222, found 407.2222.

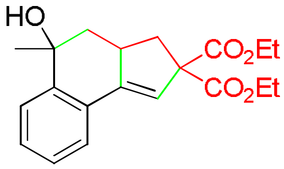

6a

Compound ***6a***. 62% yield (53 mg); colorless oil; dr = 68:32; ^{1}H NMR (600 MHz, $CDCl_3$) date of the major isomer δ 1.24−1.30 (m, 6H), 1.65 (s, 3H), 1.76 (m, 1H), 1.92−2.00 (m, 2H), 2.21−2.27 (m, 1H), 2.93−3.00 (m, 1H), 3.34−3.41 (m, 1H), 4.13−4.26 (m, 4H), 6.17−6.19 (d, J = 2.4 Hz, 1H), 7.25−7.34 (m, 2H), 7.55−7.59 (m, 1H), 7.62−7.66 (m, 1H); ^{13}C NMR (151 MHz, $CDCl_3$) date of the major isomer δ 14.0, 29.7, 38.5, 41.4, 45.2, 61.5, 65.8, 70.3, 119.6, 125.6, 126.2, 127.9, 129.1, 140.7, 146.3, 170.6, 171.6; HRMS (ESI) calcd for $C_{20}H_{25}O_5$ $(M + H)^+$ 345.1702, found 345.1697.

Experimental Procedure for the Preparation of 6b via Reduction of 3aa:

[1]K.-T. Yip, N.-Y. Zhu and D. Yang, *Org. Lett.*, 2009, **11**, 1911.

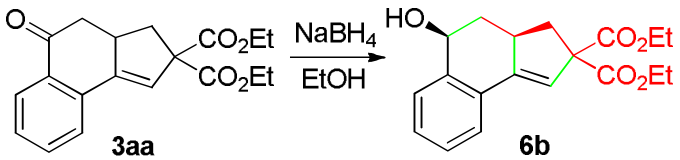

To a solution of **3aa** (164 mg, 0.5mmol) in 3 mL of EtOH was added $NaBH_4$ (28 mg, 0.75 mmol). After stirring at room temperature for 30 min, the reaction mixture was quenched with water, extracted with EtOAc, washed with brine, dried over anhydrous Na_2SO_4, and concentrated. Column chromatography on silica gel (EtOAc/petroleum ether = 1:5) gave 145 mg of **6b** (yield: 88%) as a colorless oil; the stereochemistry of **6b** was determined by the NOE measurements. ^{1}H NMR (600 MHz, $CDCl_3$) δ 1.25−1.32 (m, 6H), 1.48−1.56 (m, 1H), 1.96−2.07 (m, 2H), 2.46−2.50 (m, 1H), 2.96 (dd, J = 7.0, 12.8 Hz, 1H), 3.15−3.23 (m, 1H), 4.14−4.30 (m, 4H), 4.89−4.92 (m, 1H), 6.19 (d, J = 2.3 Hz, 1H), 7.24−7.29 (m, 1H), 7.34 (t, J = 7.5 Hz, 1H), 7.65 (dd, J = 7.7, 15.7 Hz, 2H); ^{13}C NMR (151 MHz, $CDCl_3$) δ 14.0, 14.1, 39.0, 40.5, 41.6, 61.5, 61.6, 65.7, 69.5, 119.0, 125.0, 126.3, 127.4, 128.9, 129.3, 140.4, 145.9, 170.4, 171.5; HRMS (ESI) calcd for $C_{19}H_{22}NaO_5$ $(M + Na)^+$ 353.1365, found 353.1371.

Experimental Procedure for the Synthesis of 3aj from 3aa:[2]

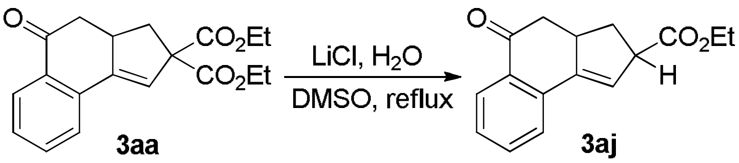

A mixture of **3aa** (164 mg, 0.5mmol), LiCl (63 mg, 1.5mmol), and H_2O (0.25 mL) in 1 mL of dimethyl sulfoxide was heated at reflux for 5 h. After cooling to room temperature, the reaction mixture was quenched with water, extracted with EtOAc, washed with brine, dried over anhydrous Na_2SO_4, and concentrated. Column chromatography on silica gel (EtOAc/petroleum ether = 1:15) gave 103 mg of **3aj** (yield: 80%) as a colorless oil; dr = 54:46; ^{1}H NMR (600 MHz, $CDCl_3$) date of the major isomer δ 1.28−1.35 (m, 3H), 1.91−2.00 (m, 1H), 2.56 (dd, J = 13.8, 15.7 Hz, 1H), 2.62−2.69 (m, 1H), 3.00−3.05 (m, 1H), 3.32−3.39 (m, 1H), 3.79−3.84 (m, 1H), 4.20−4.26 (m, 2H), 6.32−6.36 (m, 1H), 7.40 (dd, J = 7.1, 14.3 Hz, 1H), 7.53−7.58 (m, 1H), 7.65−7.73 (m, 1H), 8.00−8.05 (m, 1H); ^{13}C NMR (151 MHz, $CDCl_3$) date of the major isomer δ 14.2, 34.6, 42.9, 46.0, 50.6, 61.0, 123.3, 125.0, 127.3, 128.5, 130.8, 133.6, 135.7, 141.6, 173.8, 197.6; HRMS (ESI)

[2]A. P. Krapcho, J. F. Weimaster, J. M. Eldridge, E. G. E. Jahngen, Jr., A. J. Lovey and W. P. Stephens, *J. Org. Chem.,*1978, **43**, 138.

calcd for $C_{16}H_{17}O_3$ $(M + H)^+$ 257.1178, found 257.1174.

Experimental Procedure for the Epoxidation of Tricyclic Ketones:

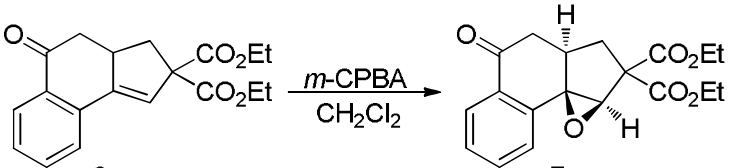

To a mixture of **3aa** (164 mg, 0.5mmol) and $NaHCO_3$ (84 mg, 1.0 mmol) in 5 mL of DCM was added *m*-CPBA (172 mg, 1.0mmol) at 0°C. After stirring at 25°C for 24 h, the reaction mixture was quenched with water, extracted with EtOAc, washed with brine, dried over anhydrous Na_2SO_4, and concentrated. Column chromatography on silica gel (EtOAc/petroleum ether = 1:15) gave 141 mg of **7a** (yield: 82%) as a colorless oil; ^{1}H NMR (600 MHz, $CDCl_3$) δ 1.27 (t, J = 7.1 Hz, 3H), 1.34 (t, J = 7.1 Hz, 3H), 1.95−2.00 (m, 1H), 2.36−2.40 (m, 1H), 2.75−2.84 (m, 2H), 2.90−2.98 (m, 1H), 4.19−4.35 (m, 4H), 4.74 (s, 1H), 7.24−7.29 (m, 1H), 7.50−7.62 (m, 2H), 8.05−8.13 (m, 1H); ^{13}C NMR (151 MHz, $CDCl_3$) δ 14.0, 14.0, 32.5, 36.2, 40.3, 60.9, 62.0, 62.1, 63.4, 64.9, 125.8, 127.9, 129.7, 134.0, 134.8, 135.7, 168.4, 168.8, 196.2; HRMS (ESI) calcd for $C_{19}H_{21}O_6$ $(M + H)^+$ 345.1338, found 345.1333.

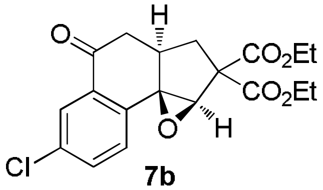

7b

Compound ***7b***. 76% yield (144 mg); white solid, mp 83–85°C; ^{1}H NMR (600 MHz, $CDCl_3$) δ 1.27 (t, J = 7.1 Hz, 3H), 1.34 (t, J = 7.1 Hz, 3H), 1.94−2.00 (m, 1H), 2.35−2.41 (m, 1H), 2.73−2.82 (m, 2H), 2.90−3.00 (m, 1H), 4.19−4.37 (m, 4H), 4.71 (s, 1H), 7.21 (d, J = 8.2 Hz, 1H), 7.56 (dd, J = 2.2, 8.2 Hz, 1H), 8.07 (d, J = 2.2 Hz, 1H); ^{13}C NMR (151 MHz, $CDCl_3$) δ 13.9, 14.0, 32.3, 36.0, 40.1, 60.8, 62.1, 62.1, 63.5, 64.3, 127.5, 127.9, 134.0, 134.0, 136.1, 136.3, 168.2, 168.7, 194.8; HRMS (ESI) calcd for $C_{19}H_{20}ClO_6$ $(M + H)^+$ 379.0948, found 379.0952.

Crystal data for **7b** ($C_{19}H_{19}ClO_6$, 378.79): triclinic, space group P–1, a = 9.9793(12) Å, b = 11.9540(15) Å, c = 15.515(2) Å, U = 1814.8(4) Å^3, Z = 4, T = 296(2) K, absorption coefficient 0.243mm^{-1}, reflections collected 8218, independent reflections 4660 [R(int) = 0.0639], refinement by full-matrix least-squares on F^2, data/restraints/parameters 8218/26/470, goodness-of-fit on F^2 =

1.017, final R indices [$I > 2\sigma(I)$] R_1 = 0.0720, wR_2 = 0.1765, R indices (all data) R_1 = 0.1273, wR_2 = 0.2177, largest diff. peak and hole 0.870 and –0.352 e·Å^{-3}. Crystallographic data for the structure **7b** have been deposited with the Cambridge Crystallographic Data Centre as supplementary publication No. CCDC1439851.

Experimental Procedure for the Synthesis of α-BromoDiketone 7c:[3]

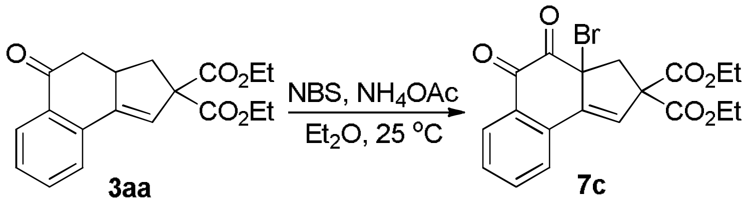

To a solution of **3aa** (82 mg, 0.25 mmol) in 1 mL of Et_2O was added NBS (445 mg, 2.5 mmol) and NH_4OAc (19 mg, 0.25 mmol). After stirring at room temperature for 1.5 h, the reaction mixture was quenched with water, extracted with EtOAc, washed with brine, dried over anhydrous Na_2SO_4, and concentrated. Column chromatography on silica gel (EtOAc/petroleum ether = 1:15) gave 80 mg of **7c** (yield: 76%) as a colorless oil; ^{1}H NMR (600 MHz, $CDCl_3$) δ 1.24−1.28 (m, 3H), 1.34−1.38 (m, 3H), 3.30 (d, J = 18.2 Hz, 1H), 3.72 (d, J = 18.2 Hz, 1H), 4.11−4.38 (m, 4H), 5.95 (s, 1H), 7.56 (t, J = 6.2 Hz, 2H), 7.73 (t, J = 7.1 Hz, 1H), 8.11−8.15 (m, 1H); ^{13}C NMR (151 MHz, $CDCl_3$) δ 13.8, 14.0, 34.7, 51.8, 62.6, 62.9, 64.7, 126.6, 130.1, 130.8, 131.0, 131.4, 135.6, 135.7, 153.8, 166.2, 168.7, 178.2, 179.1; HRMS (ESI) calcd for $C_{19}H_{18}BrO_6$ $(M + H)^+$ 421.0287, found 421.0274.

Experimental Procedure for the Preparation of 1-Naphthol 7d via Reduction of 3ak:

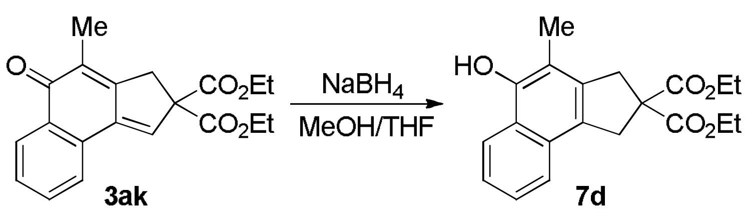

To a solution of **3ak** (170 mg, 0.5mmol) in 2 mL of MeOH/THF (v/v = 1:1) was added $NaBH_4$ (23 mg, 0.6 mmol). After stirring at 25°C for 30 min, the reaction mixture was quenched with water, extracted with EtOAc, washed with brine, dried over anhydrous Na_2SO_4, and concentrated.

[3] K. Tanemura, T. Suzuki, Y. Nishida, K. Satsumabayashi and T. Horaguchi, *Chem. Commun.,*2004, 470.

Column chromatography on silica gel (EtOAc/petroleum ether = 1:10) gave 154 mg of **7d** (yield: 92%) as a colorless oil; ^{1}H NMR (600 MHz, $CDCl_3$) δ 1.28 (t, J = 7.1 Hz, 6H), 2.28 (s, 3H), 3.69 (s, 2H), 3.89 (s, 2H), 4.24 (q, J = 7.1 Hz, 4H), 5.23 (br,s, 1H), 7.40–7.46 (m, 2H), 7.66 (d, J = 7.6 Hz, 1H), 8.07 (d, J = 7.8 Hz, 1H); ^{13}C NMR (151 MHz, $CDCl_3$) δ 12.3, 14.0, 39.2, 40.8, 59.6, 61.8, 113.5, 121.7, 123.9, 124.0, 124.5, 125.7, 126.8, 128.8, 137.5, 148.2, 172.0; HRMS (ESI) calcd for $C_{20}H_{23}O_5$ $(M + H)^+$ 343.1545, found 343.1535.

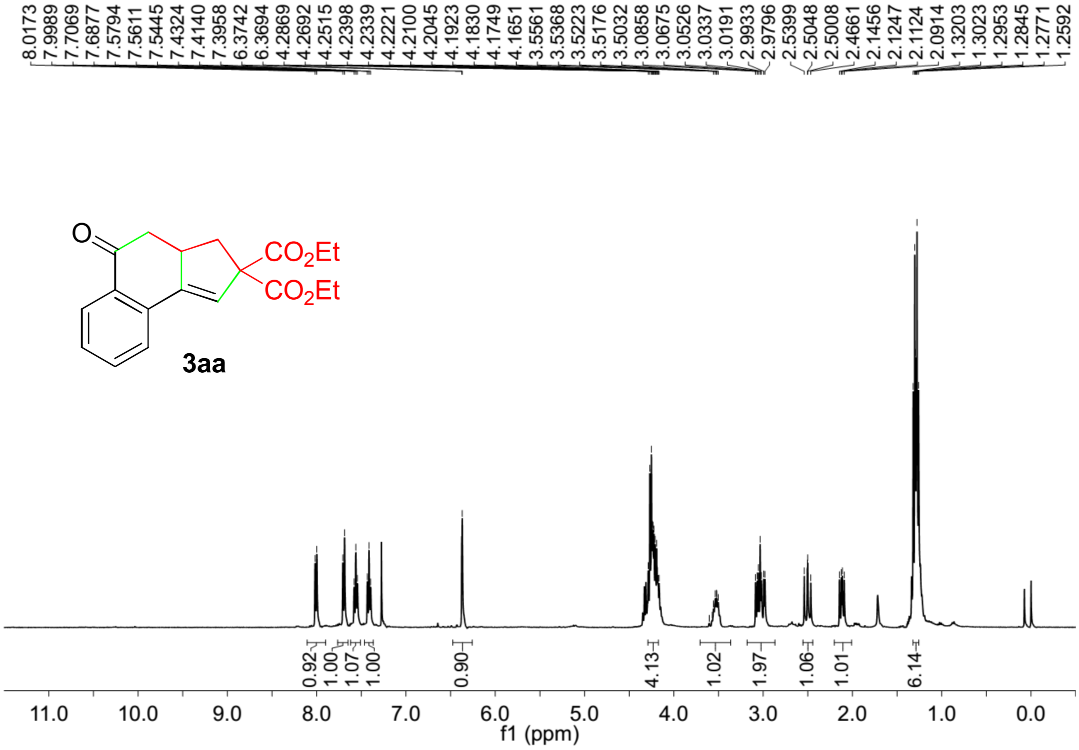

8.0173
7.9989
7.7069
7.6877
7.5794
7.5611
7.5445
7.4324
7.4140
7.3958
6.3742
6.3694
4.2869
4.2692
4.2515
4.2398
4.2339
4.2221
4.2100
4.2045
4.1923
4.1830
4.1749
4.1651
3.5561
3.5368
3.5223
3.5176
3.5032
3.0858
3.0675
3.0526
3.0337
3.0191
2.9933
2.9796
2.5399
2.5048
2.5008
2.4661
2.1456
2.1247
2.1124
2.0914
1.3203
1.3023
1.2953
1.2845
1.2771
1.2592
O
CO2Et
CO2Et
3aa
0.92
1.00
1.07
1.00
0.90
4.13
1.02
1.97
1.06
1.01
6.14
11.0
10.0
9.0
8.0
7.0
6.0
5.0
4.0
3.0
2.0
1.0
0.0
f1 (ppm)

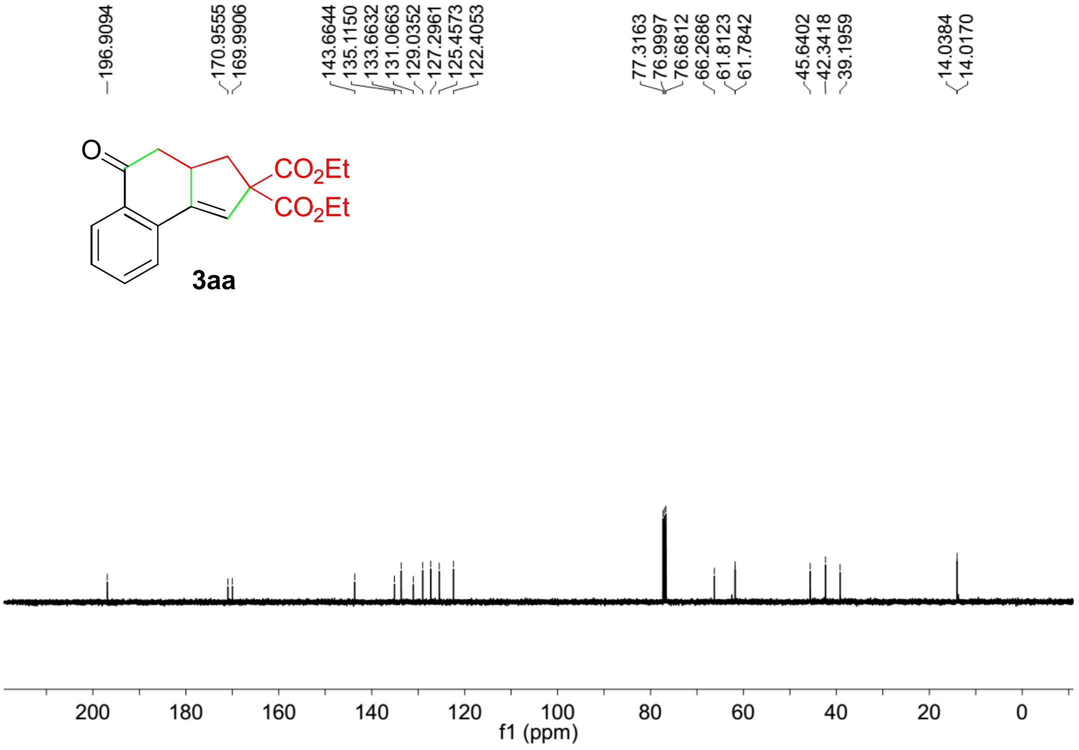

196.9094
170.9555
169.9906
143.6644
135.1150
133.6632
131.0663
129.0352
127.2961
125.4573
122.4053
77.3163
76.9997
76.6812
66.2686
61.8123
61.7842
45.6402
42.3418
39.1959
14.0384
14.0170
O
CO2Et
CO2Et
3aa
200
180
160
140
120
100
80
60
40
20
0
f1 (ppm)

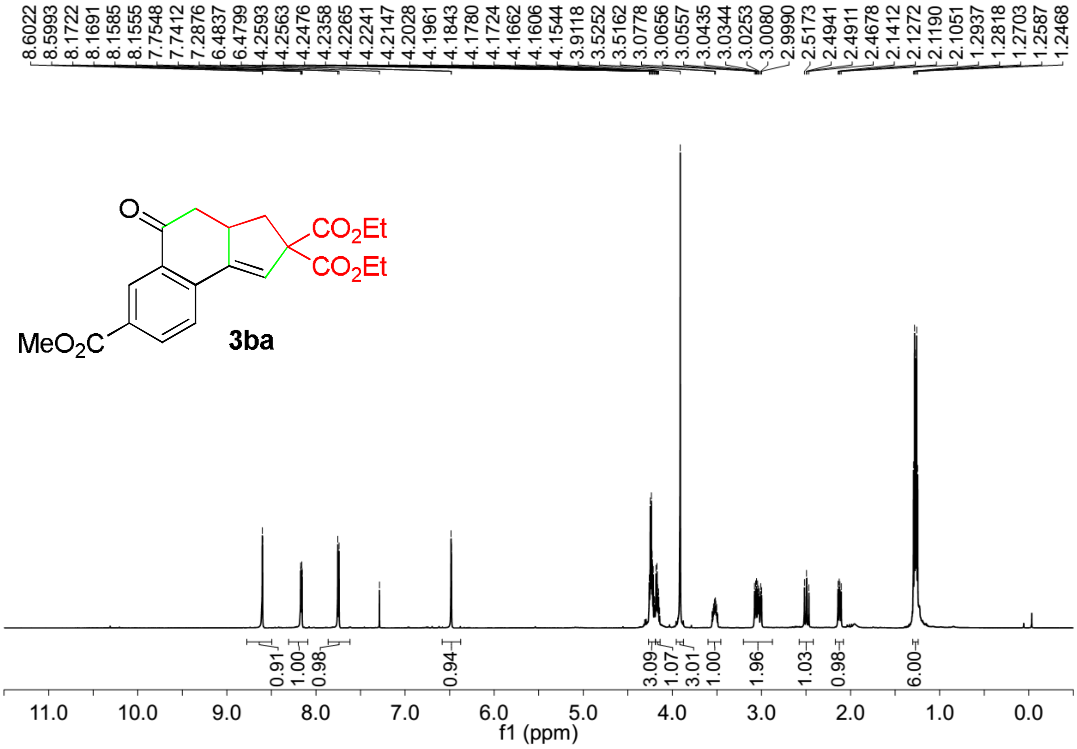
8.6022 8.5993 8.1722 8.1691 8.1585 8.1555 7.7548 7.7412 7.2876 6.4837 6.4799 4.2593 4.2563 4.2476 4.2358 4.2265 4.2241 4.2147 4.2028 4.1961 4.1843 4.1780 4.1724 4.1662 4.1606 4.1544 3.9118 3.5252 3.5162 3.0778 3.0656 3.0557 3.0435 3.0344 3.0253 3.0080 2.9990 2.5173 2.4941 2.4911 2.4678 2.1412 2.1272 2.1190 2.1051 1.2937 1.2818 1.2703 1.2587 1.2468
O
CO_2Et
CO_2Et
MeO_2C
3ba
0.91 1.00 0.98 0.94 3.09 1.07 3.01 1.00 1.96 1.03 0.98 6.00
11.0 10.0 9.0 8.0 7.0 6.0 5.0 4.0 3.0 2.0 1.0 0.0
f1 (ppm)

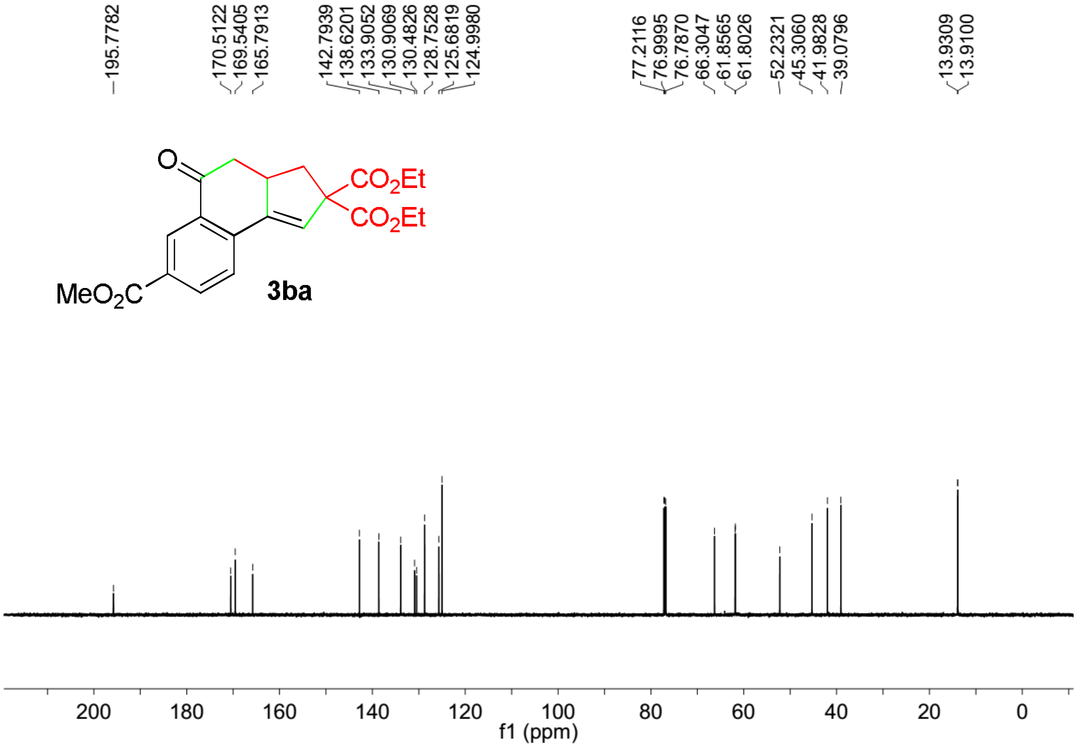
195.7782 170.5122 169.5405 165.7913 142.7939 138.6201 133.9052 130.9069 130.4826 128.7528 125.6819 124.9980 77.2116 76.9995 76.7870 66.3047 61.8565 61.8026 52.2321 45.3060 41.9828 39.0796 13.9309 13.9100
O
CO_2Et
CO_2Et
MeO_2C
3ba
200 180 160 140 120 100 80 60 40 20 0
f1 (ppm)

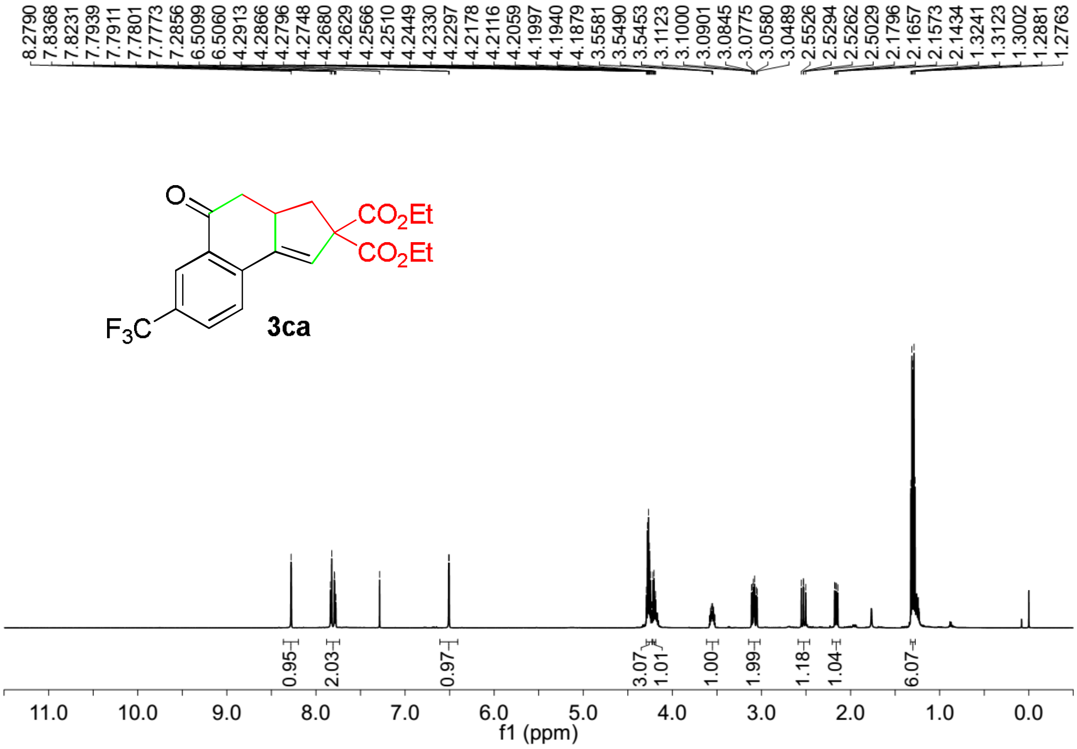
8.2790 7.8368 7.8231 7.7939 7.7911 7.7801 7.7773 7.2856 6.5099 6.5060 4.2913 4.2866 4.2796 4.2748 4.2680 4.2629 4.2566 4.2510 4.2449 4.2330 4.2297 4.2178 4.2116 4.2059 4.1997 4.1940 4.1879 3.5581 3.5490 3.5453 3.1123 3.1000 3.0901 3.0845 3.0775 3.0580 3.0489 2.5526 2.5294 2.5262 2.5029 2.1796 2.1657 2.1573 2.1434 1.3241 1.3123 1.3002 1.2881 1.2763
O
CO_2Et
CO_2Et
F_3C
3ca
0.95
2.03
0.97
3.07
1.01
1.00
1.99
1.18
1.04
6.07
11.0 10.0 9.0 8.0 7.0 6.0 5.0 4.0 3.0 2.0 1.0 0.0
f1 (ppm)

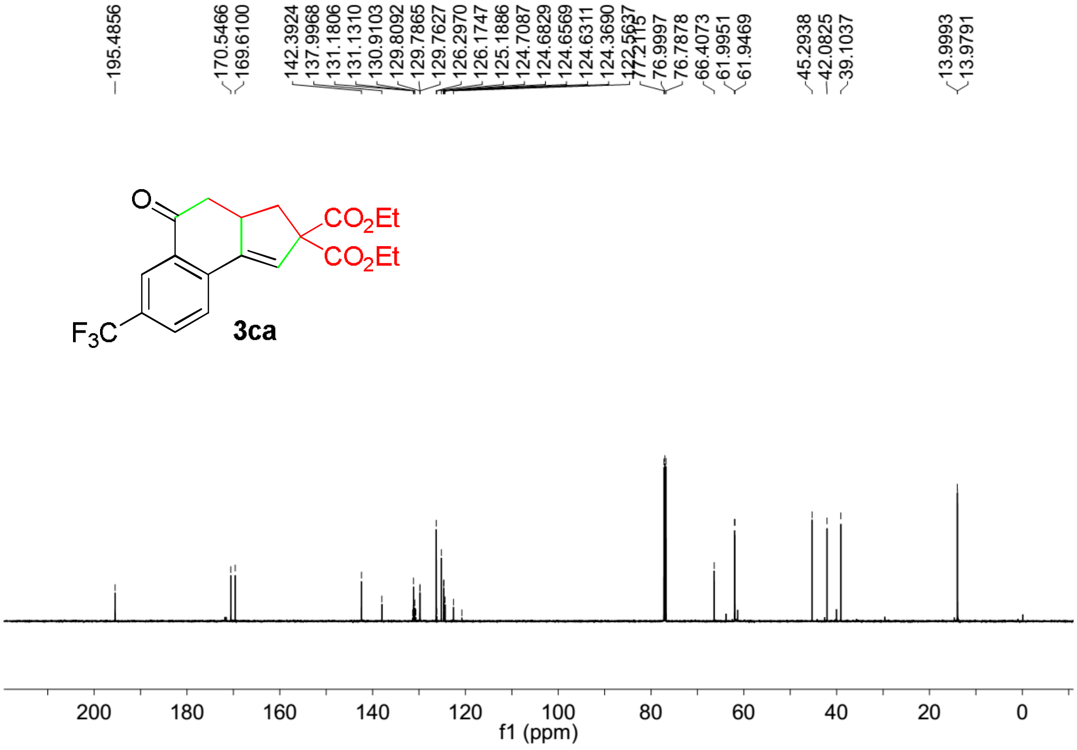
195.4856
170.5466 169.6100
142.3924 137.9968 131.1806 131.1310 130.9103 129.8092 129.7865 129.7627 126.2970 126.1747 125.1886 124.7087 124.6829 124.6569 124.6311 124.3690 122.5637 77.2115 76.9997 76.7878
66.4073 61.9951 61.9469
45.2938 42.0825 39.1037
13.9993 13.9791
O
CO_2Et
CO_2Et
F_3C
3ca
200 180 160 140 120 100 80 60 40 20 0
f1 (ppm)

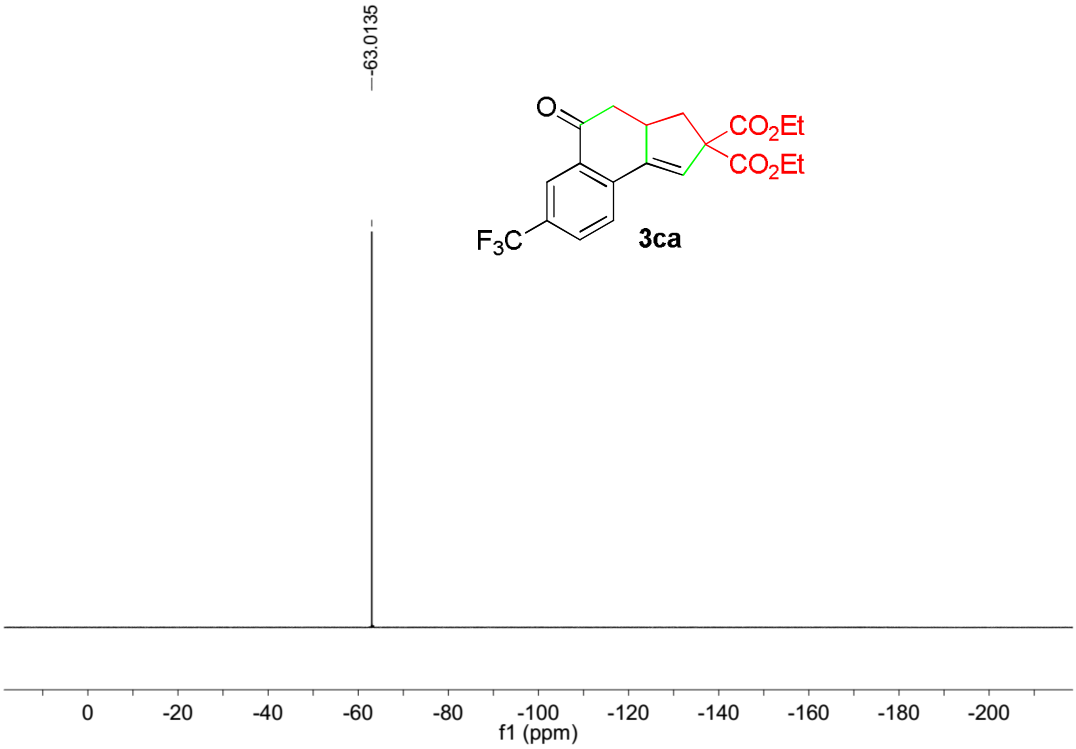

-63.0135
O
CO2Et
CO2Et
F3C
3ca
0
-20
-40
-60
-80
-100
-120
-140
-160
-180
-200
f1 (ppm)

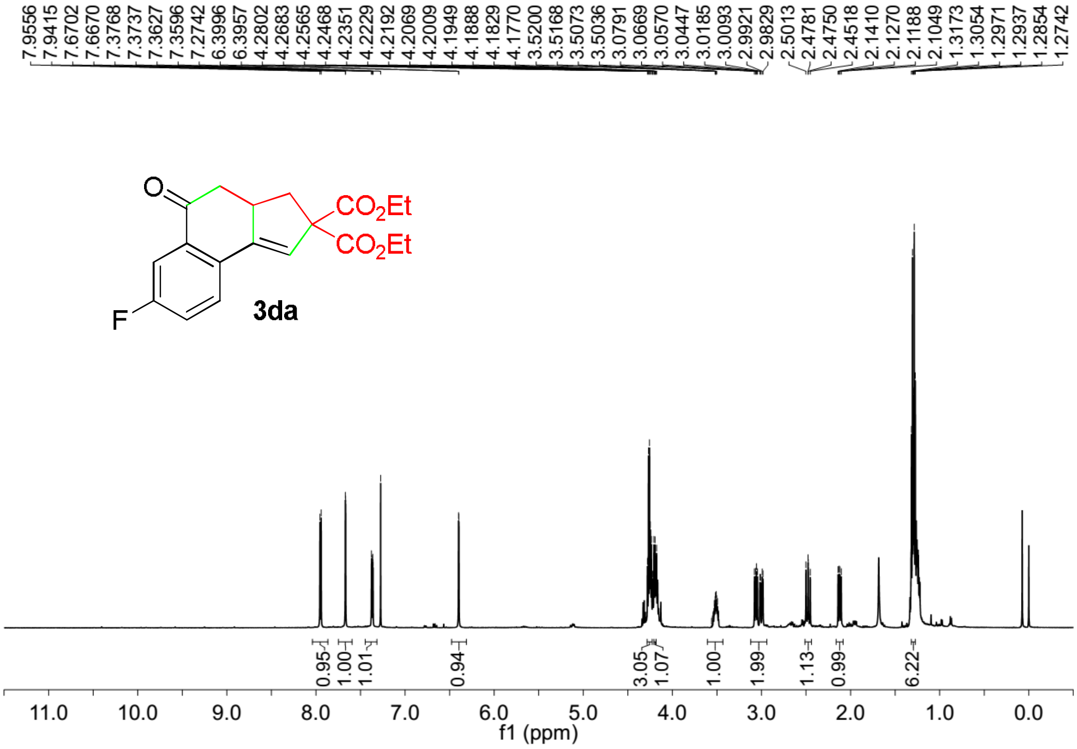
7.9556
7.9415
7.6702
7.6670
7.3768
7.3737
7.3627
7.3596
7.2742
6.3996
6.3957
4.2802
4.2683
4.2565
4.2468
4.2351
4.2229
4.2192
4.2069
4.2009
4.1949
4.1888
4.1829
4.1770
3.5200
3.5168
3.5073
3.5036
3.0791
3.0669
3.0570
3.0447
3.0185
3.0093
2.9921
2.9829
2.5013
2.4781
2.4750
2.4518
2.1410
2.1270
2.1188
2.1049
1.3173
1.3054
1.2971
1.2937
1.2854
1.2742
O
CO2Et
CO2Et
F
3da
0.95
1.00
1.01
0.94
3.05
1.07
1.00
1.99
1.13
0.99
6.22
11.0
10.0
9.0
8.0
7.0
6.0
5.0
4.0
3.0
2.0
1.0
0.0
f1 (ppm)

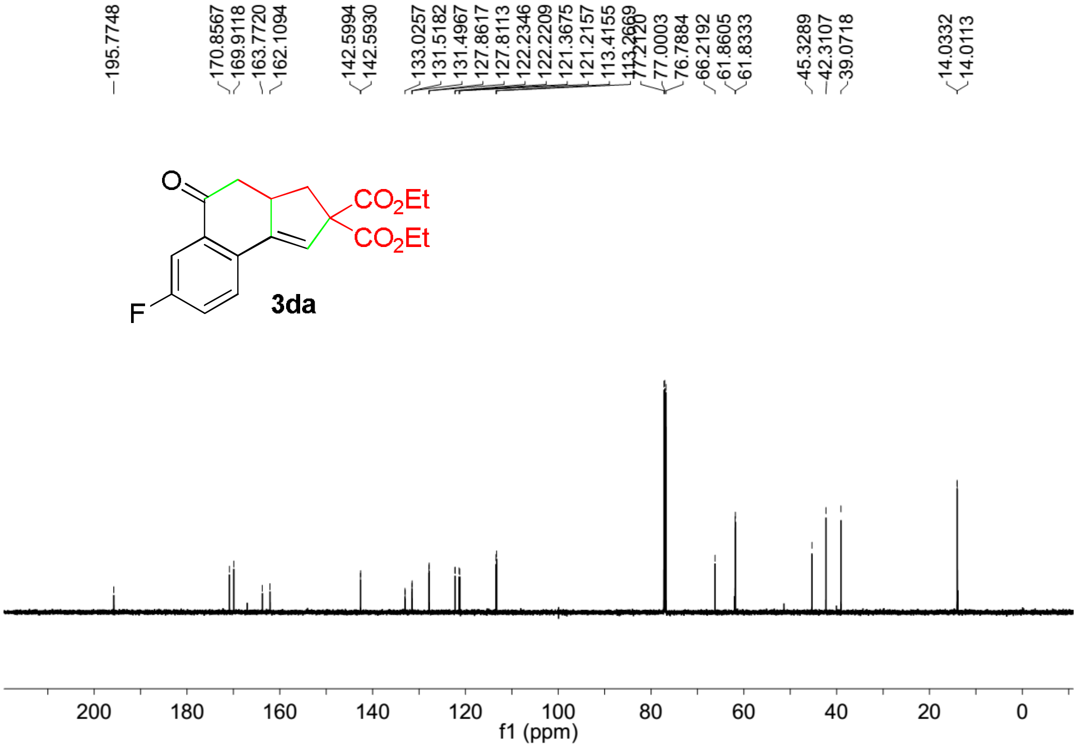
195.7748
170.8567
169.9118
163.7720
162.1094
142.5994
142.5930
133.0257
131.5182
131.4967
127.8617
127.8113
122.2346
122.2209
121.3675
121.2157
113.4155
113.2669
77.2120
77.0003
76.7884
66.2192
61.8605
61.8333
45.3289
42.3107
39.0718
14.0332
14.0113
O
CO2Et
CO2Et
F
3da
200
180
160
140
120
100
80
60
40
20
0
f1 (ppm)

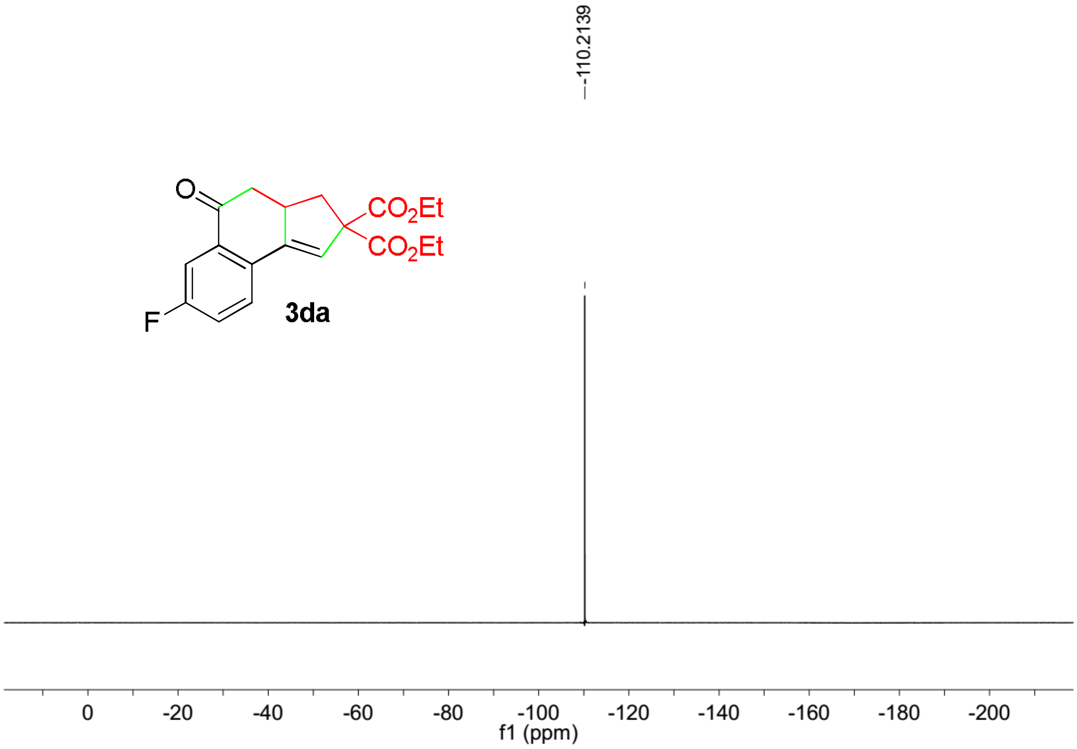
-110.2139
CO2Et
CO2Et
O
F
3da
0
-20
-40
-60
-80
-100
-120
-140
-160
-180
-200
f1 (ppm)

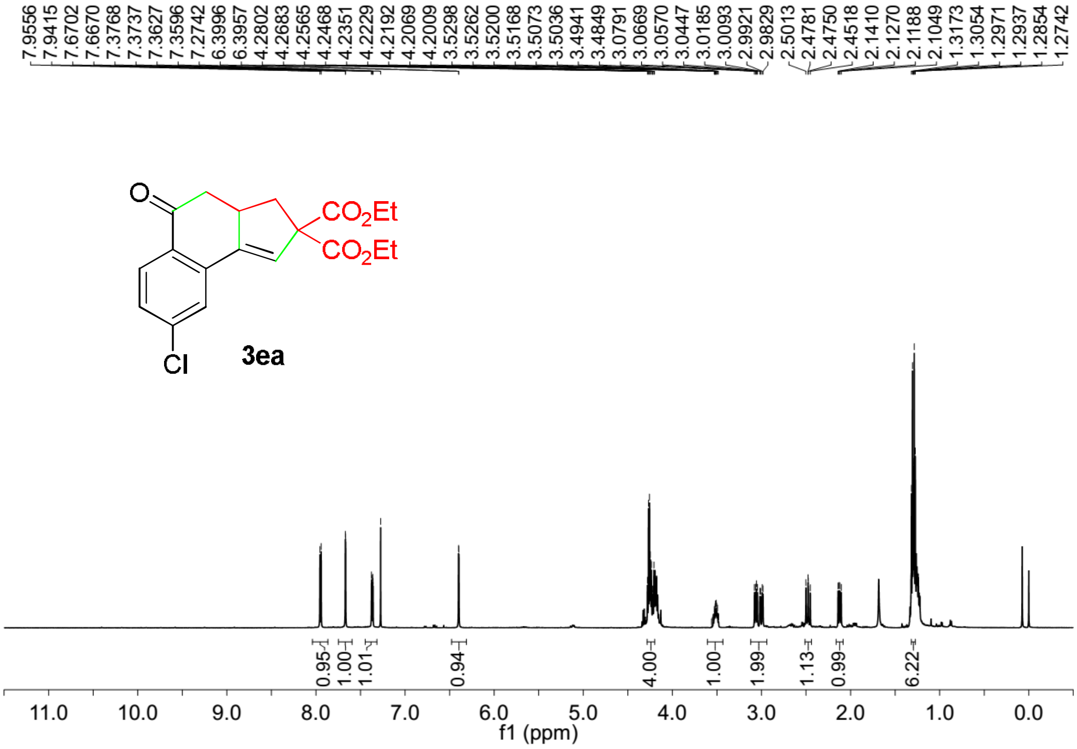
7.9556 7.9415 7.6702 7.6670 7.3768 7.3737 7.3627 7.3596 7.2742 6.3996 6.3957 4.2802 4.2683 4.2565 4.2468 4.2351 4.2229 4.2192 4.2069 4.2009 3.5298 3.5262 3.5200 3.5168 3.5073 3.5036 3.4941 3.4849 3.0791 3.0669 3.0570 3.0447 3.0185 3.0093 2.9921 2.9829 2.5013 2.4781 2.4750 2.4518 2.1410 2.1270 2.1188 2.1049 1.3173 1.3054 1.2971 1.2937 1.2854 1.2742
O
CO2Et
CO2Et
Cl
3ea
0.95
1.00
1.01
0.94
4.00
1.00
1.99
1.13
0.99
6.22
11.0 10.0 9.0 8.0 7.0 6.0 5.0 4.0 3.0 2.0 1.0 0.0
f1 (ppm)

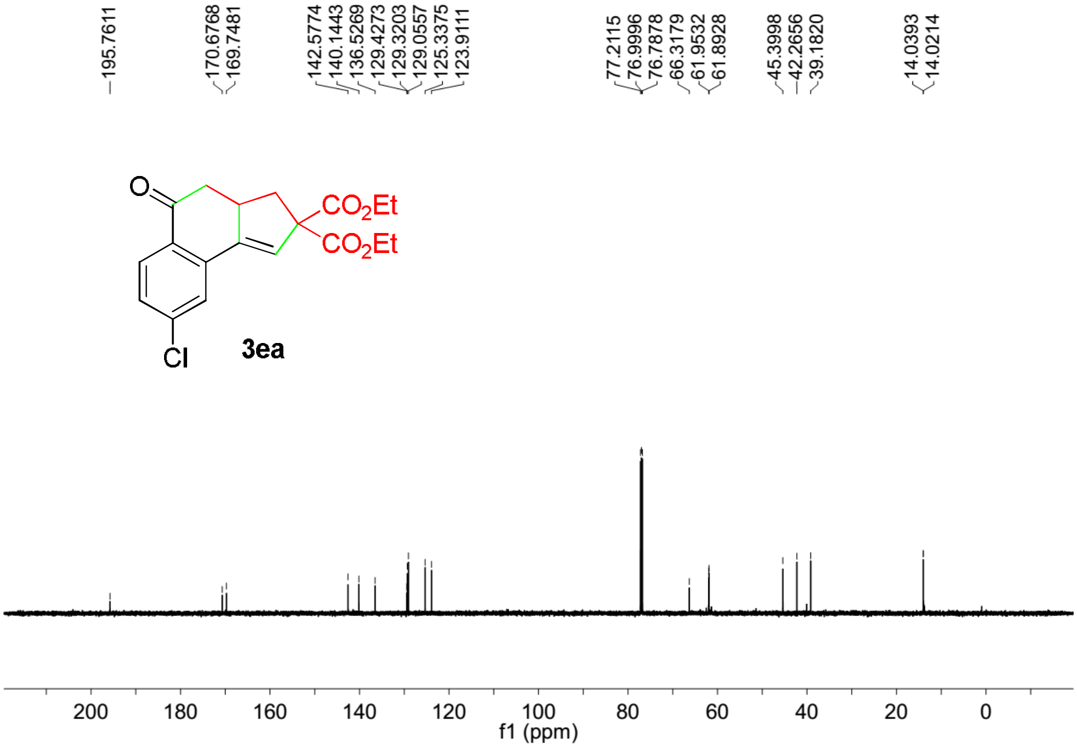
195.7611
170.6768 169.7481
142.5774 140.1443 136.5269 129.4273 129.3203 129.0557 125.3375 123.9111
77.2115 76.9996 76.7878 66.3179 61.9532 61.8928
45.3998 42.2656 39.1820
14.0393 14.0214
O
CO2Et
CO2Et
Cl
3ea
200 180 160 140 120 100 80 60 40 20 0
f1 (ppm)

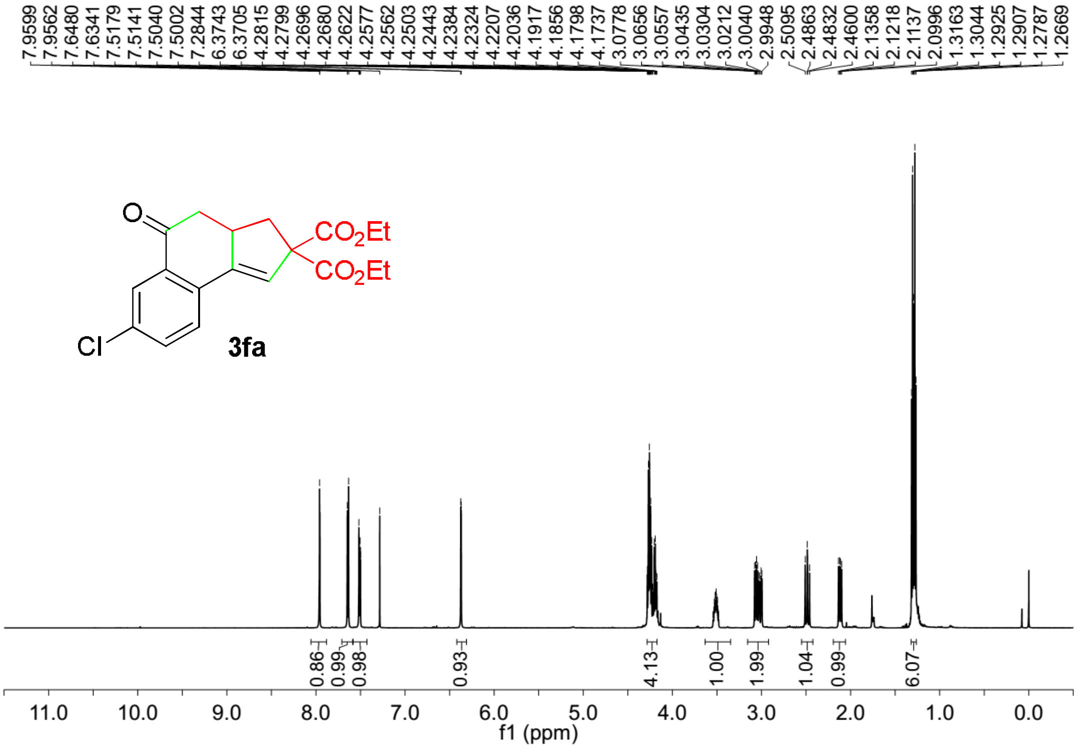
7.9599
7.9562
7.6480
7.6341
7.5179
7.5141
7.5040
7.5002
7.2844
6.3743
6.3705
4.2815
4.2799
4.2696
4.2680
4.2622
4.2577
4.2562
4.2503
4.2443
4.2384
4.2324
4.2207
4.2036
4.1917
4.1856
4.1798
4.1737
3.0778
3.0656
3.0557
3.0435
3.0304
3.0212
3.0040
2.9948
2.5095
2.4863
2.4832
2.4600
2.1358
2.1218
2.1137
2.0996
1.3163
1.3044
1.2925
1.2907
1.2787
1.2669
O
CO_2Et
CO_2Et
Cl
3fa
0.86
0.99
0.98
0.93
4.13
1.00
1.99
1.04
0.99
6.07
11.0
10.0
9.0
8.0
7.0
6.0
5.0
4.0
3.0
2.0
1.0
0.0
f1 (ppm)

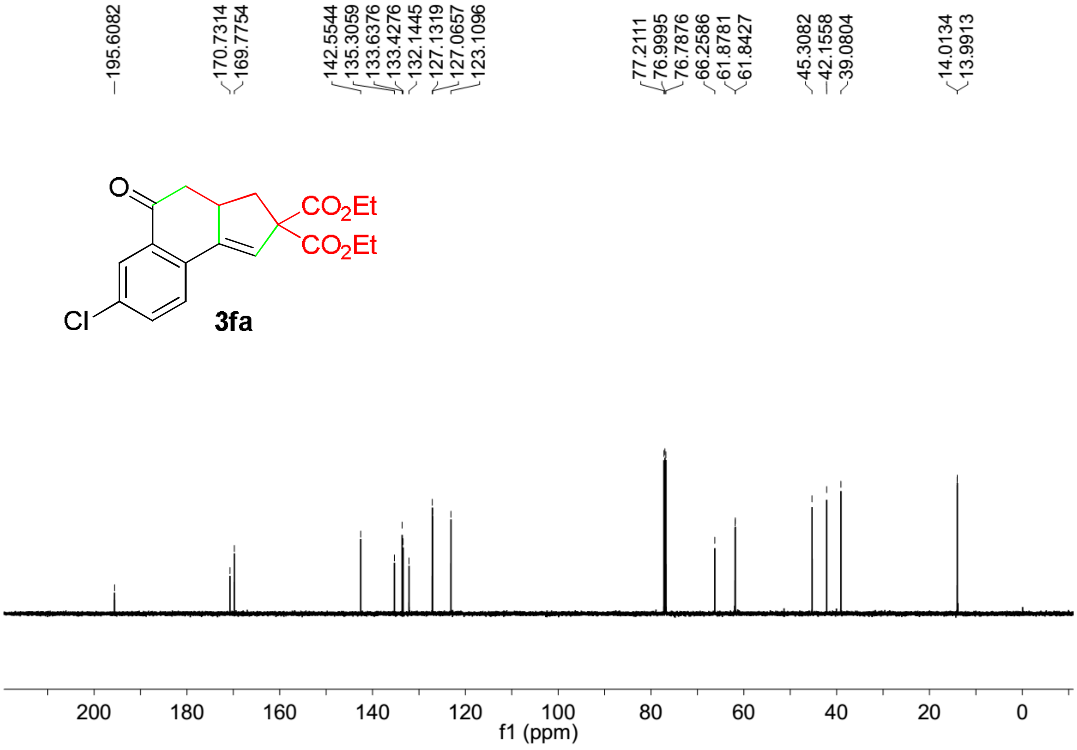
195.6082
170.7314
169.7754
142.5544
135.3059
133.6376
133.4276
132.1445
127.1319
127.0657
123.1096
77.2111
76.9995
76.7876
66.2586
61.8781
61.8427
45.3082
42.1558
39.0804
14.0134
13.9913
O
CO_2Et
CO_2Et
Cl
3fa
200
180
160
140
120
100
80
60
40
20
0
f1 (ppm)

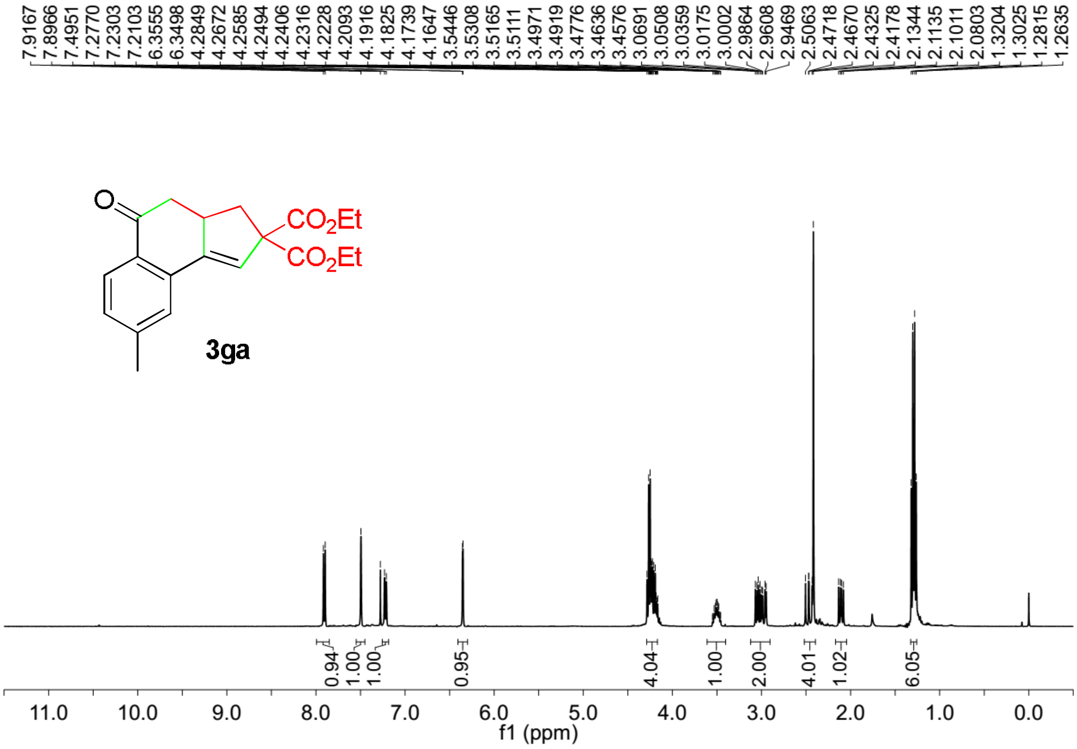

7.9167
7.8966
7.4951
7.2770
7.2303
7.2103
6.3555
6.3498
4.2849
4.2672
4.2585
4.2494
4.2406
4.2316
4.2228
4.2093
4.1916
4.1825
4.1739
4.1647
3.5446
3.5308
3.5165
3.5111
3.4971
3.4919
3.4776
3.4636
3.4576
3.0691
3.0508
3.0359
3.0175
3.0002
2.9864
2.9608
2.9469
2.5063
2.4718
2.4670
2.4325
2.4178
2.1344
2.1135
2.1011
2.0803
1.3204
1.3025
1.2815
1.2635
CO2Et
CO2Et
3ga
0.94
1.00
1.00
0.95
4.04
1.00
2.00
4.01
1.02
6.05
11.0
10.0
9.0
8.0
7.0
6.0
5.0
4.0
3.0
2.0
1.0
0.0
f1 (ppm)

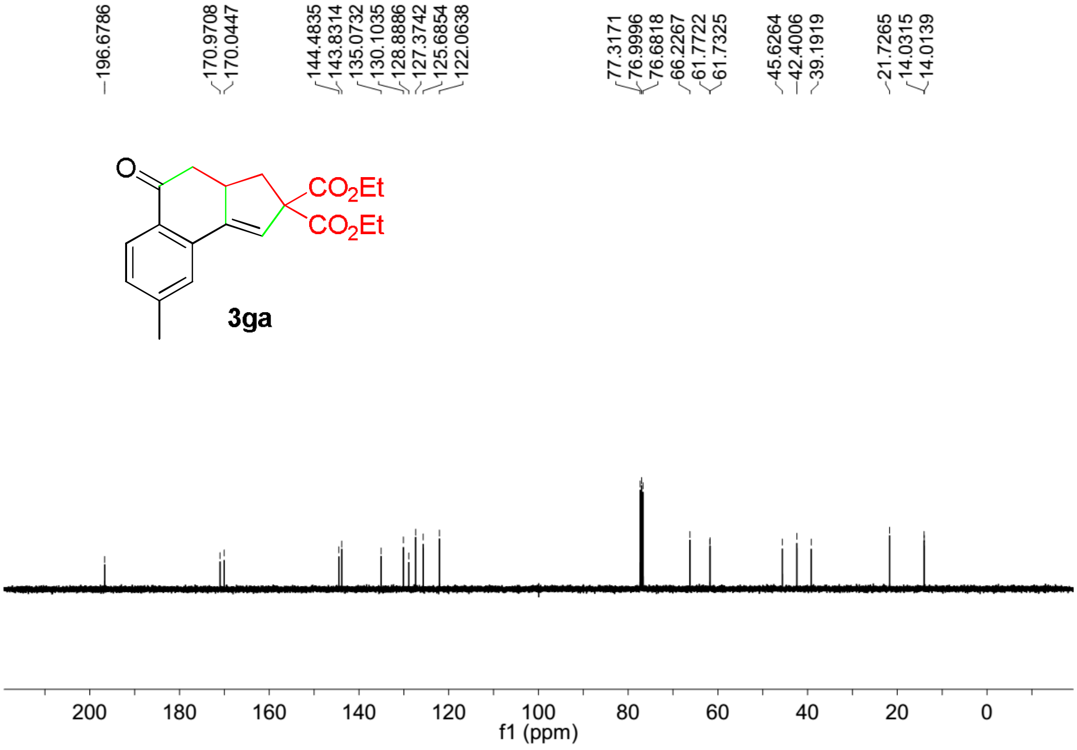

196.6786
170.9708
170.0447
144.4835
143.8314
135.0732
130.1035
128.8886
127.3742
125.6854
122.0638
77.3171
76.9996
76.6818
66.2267
61.7722
61.7325
45.6264
42.4006
39.1919
21.7265
14.0315
14.0139
CO2Et
CO2Et
3ga
200
180
160
140
120
100
80
60
40
20
0
f1 (ppm)

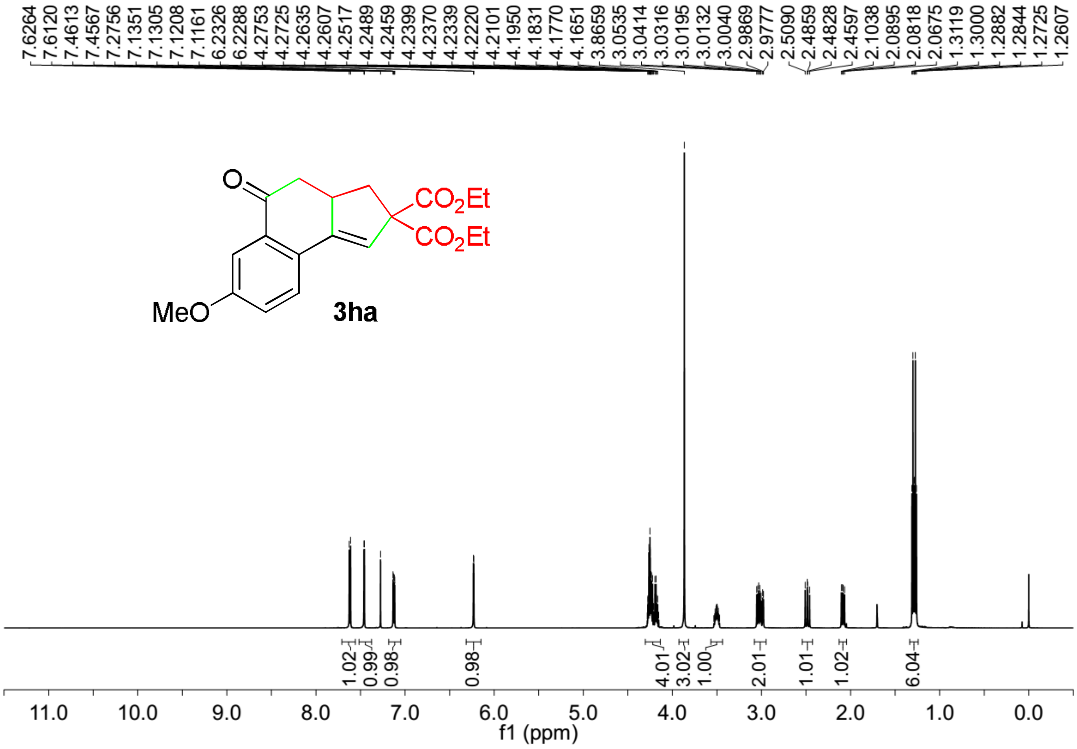
7.6264
7.6120
7.4613
7.4567
7.2756
7.1351
7.1305
7.1208
7.1161
6.2326
6.2288
4.2753
4.2725
4.2635
4.2607
4.2517
4.2489
4.2459
4.2399
4.2370
4.2339
4.2220
4.2101
4.1950
4.1831
4.1770
4.1651
3.8659
3.0535
3.0414
3.0316
3.0195
3.0132
3.0040
2.9869
2.9777
2.5090
2.4859
2.4828
2.4597
2.1038
2.0895
2.0818
2.0675
1.3119
1.3000
1.2882
1.2844
1.2725
1.2607
O
CO_2Et
CO_2Et
MeO
3ha
1.02
0.99
0.98
0.98
4.01
3.02
1.00
2.01
1.01
1.02
6.04
11.0
10.0
9.0
8.0
7.0
6.0
5.0
4.0
3.0
2.0
1.0
0.0
f1 (ppm)

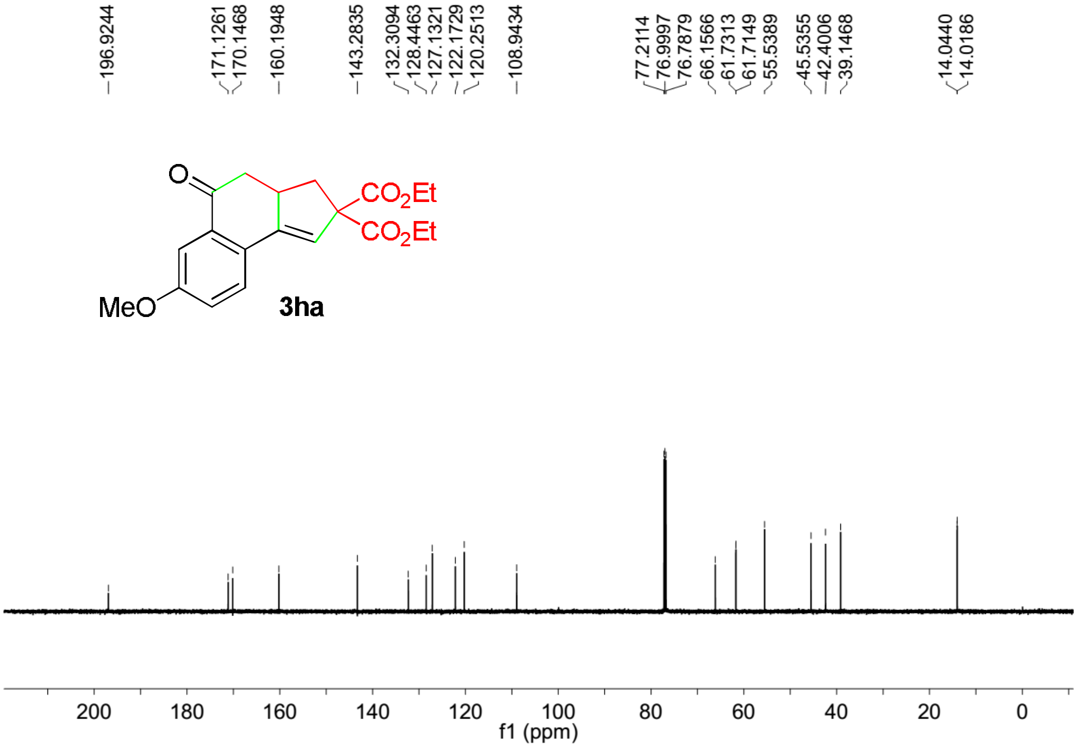
196.9244
171.1261
170.1468
160.1948
143.2835
132.3094
128.4463
127.1321
122.1729
120.2513
108.9434
77.2114
76.9997
76.7879
66.1566
61.7313
61.7149
55.5389
45.5355
42.4006
39.1468
14.0440
14.0186
O
CO_2Et
CO_2Et
MeO
3ha
200
180
160
140
120
100
80
60
40
20
0
f1 (ppm)

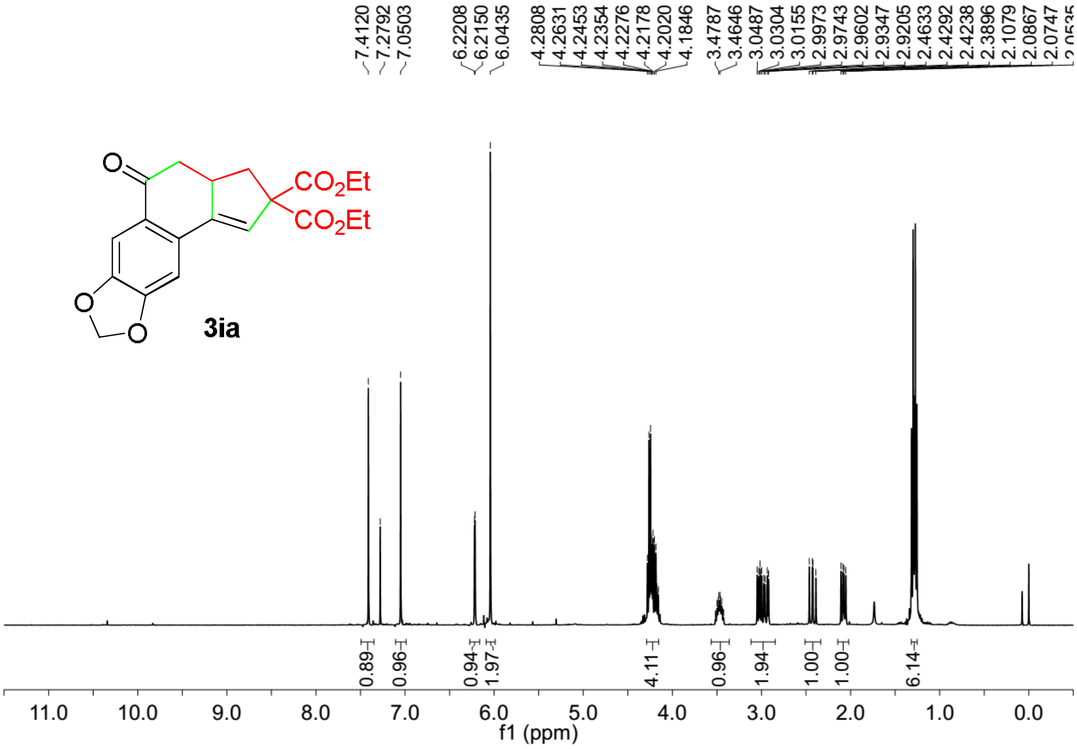

7.4120
7.2792
7.0503
6.2208
6.2150
6.0435
4.2808
4.2631
4.2453
4.2354
4.2276
4.2178
4.2020
4.1846
3.4787
3.4646
3.0487
3.0304
3.0155
2.9973
2.9743
2.9602
2.9347
2.9205
2.4633
2.4292
2.4238
2.3896
2.1079
2.0867
2.0747
CO_2Et
CO_2Et
3ia
0.89
0.96
0.94
1.97
4.11
0.96
1.94
1.00
1.00
6.14
11.0 10.0 9.0 8.0 7.0 6.0 5.0 4.0 3.0 2.0 1.0 0.0
f1 (ppm)

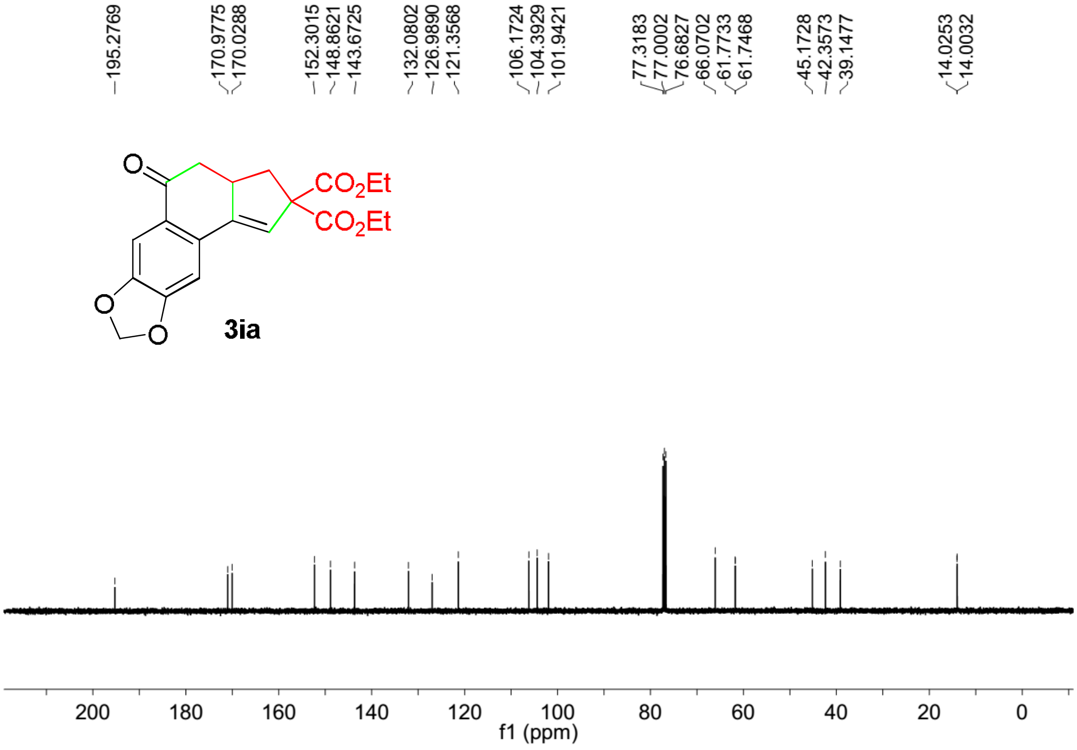

195.2769
170.9775
170.0288
152.3015
148.8621
143.6725
132.0802
126.9890
121.3568
106.1724
104.3929
101.9421
77.3183
77.0002
76.6827
66.0702
61.7733
61.7468
45.1728
42.3573
39.1477
14.0253
14.0032
CO_2Et
CO_2Et
3ia
200 180 160 140 120 100 80 60 40 20 0
f1 (ppm)

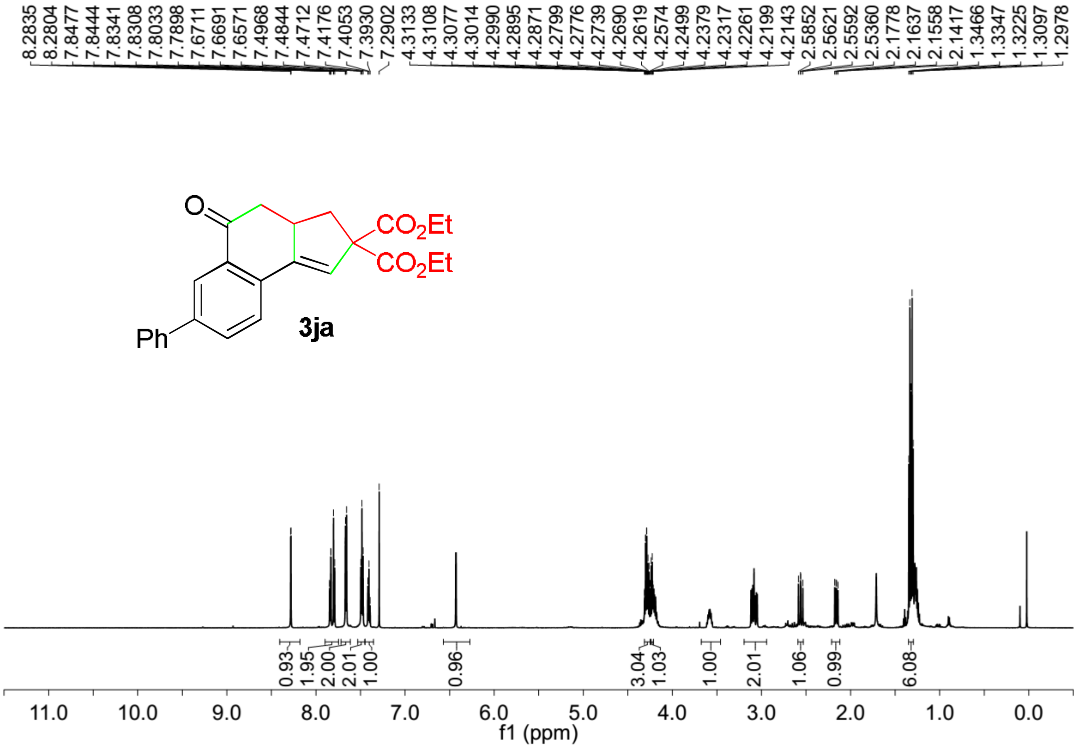
8.2835
8.2804
7.8477
7.8444
7.8341
7.8308
7.8033
7.7898
7.6711
7.6691
7.6571
7.4968
7.4844
7.4712
7.4176
7.4053
7.3930
7.2902
4.3133
4.3108
4.3077
4.3014
4.2990
4.2895
4.2871
4.2799
4.2776
4.2739
4.2690
4.2619
4.2574
4.2499
4.2379
4.2317
4.2261
4.2199
4.2143
2.5852
2.5621
2.5592
2.5360
2.1778
2.1637
2.1558
2.1417
1.3466
1.3347
1.3225
1.3097
1.2978
O
CO_2Et
CO_2Et
Ph
3ja
0.93
1.95
2.00
2.01
1.00
0.96
3.04
1.03
1.00
2.01
1.06
0.99
6.08
11.0
10.0
9.0
8.0
7.0
6.0
5.0
4.0
3.0
2.0
1.0
0.0
f1 (ppm)

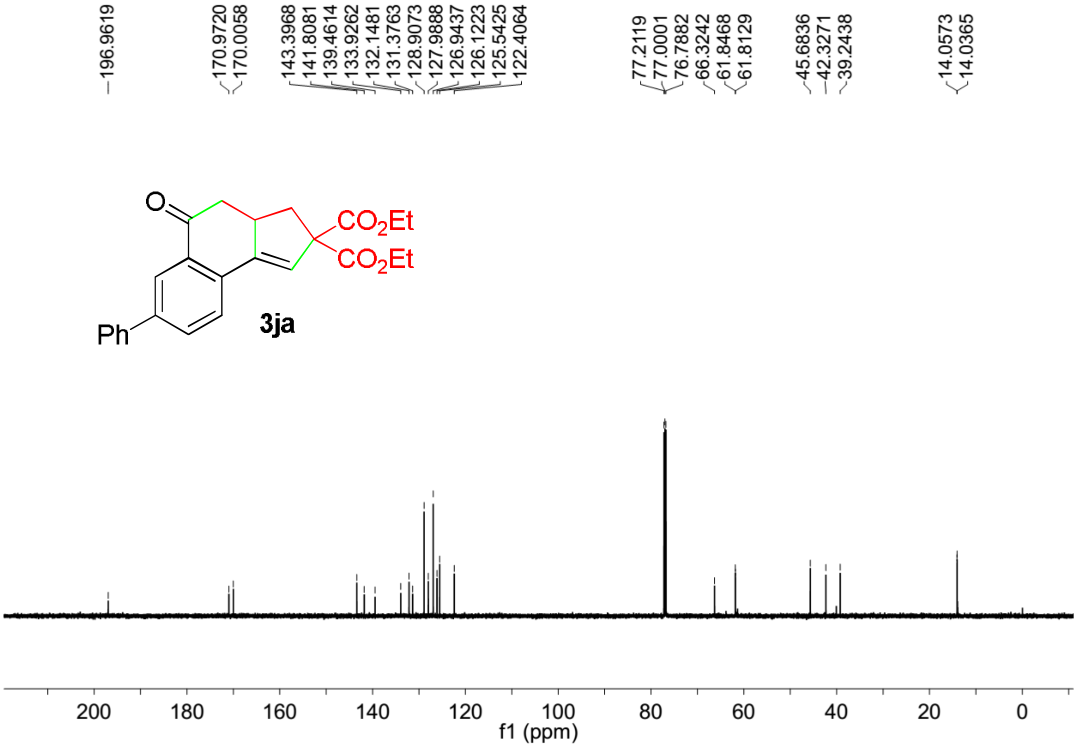
196.9619
170.9720
170.0058
143.3968
141.8081
139.4614
133.9262
132.1481
131.3763
128.9073
127.9888
126.9437
126.1223
125.5425
122.4064
77.2119
77.0001
76.7882
66.3242
61.8468
61.8129
45.6836
42.3271
39.2438
14.0573
14.0365
O
CO_2Et
CO_2Et
Ph
3ja
200
180
160
140
120
100
80
60
40
20
0
f1 (ppm)

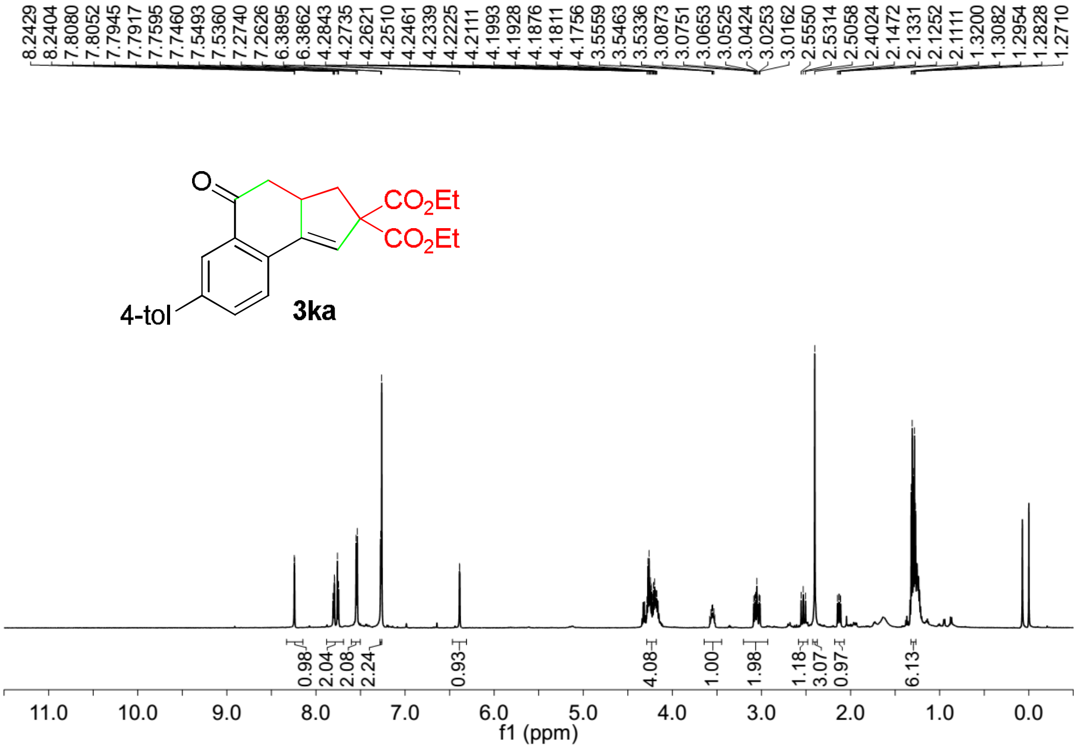

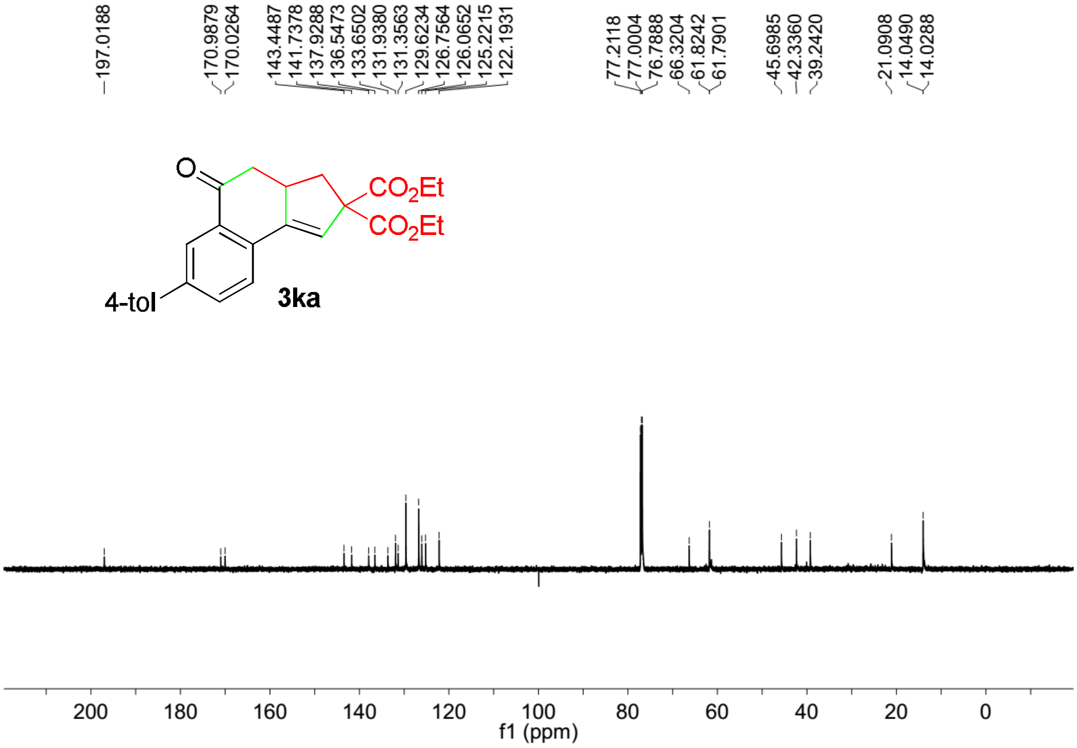

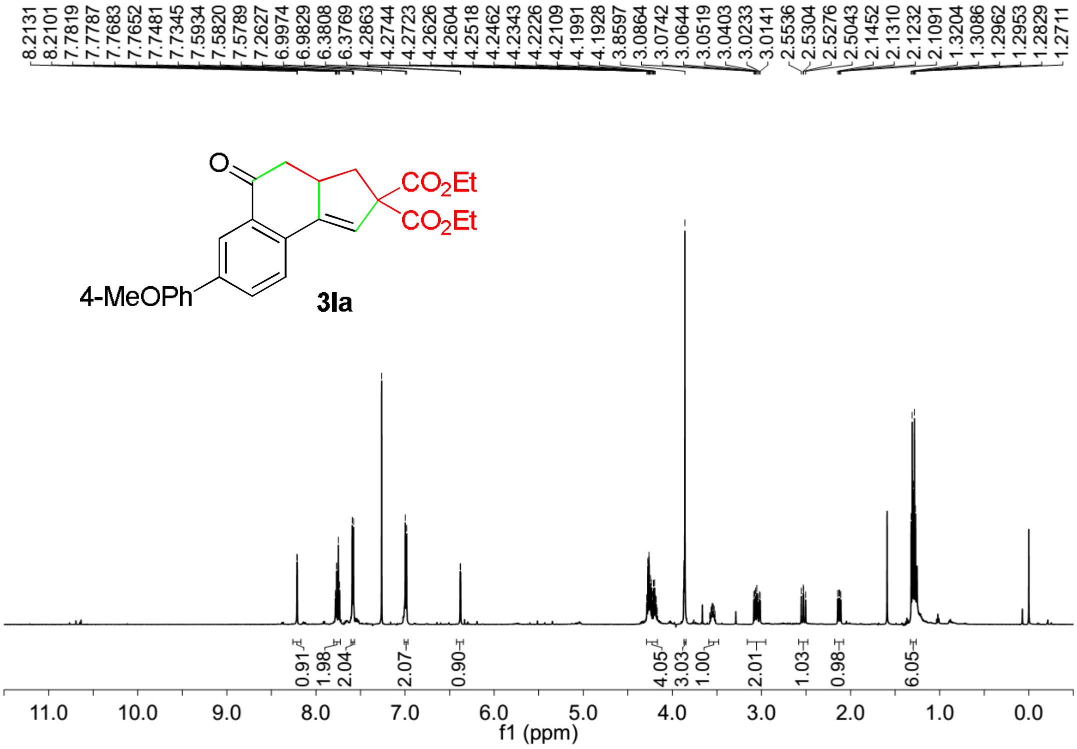

8.2131
8.2101
7.7819
7.7787
7.7683
7.7652
7.7481
7.7345
7.5934
7.5820
7.5789
7.2627
6.9974
6.9829
6.3808
6.3769
4.2863
4.2744
4.2723
4.2626
4.2604
4.2518
4.2462
4.2343
4.2226
4.2109
4.1991
4.1928
3.8597
3.0864
3.0742
3.0644
3.0519
3.0403
3.0233
3.0141
2.5536
2.5304
2.5276
2.5043
2.1452
2.1310
2.1232
2.1091
1.3204
1.3086
1.2962
1.2953
1.2829
1.2711
O
CO2Et
CO2Et
4-MeOPh
3la
0.91
1.98
2.04
2.07
0.90
4.05
3.03
1.00
2.01
1.03
0.98
6.05
11.0
10.0
9.0
8.0
7.0
6.0
5.0
4.0
3.0
2.0
1.0
0.0
f1 (ppm)

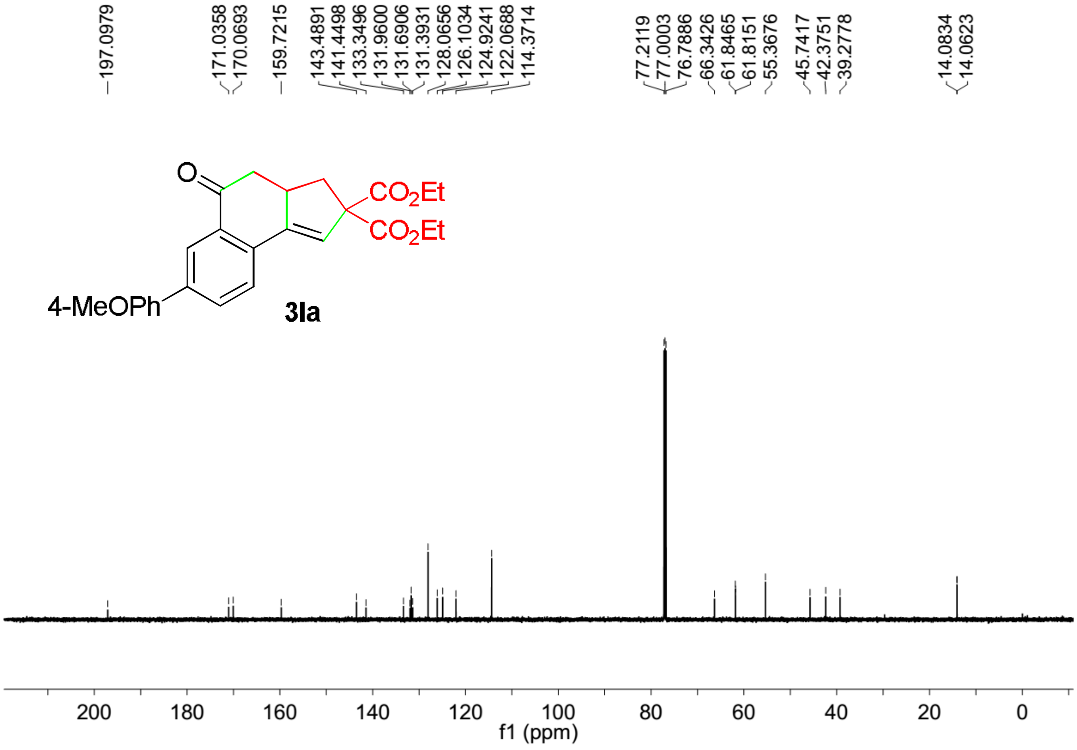

197.0979
171.0358
170.0693
159.7215
143.4891
141.4498
133.3496
131.9600
131.6906
131.3931
128.0656
126.1034
124.9241
122.0688
114.3714
77.2119
77.0003
76.7886
66.3426
61.8465
61.8151
55.3676
45.7417
42.3751
39.2778
14.0834
14.0623
O
CO2Et
CO2Et
4-MeOPh
3la
200
180
160
140
120
100
80
60
40
20
0
f1 (ppm)

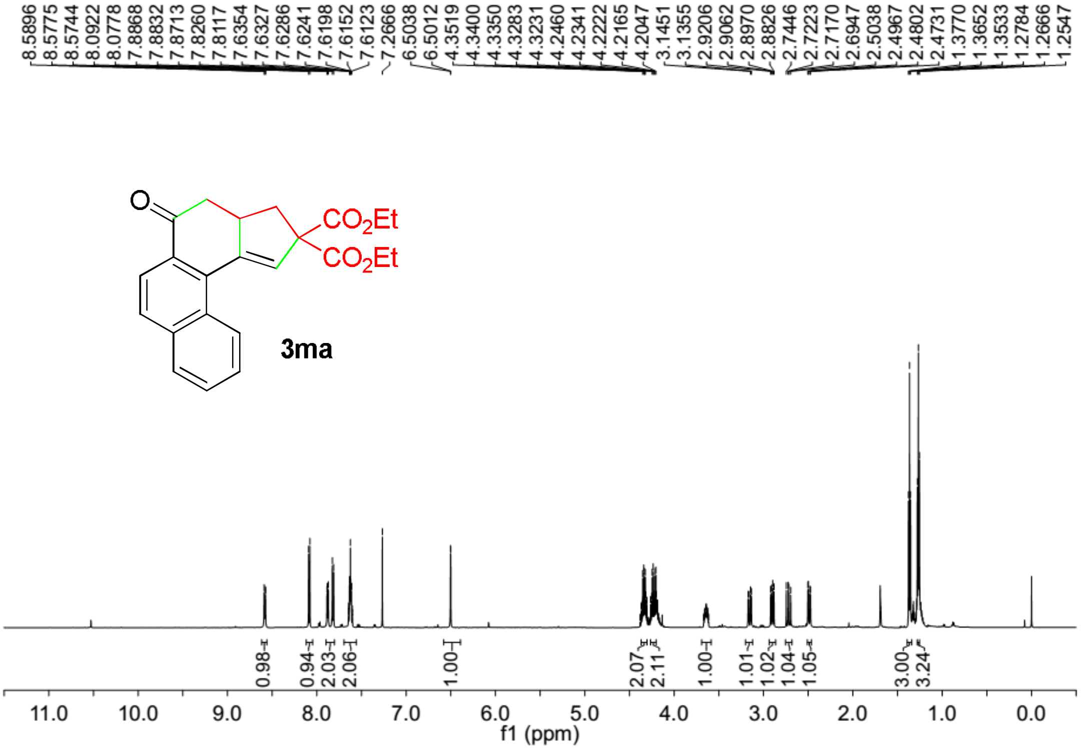
8.5896 8.5775 8.5744 8.0922 8.0778 7.8868 7.8832 7.8713 7.8260 7.8117 7.6354 7.6327 7.6286 7.6241 7.6198 7.6152 7.6123 7.2666 6.5038 6.5012 4.3519 4.3400 4.3350 4.3283 4.3231 4.2460 4.2341 4.2222 4.2165 4.2047 3.1451 3.1355 2.9206 2.9062 2.8970 2.8826 2.7446 2.7223 2.7170 2.6947 2.5038 2.4967 2.4802 2.4731 1.3770 1.3652 1.3533 1.2784 1.2666 1.2547
O
CO2Et
CO2Et
3ma
0.98 0.94 2.03 2.06 1.00 2.07 2.11 1.00 1.01 1.02 1.04 1.05 3.00 3.24
11.0 10.0 9.0 8.0 7.0 6.0 5.0 4.0 3.0 2.0 1.0 0.0
f1 (ppm)

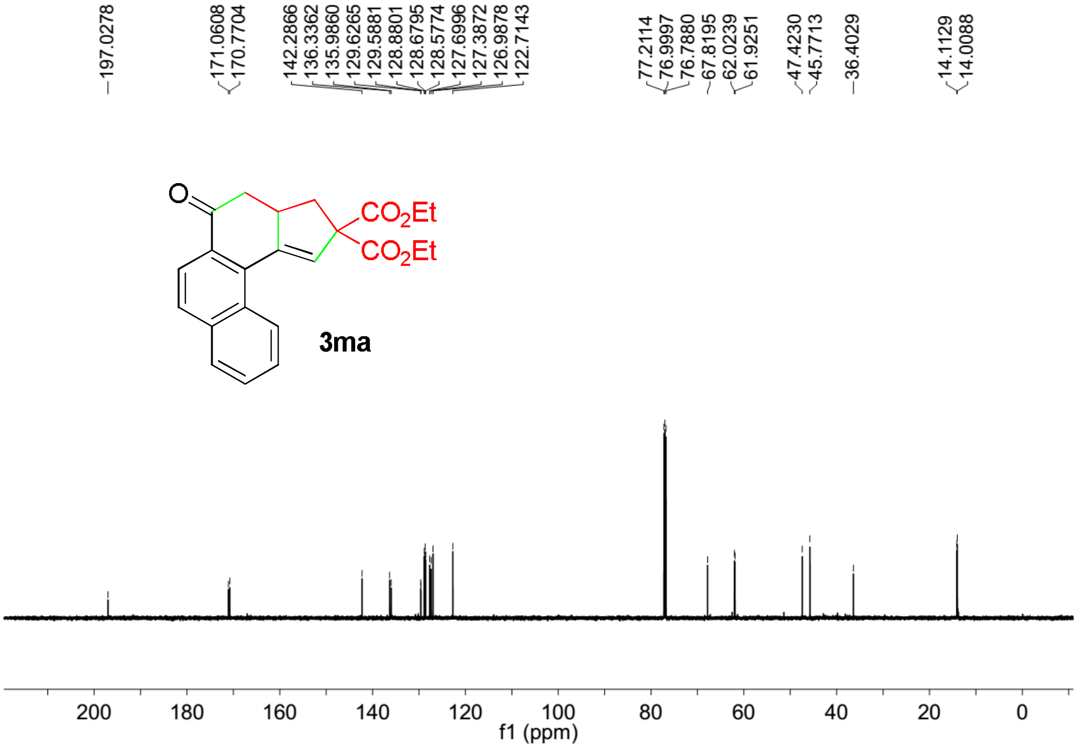
197.0278
171.0608 170.7704
142.2866 136.3362 135.9860 129.6265 129.5881 128.8801 128.6795 128.5774 127.6996 127.3872 126.9878 122.7143
77.2114 76.9997 76.7880 67.8195 62.0239 61.9251 47.4230 45.7713 36.4029 14.1129 14.0088
O
CO2Et
CO2Et
3ma
200 180 160 140 120 100 80 60 40 20 0
f1 (ppm)

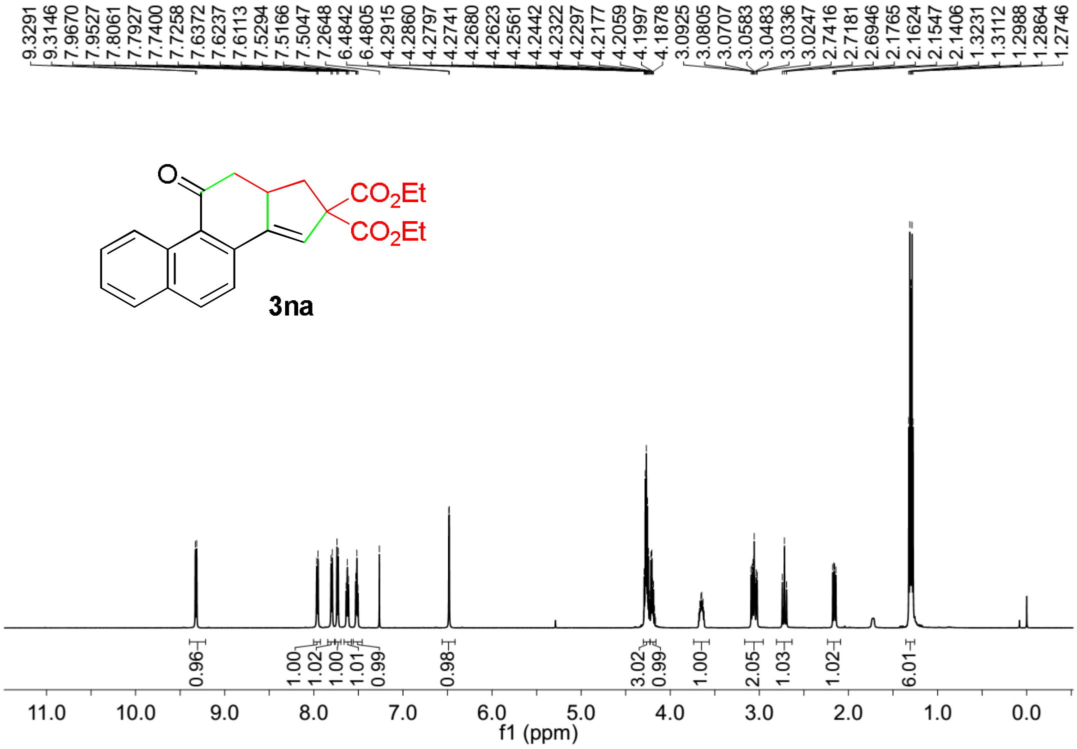
9.3291
9.3146
7.9670
7.9527
7.8061
7.7927
7.7400
7.7258
7.6372
7.6237
7.6113
7.5294
7.5166
7.5047
7.2648
6.4842
6.4805
4.2915
4.2860
4.2797
4.2741
4.2680
4.2623
4.2561
4.2442
4.2322
4.2297
4.2177
4.2059
4.1997
4.1878
3.0925
3.0805
3.0707
3.0583
3.0483
3.0336
3.0247
2.7416
2.7181
2.6946
2.1765
2.1624
2.1547
2.1406
1.3231
1.3112
1.2988
1.2864
1.2746
CO_2Et
CO_2Et
3na
0.96
1.00
1.02
1.00
1.01
0.99
0.98
3.02
0.99
1.00
2.05
1.03
1.02
6.01
11.0
10.0
9.0
8.0
7.0
6.0
5.0
4.0
3.0
2.0
1.0
0.0
f1 (ppm)

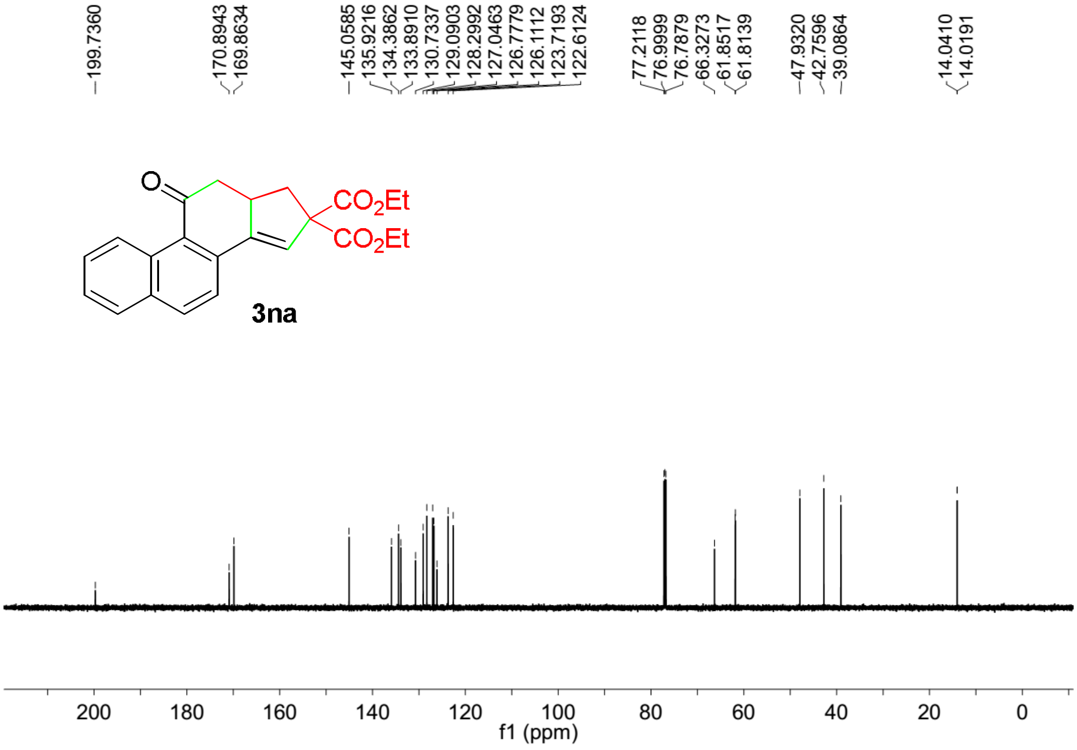
199.7360
170.8943
169.8634
145.0585
135.9216
134.3862
133.8910
130.7337
129.0903
128.2992
127.0463
126.7779
126.1112
123.7193
122.6124
77.2118
76.9999
76.7879
66.3273
61.8517
61.8139
47.9320
42.7596
39.0864
14.0410
14.0191
CO_2Et
CO_2Et
3na
200
180
160
140
120
100
80
60
40
20
0
f1 (ppm)

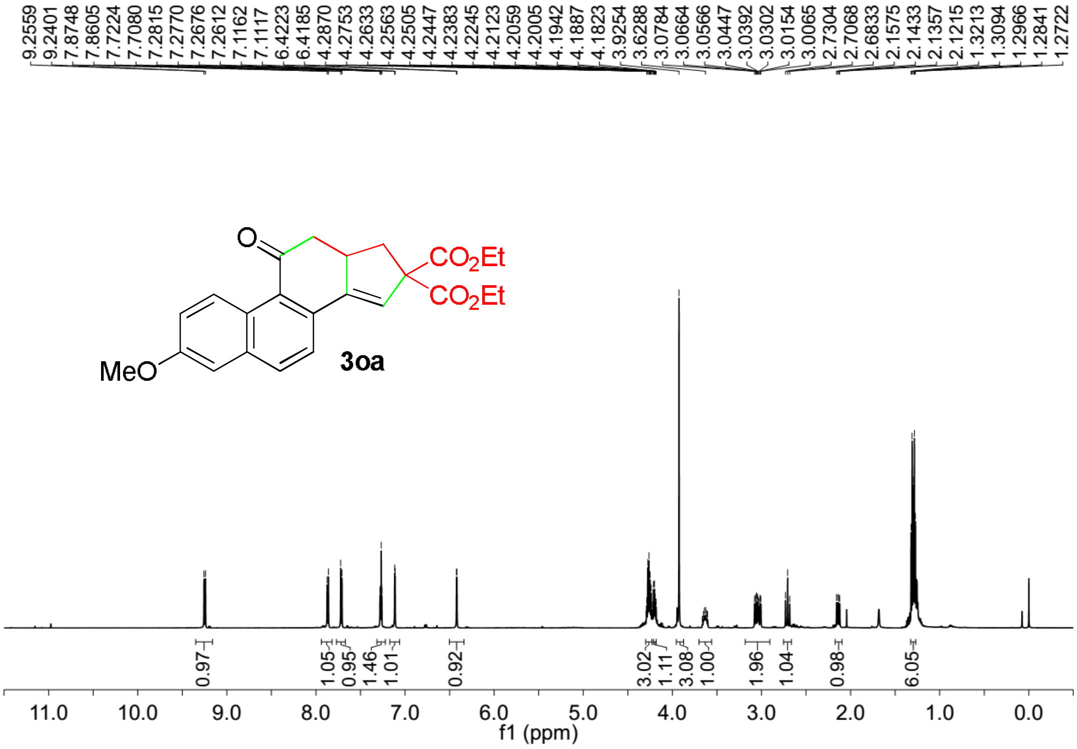
9.2559
9.2401
7.8748
7.8605
7.7224
7.7080
7.2815
7.2770
7.2676
7.2612
7.1162
7.1117
6.4223
6.4185
4.2870
4.2753
4.2633
4.2563
4.2505
4.2447
4.2383
4.2245
4.2123
4.2059
4.2005
4.1942
4.1887
4.1823
3.9254
3.6288
3.0784
3.0664
3.0566
3.0447
3.0392
3.0302
3.0154
3.0065
2.7304
2.7068
2.6833
2.1575
2.1433
2.1357
2.1215
1.3213
1.3094
1.2966
1.2841
1.2722
O
CO2Et
CO2Et
MeO
3oa
0.97
1.05
0.95
1.46
1.01
0.92
3.02
1.11
3.08
1.00
1.96
1.04
0.98
6.05
11.0
10.0
9.0
8.0
7.0
6.0
5.0
4.0
3.0
2.0
1.0
0.0
f1 (ppm)

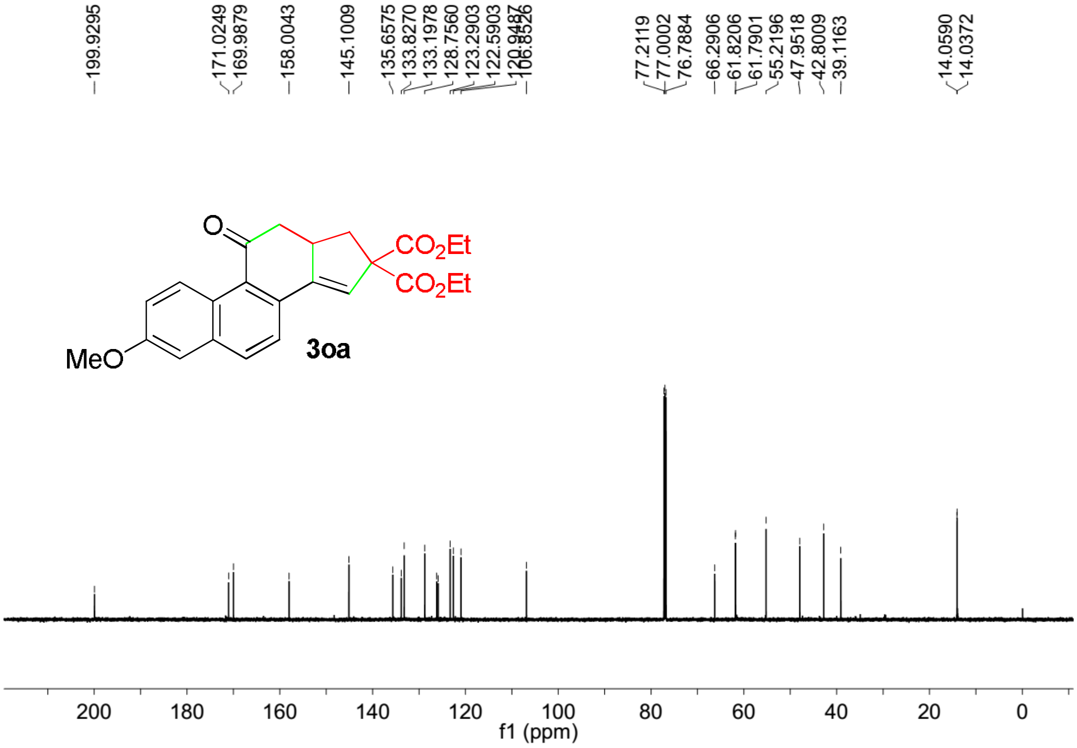
199.9295
171.0249
169.9879
158.0043
145.1009
135.6575
133.8270
133.1978
128.7560
123.2903
122.5903
120.9487
106.8526
77.2119
77.0002
76.7884
66.2906
61.8206
61.7901
55.2196
47.9518
42.8009
39.1163
14.0590
14.0372
O
CO2Et
CO2Et
MeO
3oa
200
180
160
140
120
100
80
60
40
20
0
f1 (ppm)

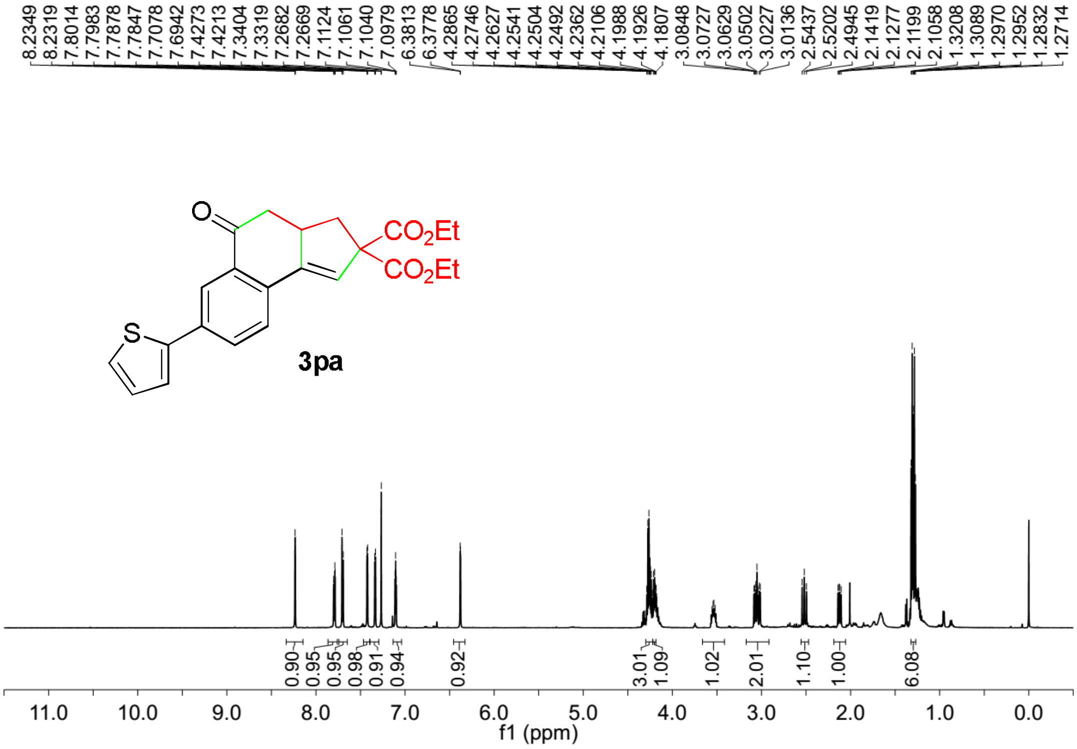
3pa
CO2Et
CO2Et
f1 (ppm)

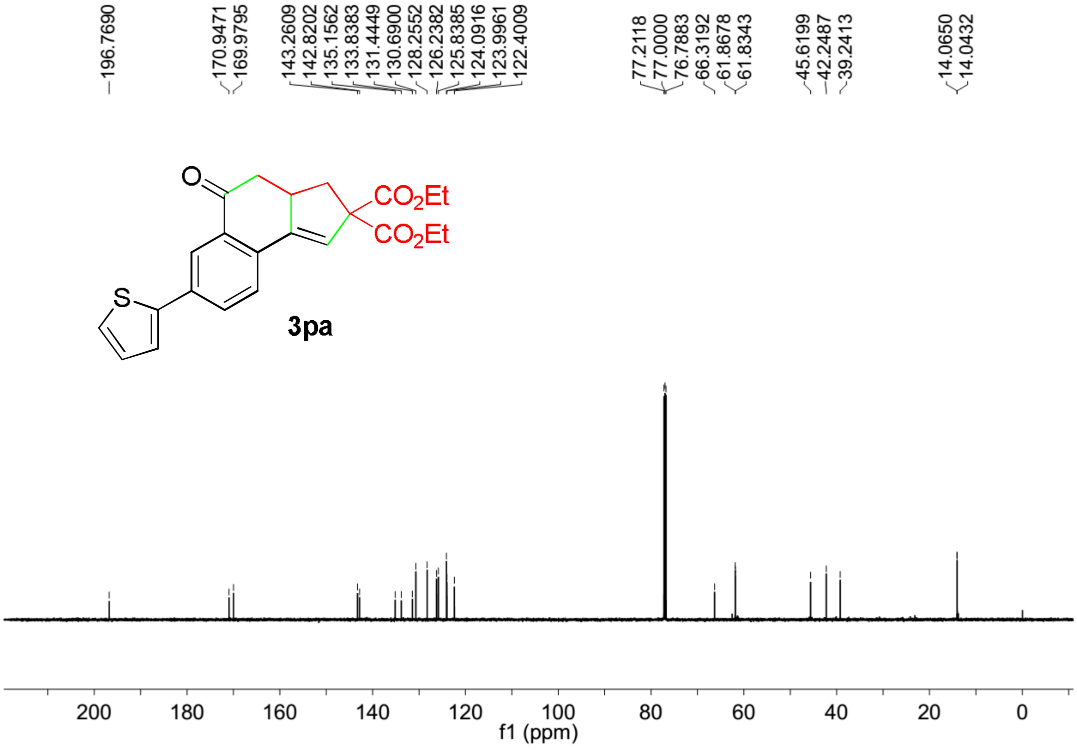
3pa
CO2Et
CO2Et
f1 (ppm)

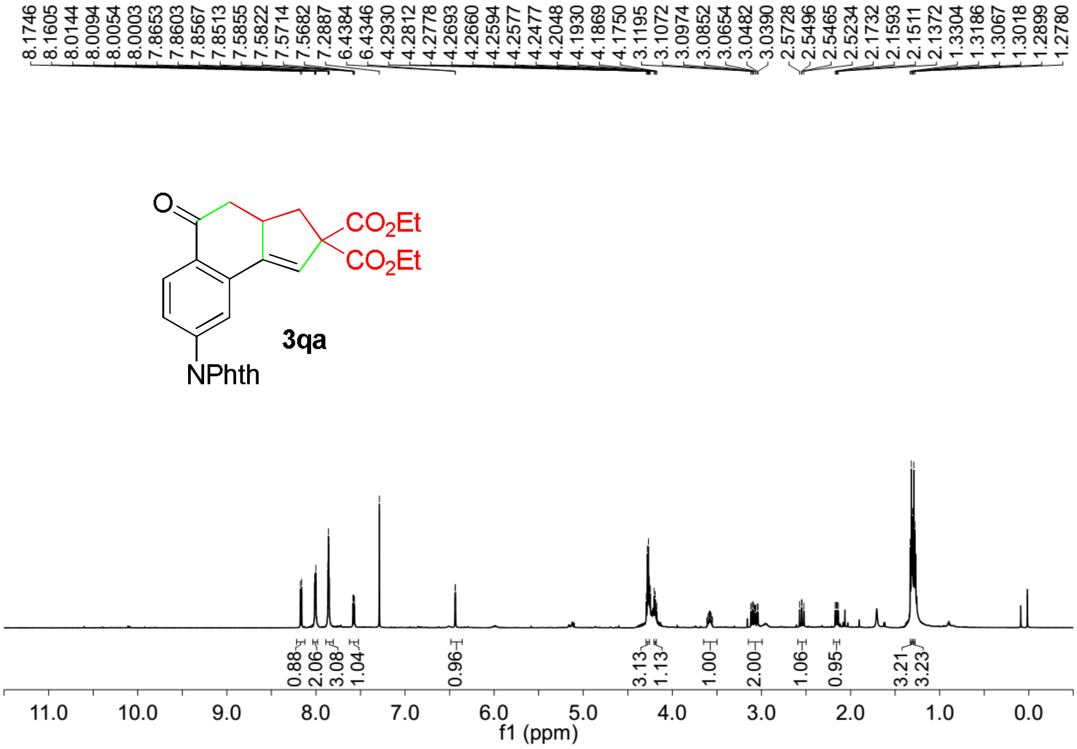
8.1746
8.1605
8.0144
8.0094
8.0054
8.0003
7.8653
7.8603
7.8567
7.8513
7.5855
7.5822
7.5714
7.5682
7.2887
6.4384
6.4346
4.2930
4.2812
4.2778
4.2693
4.2660
4.2594
4.2577
4.2477
4.2048
4.1930
4.1869
4.1750
3.1195
3.1072
3.0974
3.0852
3.0654
3.0482
3.0390
2.5728
2.5496
2.5465
2.5234
2.1732
2.1593
2.1511
2.1372
1.3304
1.3186
1.3067
1.3018
1.2899
1.2780
O
CO2Et
CO2Et
3qa
NPhth
0.88
2.06
3.08
1.04
0.96
3.13
1.13
1.00
2.00
1.06
0.95
3.21
3.23
11.0
10.0
9.0
8.0
7.0
6.0
5.0
4.0
3.0
2.0
1.0
0.0
f1 (ppm)

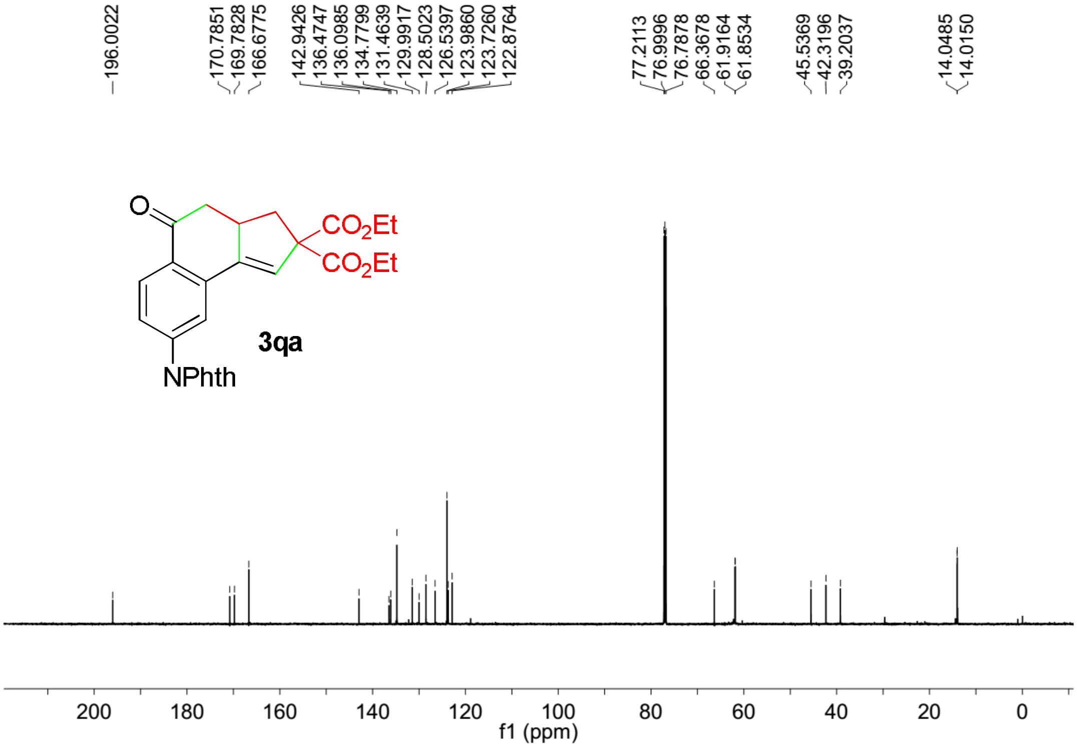
196.0022
170.7851
169.7828
166.6775
142.9426
136.4747
136.0985
134.7799
131.4639
129.9917
128.5023
126.5397
123.9860
123.7260
122.8764
77.2113
76.9996
76.7878
66.3678
61.9164
61.8534
45.5369
42.3196
39.2037
14.0485
14.0150
O
CO2Et
CO2Et
3qa
NPhth
200
180
160
140
120
100
80
60
40
20
0
f1 (ppm)

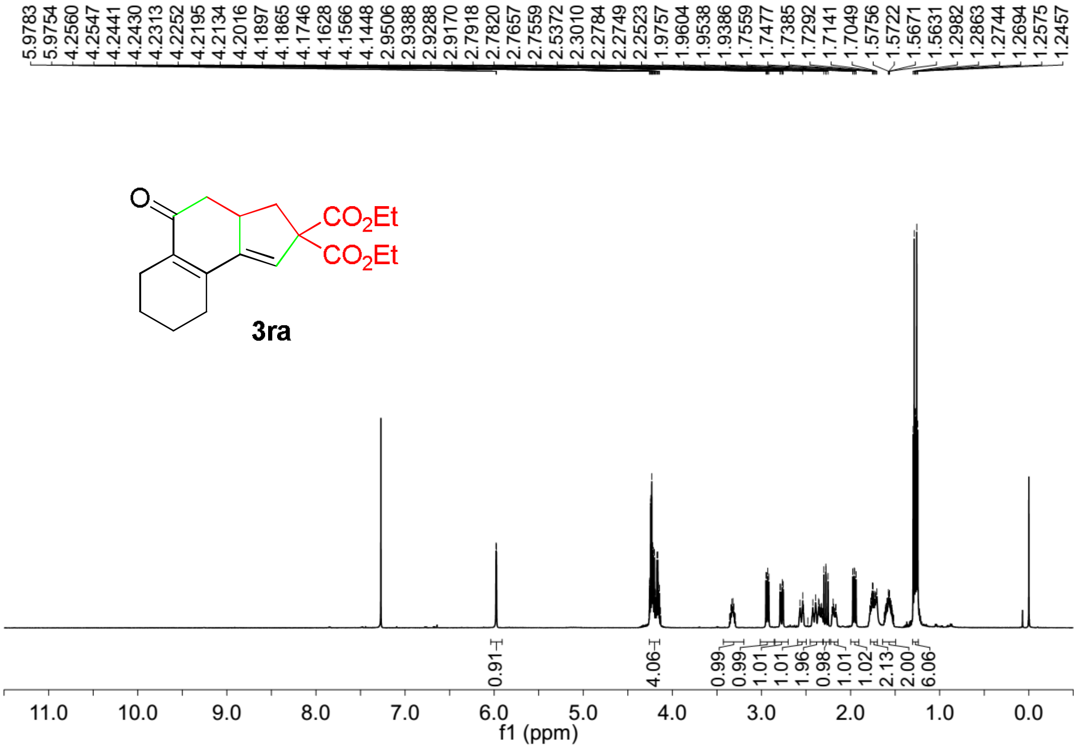

5.9783
5.9754
4.2560
4.2547
4.2441
4.2430
4.2313
4.2252
4.2195
4.2134
4.2016
4.1897
4.1865
4.1746
4.1628
4.1566
4.1448
2.9506
2.9388
2.9288
2.9170
2.7918
2.7820
2.7657
2.7559
2.5372
2.3010
2.2784
2.2749
2.2523
1.9757
1.9604
1.9538
1.9386
1.7559
1.7477
1.7385
1.7292
1.7141
1.7049
1.5756
1.5722
1.5671
1.5631
1.2982
1.2863
1.2744
1.2694
1.2575
1.2457
O
CO_2Et
CO_2Et
3ra
0.91
4.06
0.99
0.99
1.01
1.01
1.96
0.98
1.01
1.02
2.13
2.00
6.06
11.0
10.0
9.0
8.0
7.0
6.0
5.0
4.0
3.0
2.0
1.0
0.0
f1 (ppm)

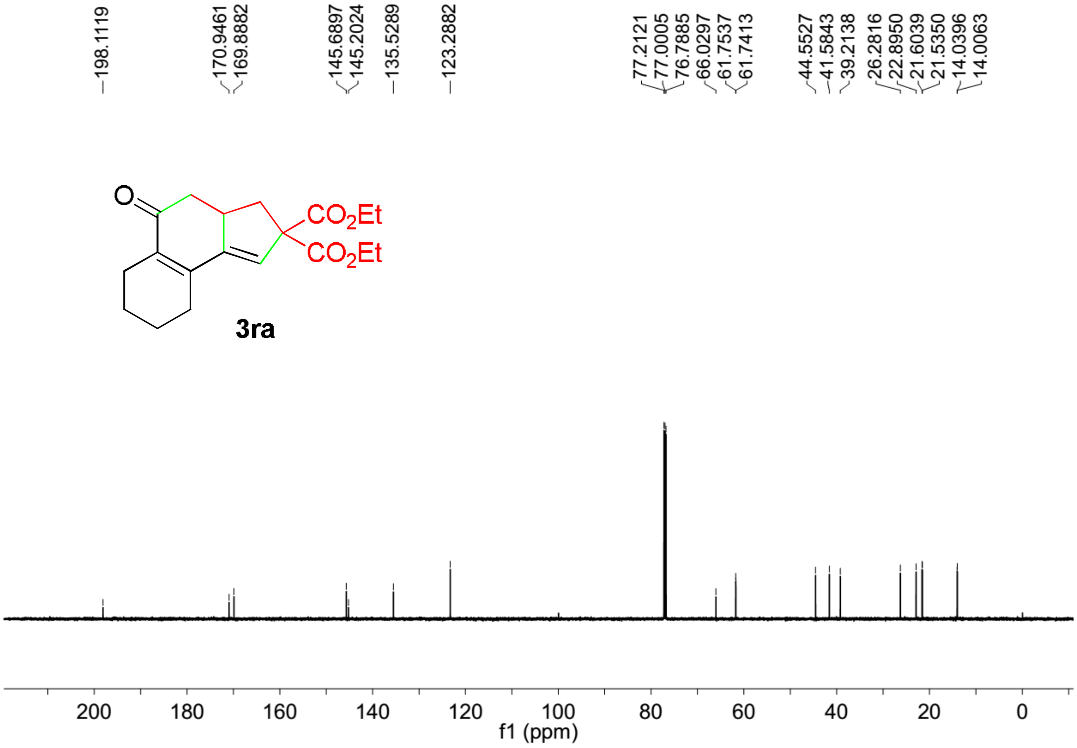

198.1119
170.9461
169.8882
145.6897
145.2024
135.5289
123.2882
77.2121
77.0005
76.7885
66.0297
61.7537
61.7413
44.5527
41.5843
39.2138
26.2816
22.8950
21.6039
21.5350
14.0396
14.0063
O
CO_2Et
CO_2Et
3ra
200
180
160
140
120
100
80
60
40
20
0
f1 (ppm)

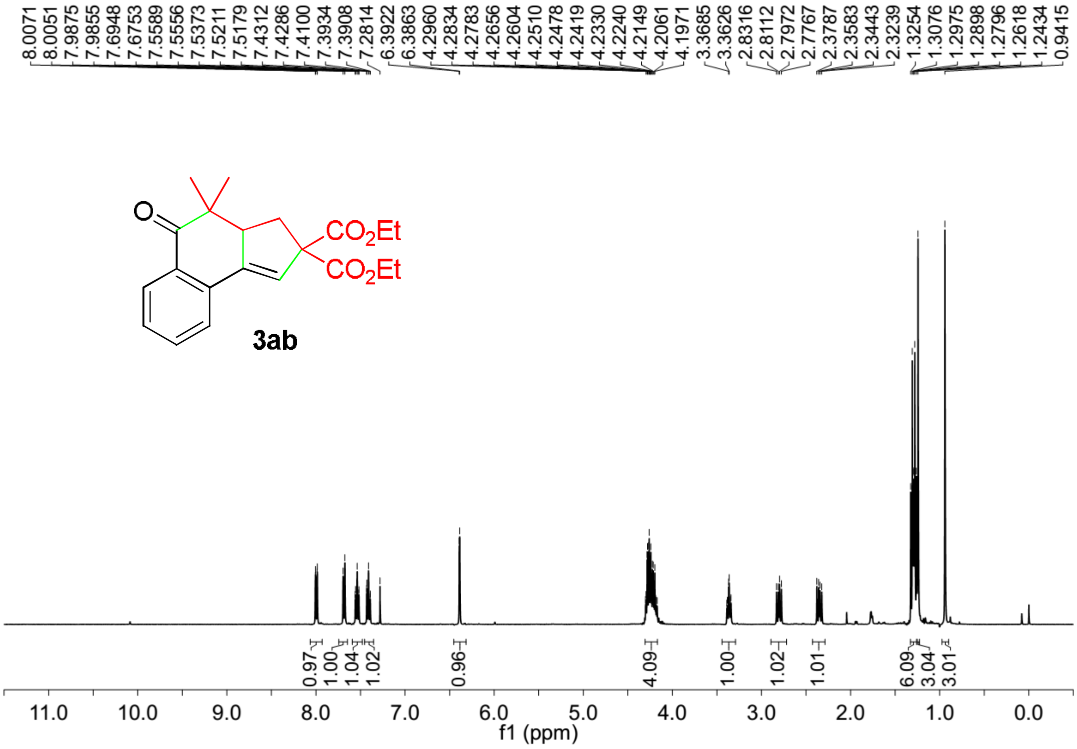
8.0071 8.0051 7.9875 7.9855 7.6948 7.6753 7.5589 7.5556 7.5373 7.5211 7.5179 7.4312 7.4286 7.4100 7.3934 7.3908 7.2814 6.3922 6.3863 4.2960 4.2834 4.2783 4.2656 4.2604 4.2510 4.2478 4.2419 4.2330 4.2240 4.2149 4.2061 4.1971 3.3685 3.3626 2.8316 2.8112 2.7972 2.7767 2.3787 2.3583 2.3443 2.3239 1.3254 1.3076 1.2975 1.2898 1.2796 1.2618 1.2434 0.9415
O
CO2Et
CO2Et
3ab
0.97
1.00
1.04
1.02
0.96
4.09
1.00
1.02
1.01
6.09
3.04
3.01
11.0 10.0 9.0 8.0 7.0 6.0 5.0 4.0 3.0 2.0 1.0 0.0
f1 (ppm)

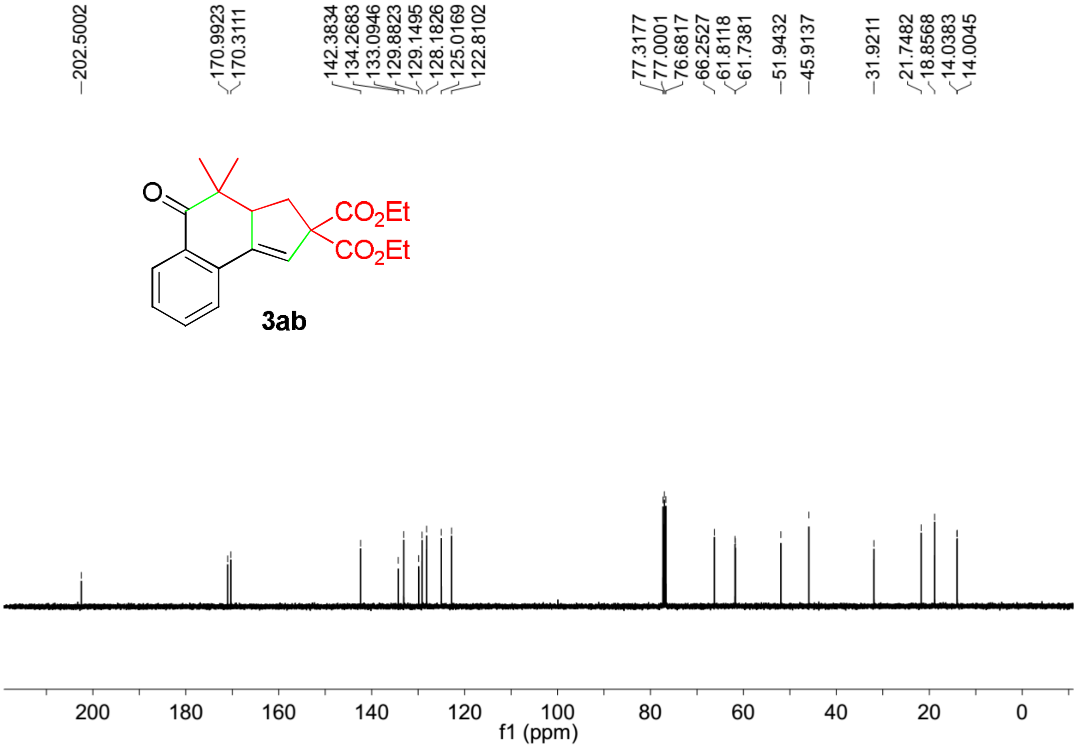
202.5002
170.9923 170.3111
142.3834 134.2683 133.0946 129.8823 129.1495 128.1826 125.0169 122.8102
77.3177 77.0001 76.6817 66.2527 61.8118 61.7381 51.9432 45.9137
31.9211 21.7482 18.8568 14.0383 14.0045
O
CO2Et
CO2Et
3ab
200 180 160 140 120 100 80 60 40 20 0
f1 (ppm)

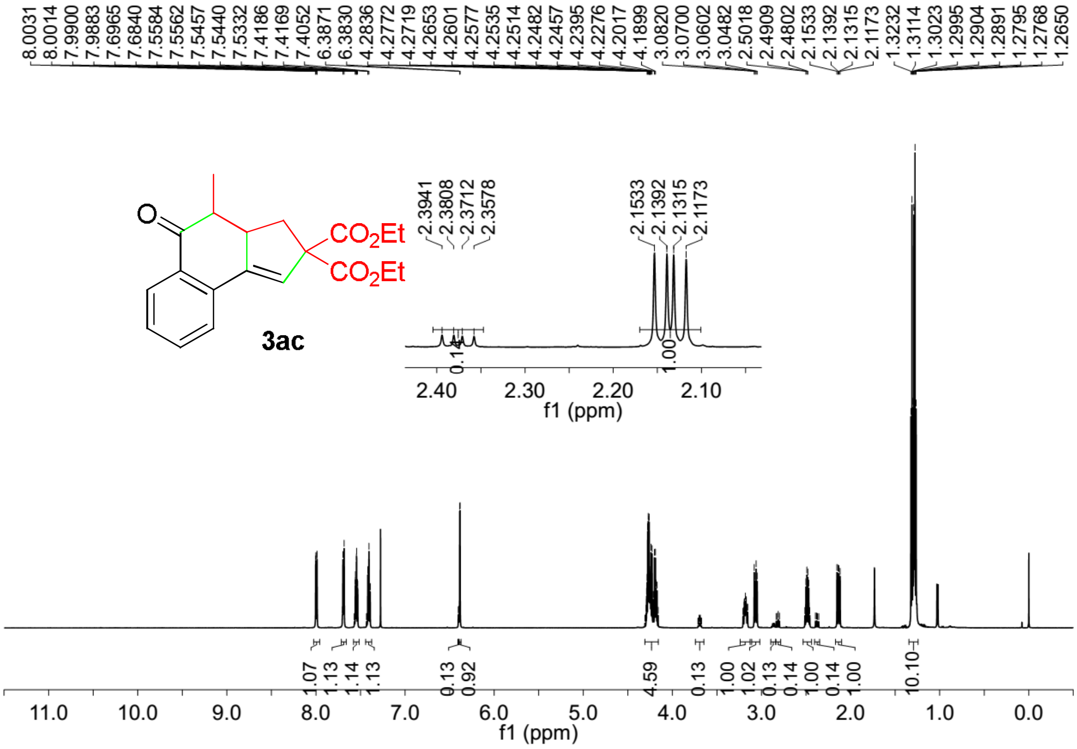

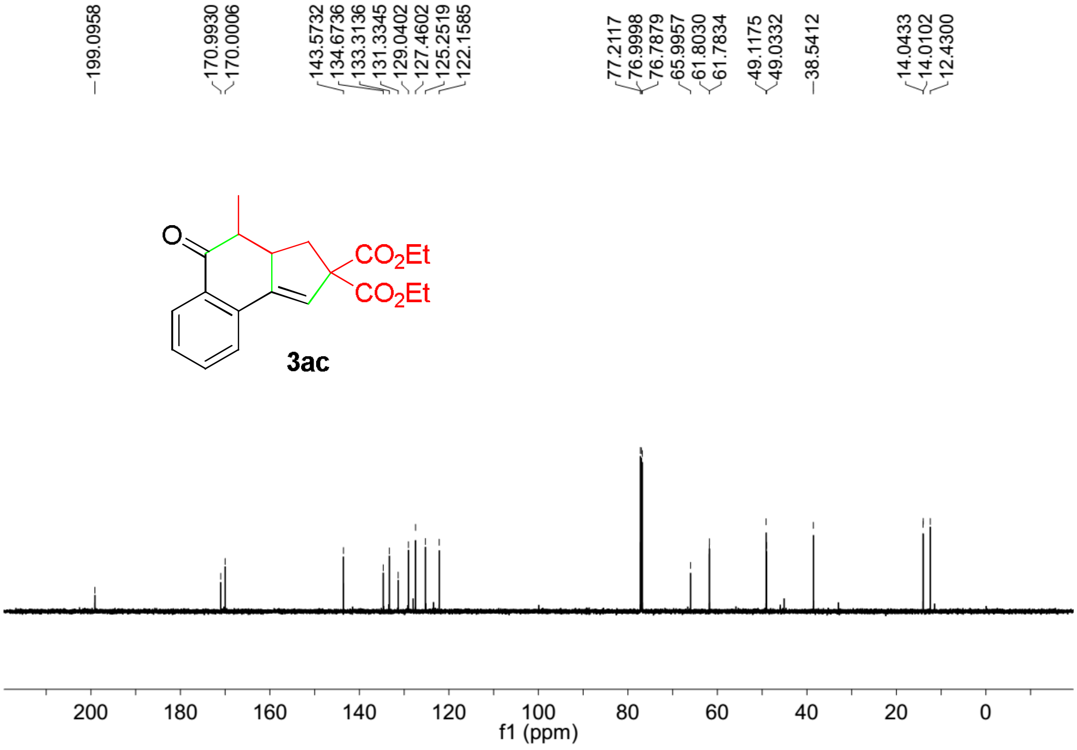

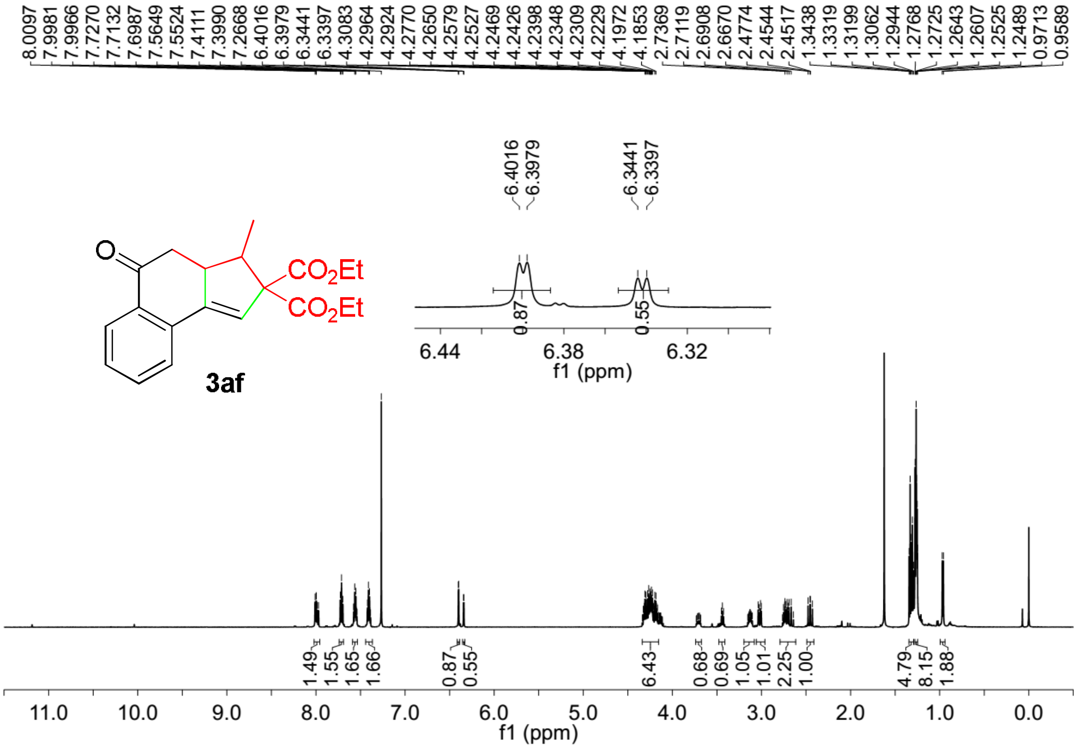
8.0097
7.9981
7.9966
7.7270
7.7132
7.6987
7.5649
7.5524
7.4111
7.3990
7.2668
6.4016
6.3979
6.3441
6.3397
4.3083
4.2964
4.2924
4.2770
4.2650
4.2579
4.2527
4.2469
4.2426
4.2398
4.2348
4.2309
4.2229
4.1972
4.1853
2.7369
2.7119
2.6908
2.6670
2.4774
2.4544
2.4517
1.3438
1.3319
1.3199
1.3062
1.2944
1.2768
1.2725
1.2643
1.2607
1.2525
1.2489
0.9713
0.9589
CO2Et
CO2Et
O
3af
6.4016
6.3979
6.3441
6.3397
0.87
0.55
6.44
6.38
6.32
f1 (ppm)
1.49
1.55
1.65
1.66
0.87
0.55
6.43
0.68
0.69
1.05
1.01
2.25
1.00
4.79
8.15
1.88
11.0
10.0
9.0
8.0
7.0
6.0
5.0
4.0
3.0
2.0
1.0
0.0
f1 (ppm)

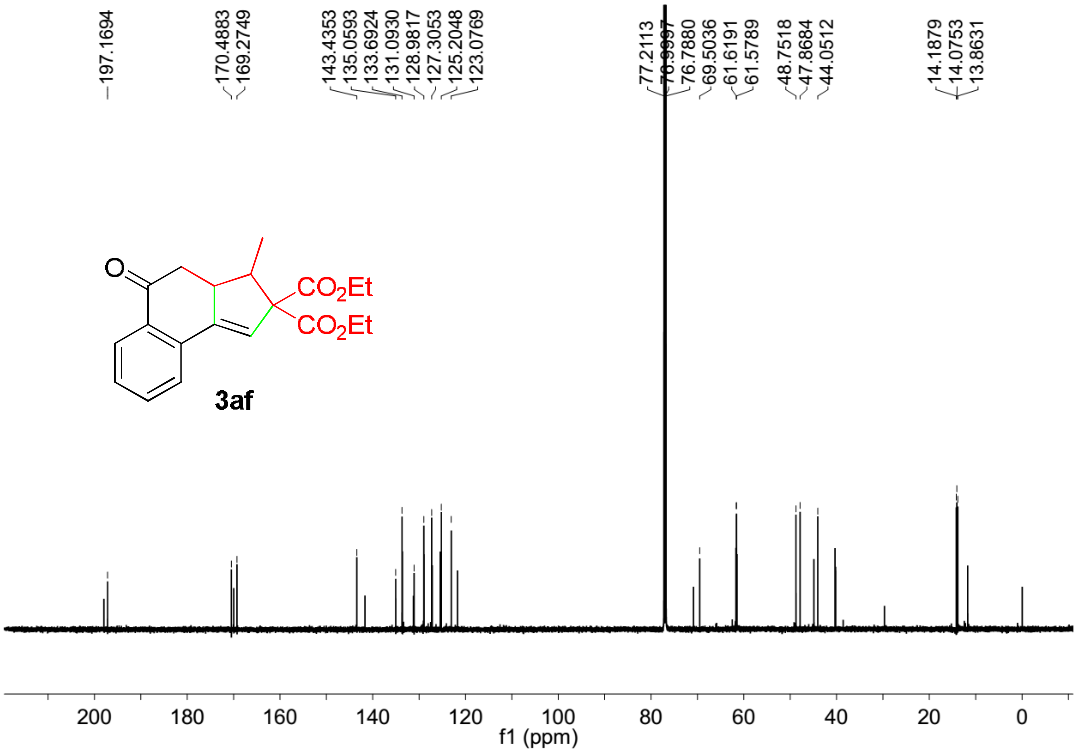
197.1694
170.4883
169.2749
143.4353
135.0593
133.6924
131.0930
128.9817
127.3053
125.2048
123.0769
77.2113
76.9997
76.7880
69.5036
61.6191
61.5789
48.7518
47.8684
44.0512
14.1879
14.0753
13.8631
CO2Et
CO2Et
O
3af
200
180
160
140
120
100
80
60
40
20
0
f1 (ppm)

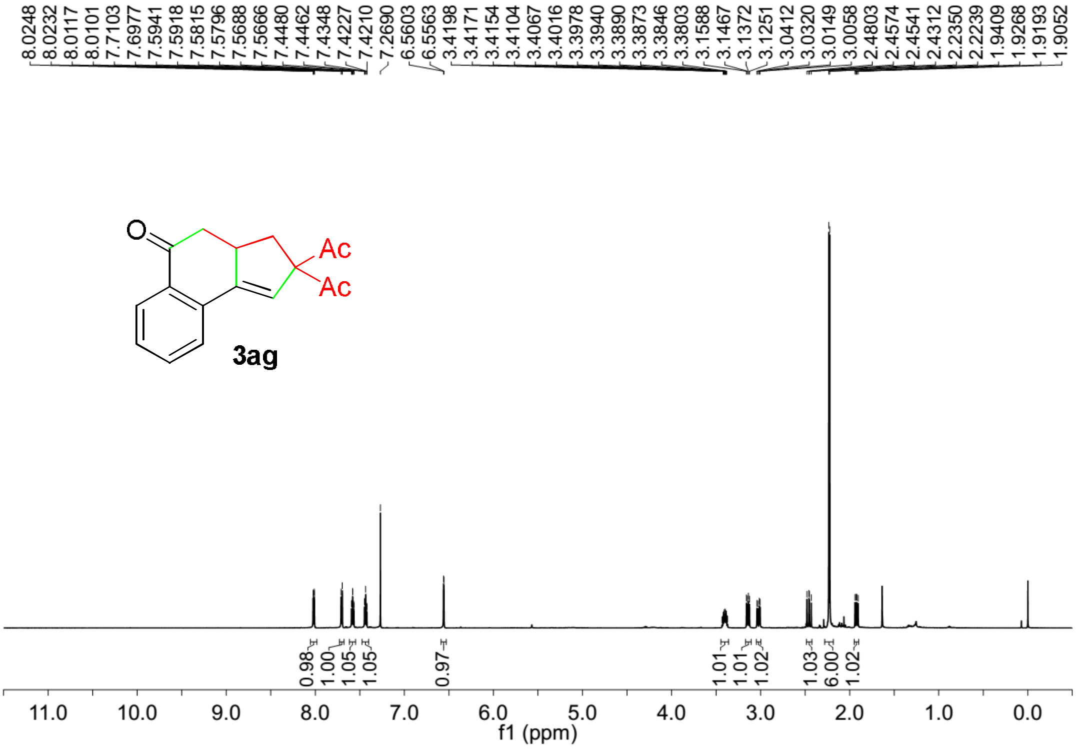
8.0248
8.0232
8.0117
8.0101
7.7103
7.6977
7.5941
7.5918
7.5815
7.5796
7.5688
7.5666
7.4480
7.4462
7.4348
7.4227
7.4210
7.2690
6.5603
6.5563
3.4198
3.4171
3.4154
3.4104
3.4067
3.4016
3.3978
3.3940
3.3890
3.3873
3.3846
3.3803
3.1588
3.1467
3.1372
3.1251
3.0412
3.0320
3.0149
3.0058
2.4803
2.4574
2.4541
2.4312
2.2350
2.2239
1.9409
1.9268
1.9193
1.9052
O
Ac
Ac
3ag
0.98
1.00
1.05
1.05
0.97
1.01
1.01
1.02
1.03
6.00
1.02
11.0
10.0
9.0
8.0
7.0
6.0
5.0
4.0
3.0
2.0
1.0
0.0
f1 (ppm)

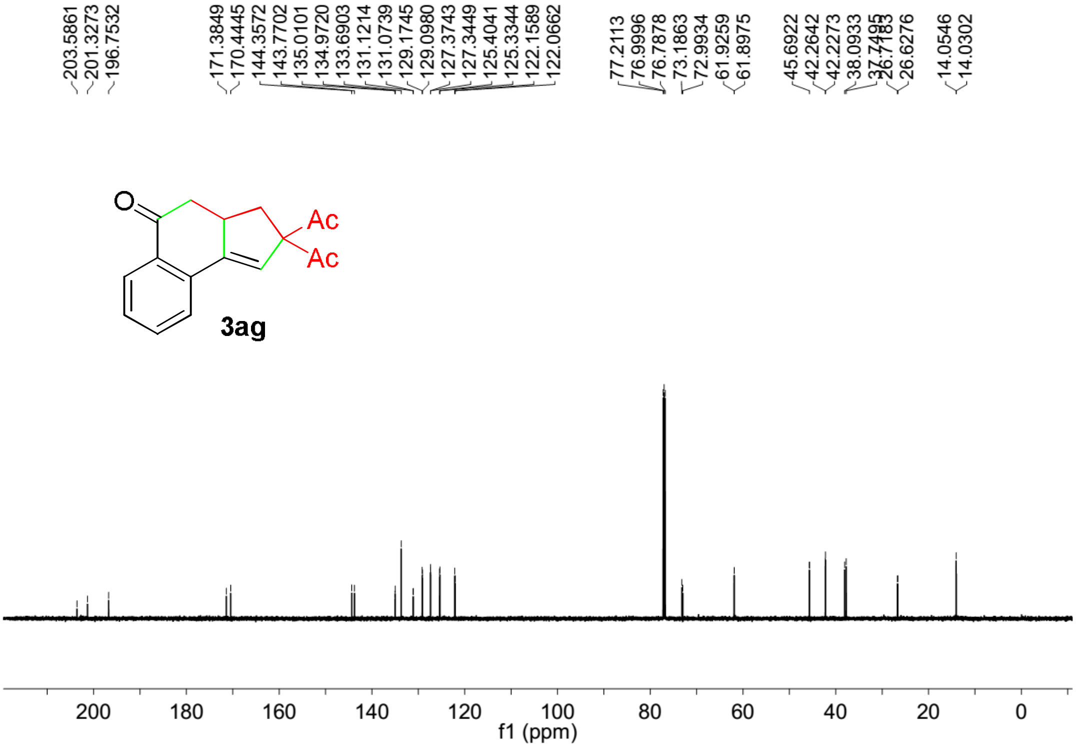
203.5861
201.3273
196.7532
171.3849
170.4445
144.3572
143.7702
135.0101
134.9720
133.6903
131.1214
131.0739
129.1745
129.0980
127.3743
127.3449
125.4041
125.3344
122.1589
122.0662
77.2113
76.9996
76.7878
73.1863
72.9934
61.9259
61.8975
45.6922
42.2642
42.2273
38.0933
37.7495
26.7183
26.6276
14.0546
14.0302
O
Ac
Ac
3ag
200
180
160
140
120
100
80
60
40
20
0
f1 (ppm)

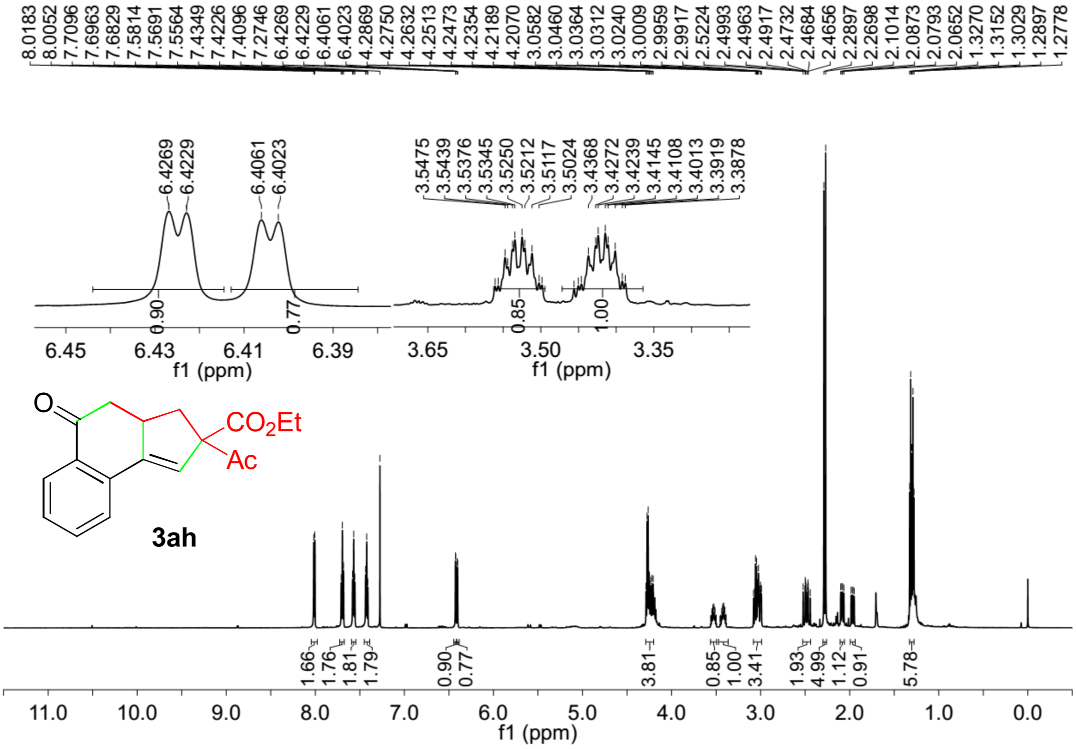
8.0183
8.0052
7.7096
7.6963
7.6829
7.5814
7.5691
7.5564
7.4349
7.4226
7.4096
7.2746
6.4269
6.4229
6.4061
6.4023
4.2869
4.2750
4.2632
4.2513
4.2473
4.2354
4.2189
4.2070
3.0582
3.0460
3.0364
3.0312
3.0240
3.0009
2.9959
2.9917
2.5224
2.4993
2.4963
2.4917
2.4732
2.4684
2.4656
2.2897
2.2698
2.1014
2.0873
2.0793
2.0652
1.3270
1.3152
1.3029
1.2897
1.2778
6.4269
6.4229
6.4061
6.4023
0.90
0.77
6.45
6.43
6.41
6.39
f1 (ppm)
3.5475
3.5439
3.5376
3.5345
3.5250
3.5212
3.5117
3.5024
3.4368
3.4272
3.4239
3.4145
3.4108
3.4013
3.3919
3.3878
0.85
1.00
3.65
3.50
3.35
f1 (ppm)
O
CO_2Et
Ac
3ah
1.66
1.76
1.81
1.79
0.90
0.77
3.81
0.85
1.00
3.41
1.93
4.99
1.12
0.91
5.78
11.0
10.0
9.0
8.0
7.0
6.0
5.0
4.0
3.0
2.0
1.0
0.0
f1 (ppm)

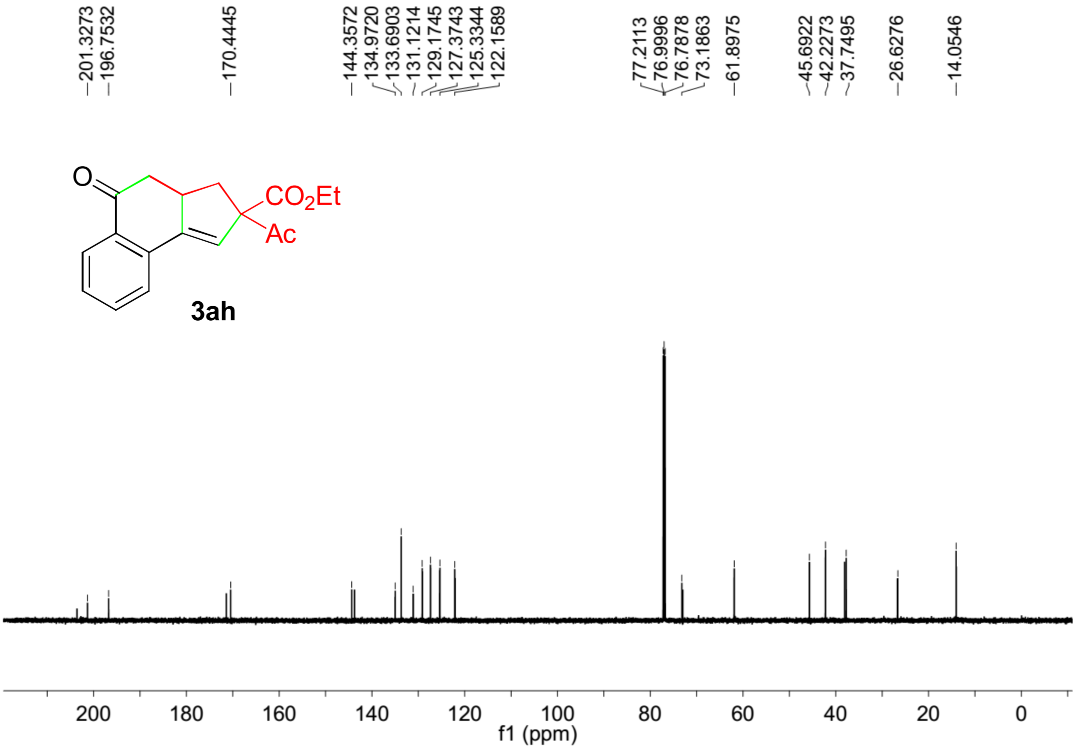
201.3273
196.7532
170.4445
144.3572
134.9720
133.6903
131.1214
129.1745
127.3743
125.3344
122.1589
77.2113
76.9996
76.7878
73.1863
61.8975
45.6922
42.2273
37.7495
26.6276
14.0546
O
CO_2Et
Ac
3ah
200
180
160
140
120
100
80
60
40
20
0
f1 (ppm)

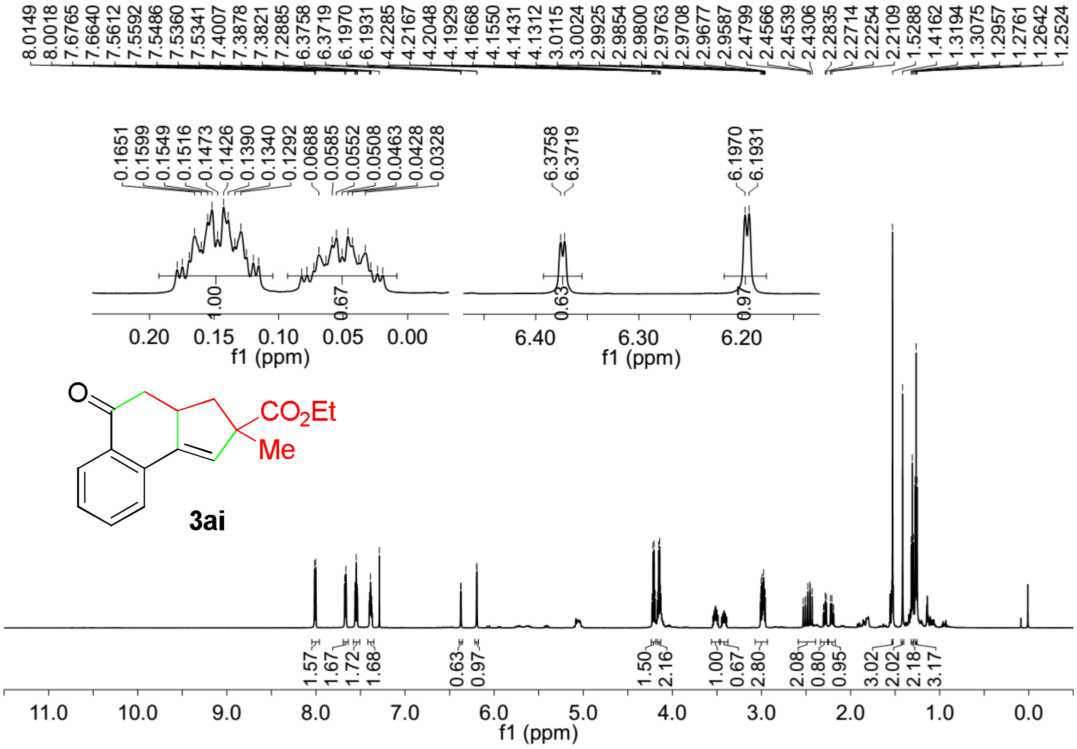

3ai

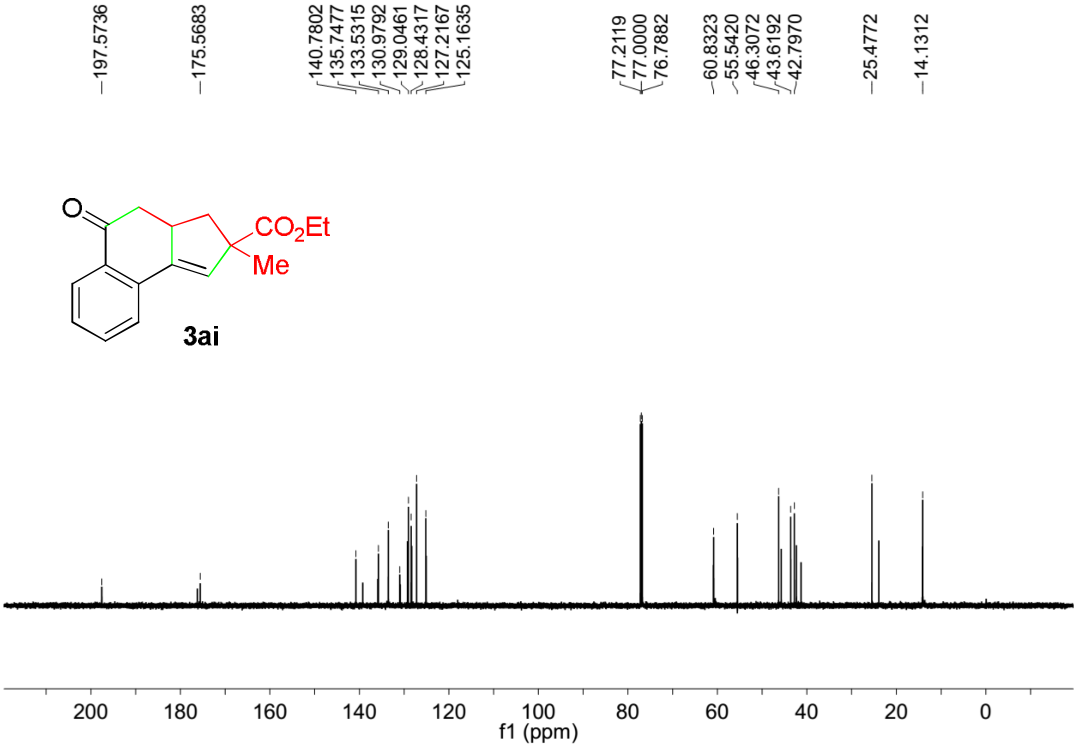

3ai

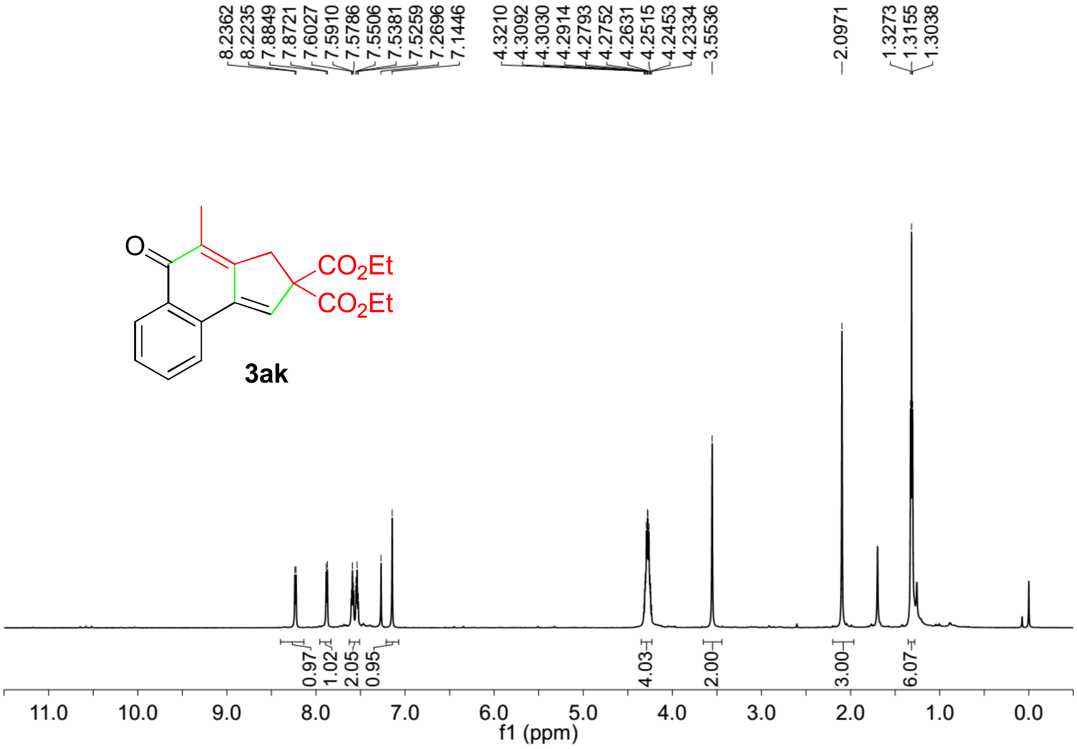
8.2362
8.2235
7.8849
7.8721
7.6027
7.5910
7.5786
7.5506
7.5381
7.5259
7.2696
7.1446
4.3210
4.3092
4.3030
4.2914
4.2793
4.2752
4.2631
4.2515
4.2453
4.2334
3.5536
2.0971
1.3273
1.3155
1.3038
O
CO_2Et
CO_2Et
3ak
0.97
1.02
2.05
0.95
4.03
2.00
3.00
6.07
11.0 10.0 9.0 8.0 7.0 6.0 5.0 4.0 3.0 2.0 1.0 0.0
f1 (ppm)

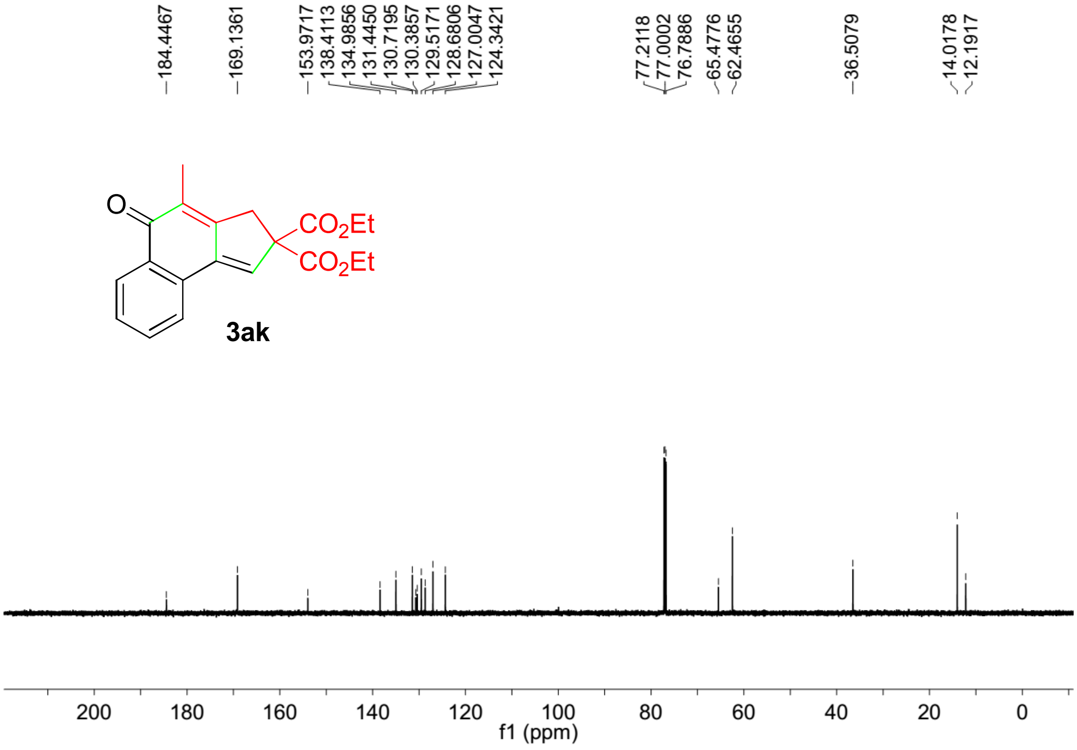
184.4467
169.1361
153.9717
138.4113
134.9856
131.4450
130.7195
130.3857
129.5171
128.6806
127.0047
124.3421
77.2118
77.0002
76.7886
65.4776
62.4655
36.5079
14.0178
12.1917
O
CO_2Et
CO_2Et
3ak
200 180 160 140 120 100 80 60 40 20 0
f1 (ppm)

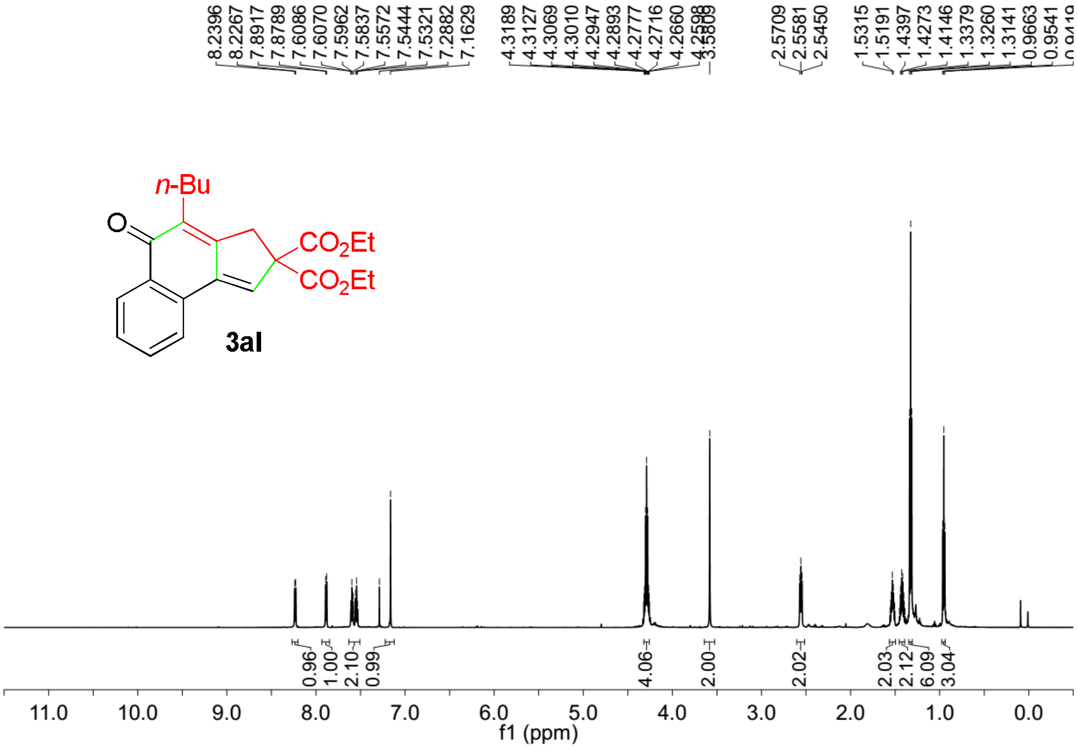
8.2396
8.2267
7.8917
7.8789
7.6086
7.6070
7.5962
7.5837
7.5572
7.5444
7.5321
7.2882
7.1629
4.3189
4.3127
4.3069
4.3010
4.2947
4.2893
4.2777
4.2716
4.2660
2.5709
2.5581
2.5450
1.5315
1.5191
1.4397
1.4273
1.4146
1.3379
1.3260
1.3141
0.9663
0.9541
n-Bu
O
CO_2Et
CO_2Et
3al
0.96
1.00
2.10
0.99
4.06
2.00
2.02
2.03
2.12
6.09
3.04
11.0
10.0
9.0
8.0
7.0
6.0
5.0
4.0
3.0
2.0
1.0
0.0
f1 (ppm)

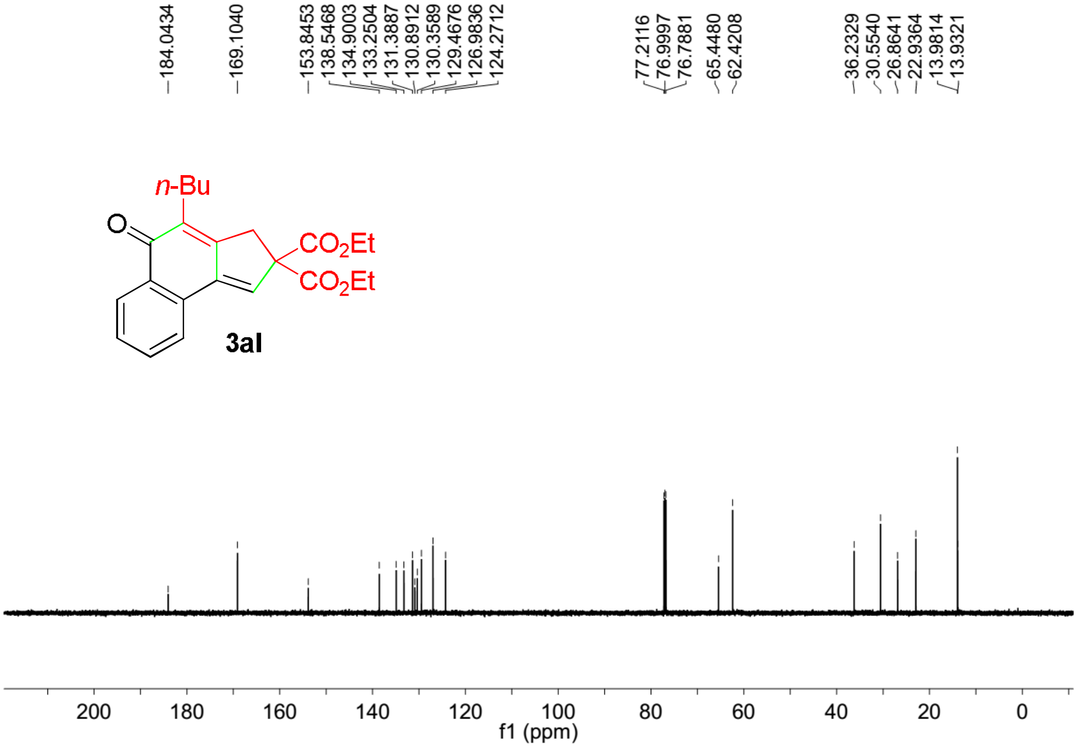
184.0434
169.1040
153.8453
138.5468
134.9003
133.2504
131.3887
130.8912
130.3589
129.4676
126.9836
124.2712
77.2116
76.9997
76.7881
65.4480
62.4208
36.2329
30.5540
26.8641
22.9364
13.9814
13.9321
n-Bu
O
CO_2Et
CO_2Et
3al
200
180
160
140
120
100
80
60
40
20
0
f1 (ppm)

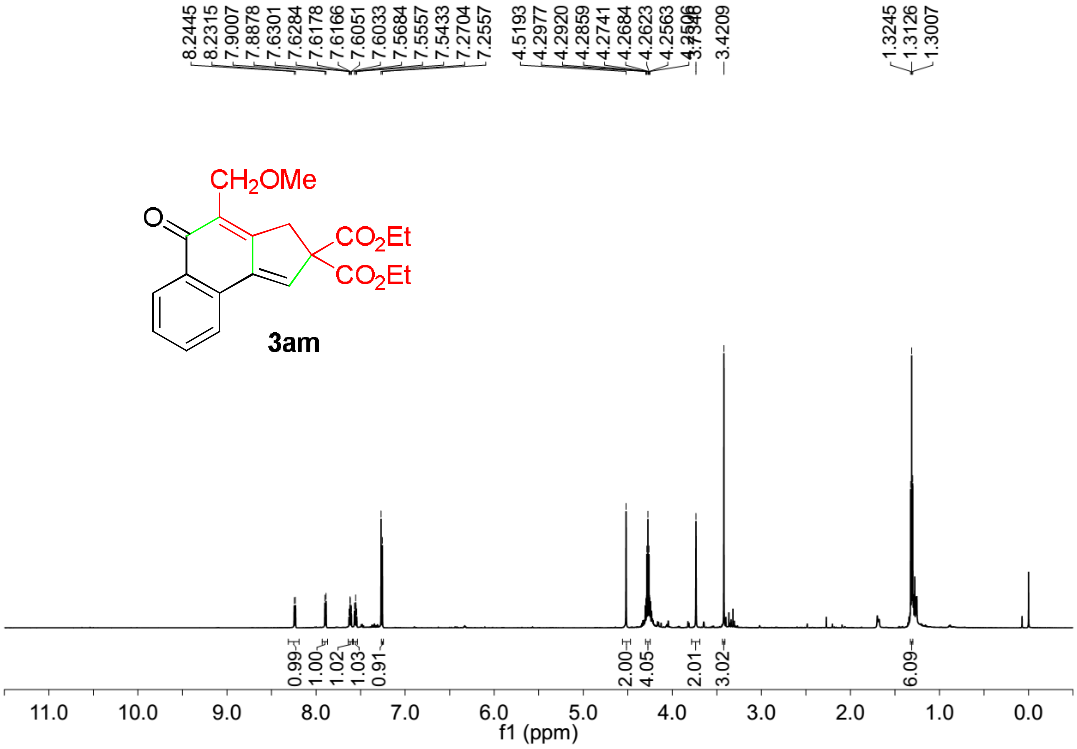
8.2445
8.2315
7.9007
7.8878
7.6301
7.6284
7.6178
7.6166
7.6051
7.6033
7.5684
7.5557
7.5433
7.2704
7.2557
4.5193
4.2977
4.2920
4.2859
4.2741
4.2684
4.2623
4.2563
3.4209
1.3245
1.3126
1.3007
CH2OMe
O
CO2Et
CO2Et
3am
0.99
1.00
1.02
1.03
0.91
2.00
4.05
2.01
3.02
6.09
11.0
10.0
9.0
8.0
7.0
6.0
5.0
4.0
3.0
2.0
1.0
0.0
f1 (ppm)

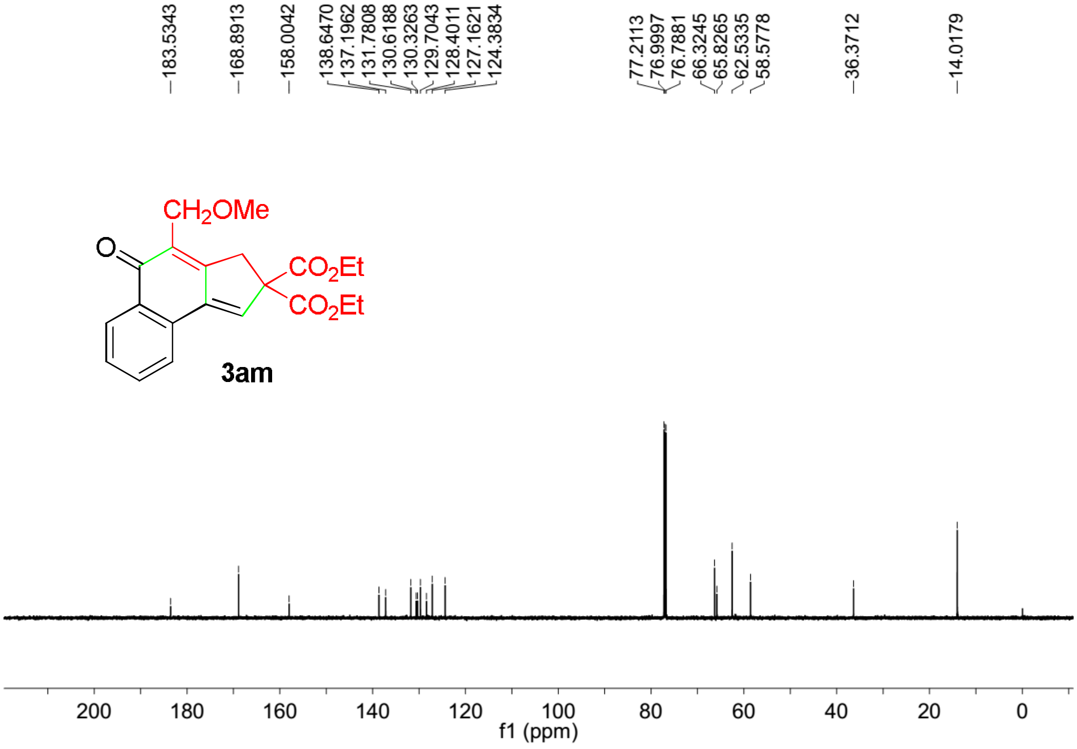
183.5343
168.8913
158.0042
138.6470
137.1962
131.7808
130.6188
130.3263
129.7043
128.4011
127.1621
124.3834
77.2113
76.9997
76.7881
66.3245
65.8265
62.5335
58.5778
36.3712
14.0179
CH2OMe
O
CO2Et
CO2Et
3am
200
180
160
140
120
100
80
60
40
20
0
f1 (ppm)

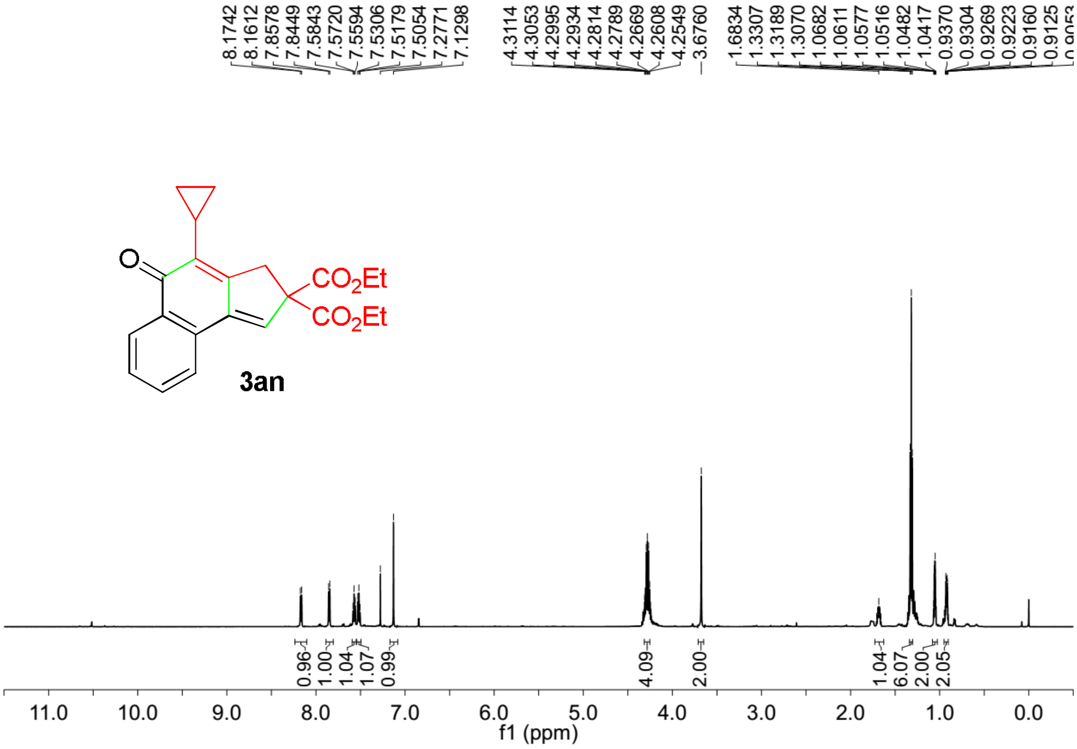
8.1742
8.1612
7.8578
7.8449
7.5843
7.5720
7.5594
7.5306
7.5179
7.5054
7.2771
7.1298
4.3114
4.3053
4.2995
4.2934
4.2814
4.2789
4.2669
4.2608
4.2549
3.6760
1.6834
1.3307
1.3189
1.3070
1.0682
1.0611
1.0577
1.0516
1.0482
1.0417
0.9370
0.9304
0.9269
0.9223
0.9160
0.9125
CO2Et
CO2Et
3an
0.96
1.00
1.04
1.07
0.99
4.09
2.00
1.04
6.07
2.00
2.05
11.0
10.0
9.0
8.0
7.0
6.0
5.0
4.0
3.0
2.0
1.0
0.0
f1 (ppm)

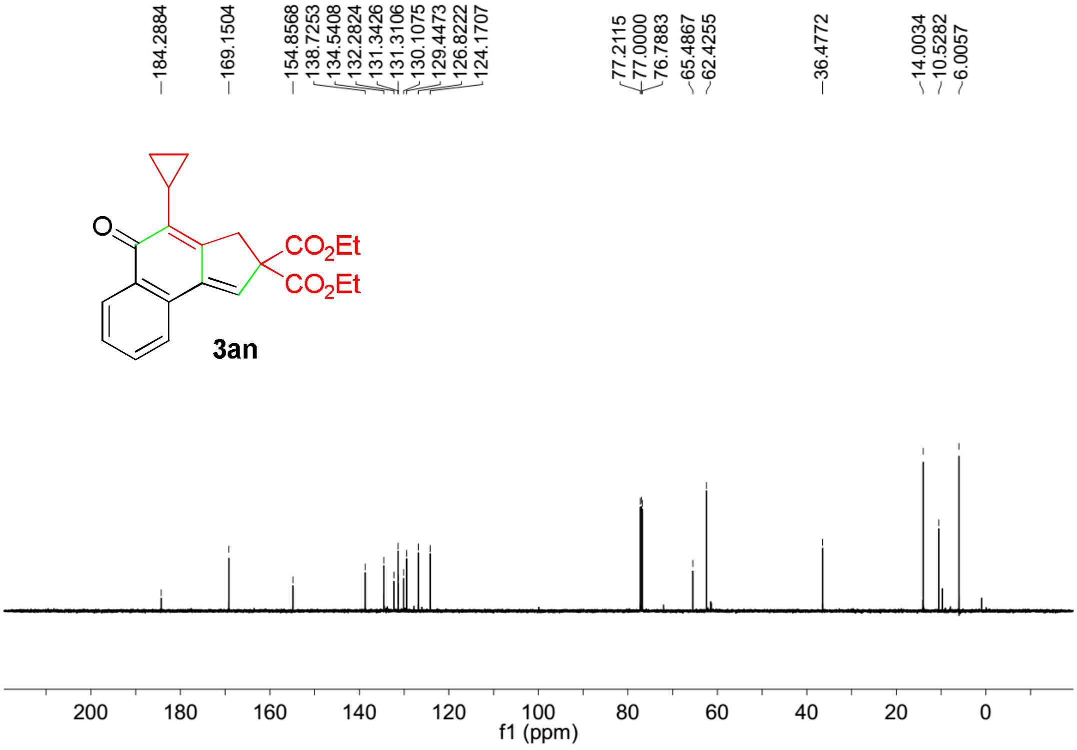
184.2884
169.1504
154.8568
138.7253
134.5408
132.2824
131.3426
131.3106
130.1075
129.4473
126.8222
124.1707
77.2115
77.0000
76.7883
65.4867
62.4255
36.4772
14.0034
10.5282
6.0057
CO2Et
CO2Et
3an
200
180
160
140
120
100
80
60
40
20
0
f1 (ppm)

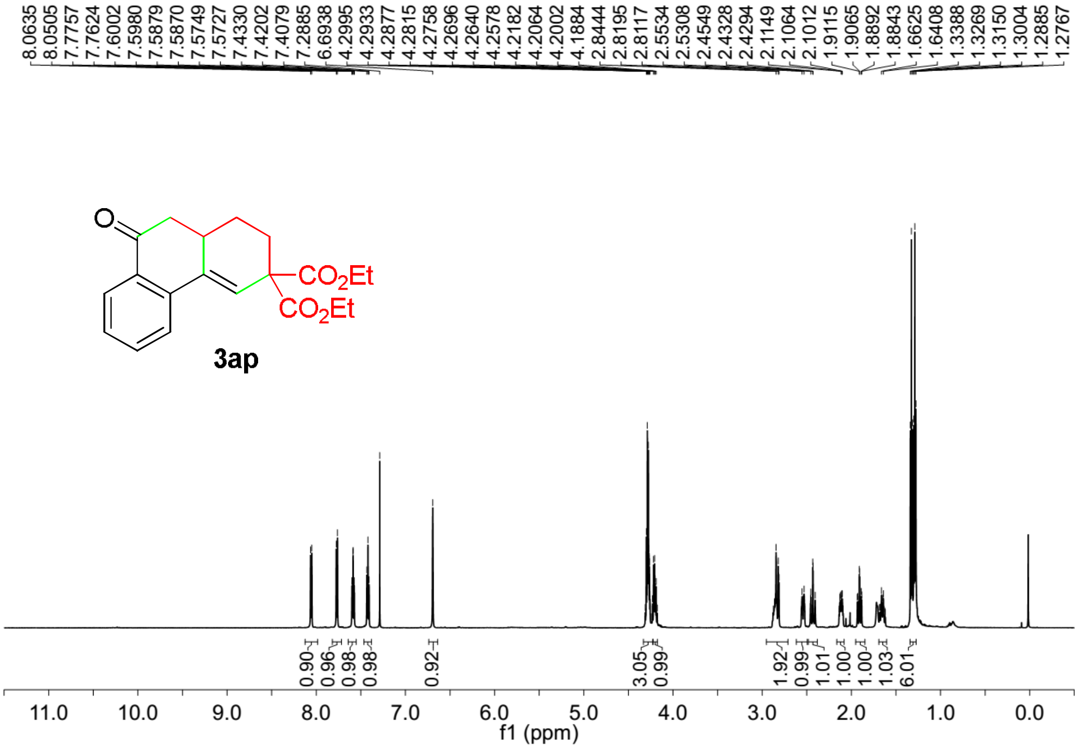
8.0635 8.0505 7.7757 7.7624 7.6002 7.5980 7.5879 7.5870 7.5749 7.5727 7.4330 7.4202 7.4079 7.2885 6.6938 4.2995 4.2933 4.2877 4.2815 4.2758 4.2696 4.2640 4.2578 4.2182 4.2064 4.2002 4.1884 2.8444 2.8195 2.8117 2.5534 2.5308 2.4549 2.4328 2.4294 2.1149 2.1064 2.1012 1.9115 1.9065 1.8892 1.8843 1.6625 1.6408 1.3388 1.3269 1.3150 1.3004 1.2885 1.2767
O
CO2Et
CO2Et
3ap
0.90 0.96 0.98 0.98 0.92 3.05 0.99 1.92 0.99 1.01 1.00 1.00 1.03 6.01
11.0 10.0 9.0 8.0 7.0 6.0 5.0 4.0 3.0 2.0 1.0 0.0
f1 (ppm)

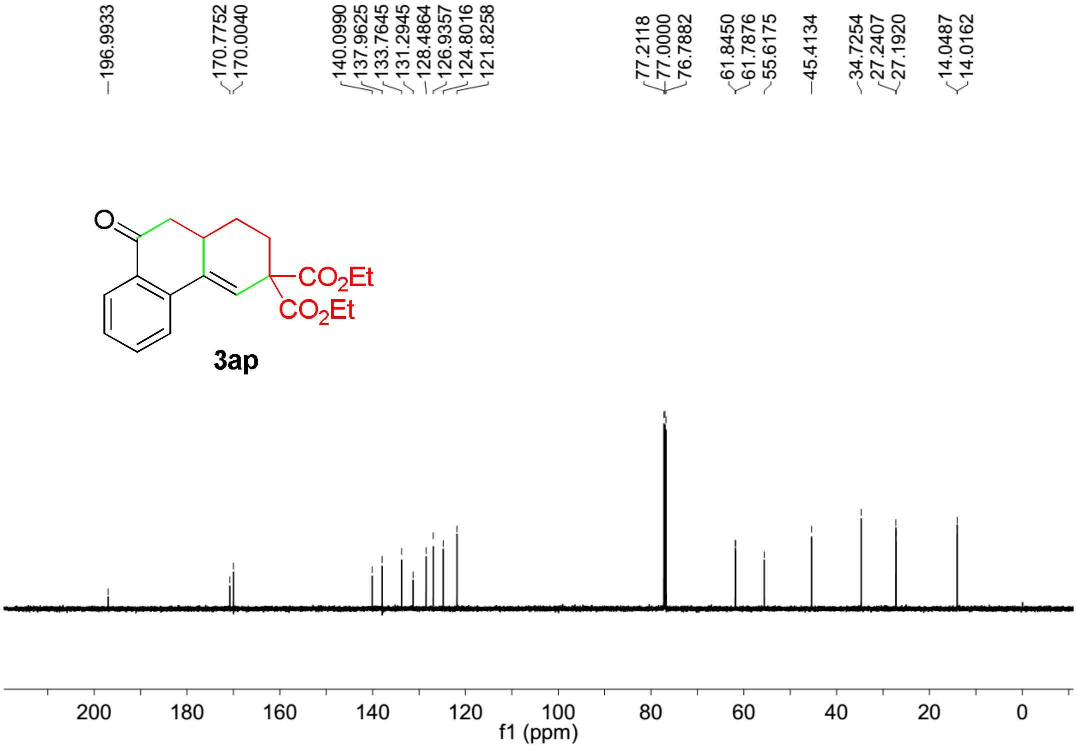
196.9933
170.7752 170.0040
140.0990 137.9625 133.7645 131.2945 128.4864 126.9357 124.8016 121.8258
77.2118 77.0000 76.7882
61.8450 61.7876 55.6175
45.4134
34.7254 27.2407 27.1920
14.0487 14.0162
O
CO2Et
CO2Et
3ap
200 180 160 140 120 100 80 60 40 20 0
f1 (ppm)

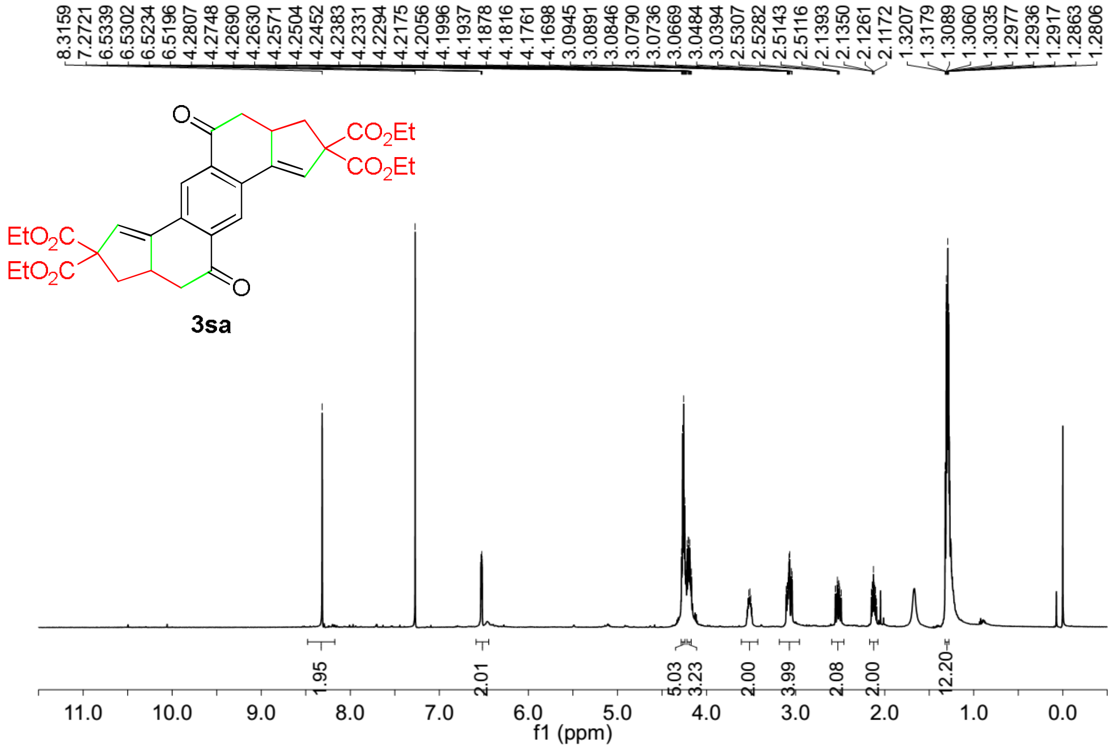
8.3159
7.2721
6.5339
6.5302
6.5234
6.5196
4.2807
4.2748
4.2690
4.2630
4.2571
4.2504
4.2452
4.2383
4.2331
4.2294
4.2175
4.2056
4.1996
4.1937
4.1878
4.1816
4.1761
4.1698
3.0945
3.0891
3.0846
3.0790
3.0736
3.0669
3.0484
3.0394
2.5307
2.5282
2.5143
2.5116
2.1393
2.1350
2.1261
2.1172
1.3207
1.3179
1.3089
1.3060
1.3035
1.2977
1.2936
1.2917
1.2863
1.2806
CO2Et
CO2Et
EtO2C
EtO2C
3sa
1.95
2.01
5.03
3.23
2.00
3.99
2.08
2.00
12.20
f1 (ppm)

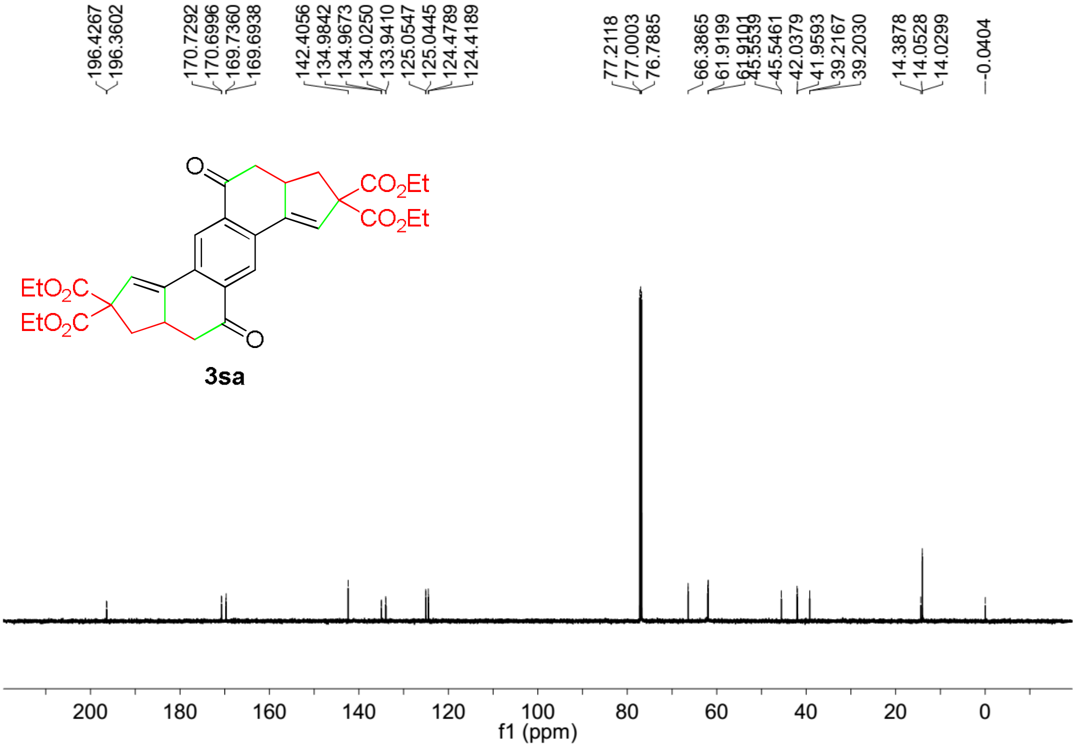
196.4267
196.3602
170.7292
170.6996
169.7360
169.6938
142.4056
134.9842
134.9673
134.0250
133.9410
125.0547
125.0445
124.4789
124.4189
77.2118
77.0003
76.7885
66.3865
61.9199
61.9101
45.5539
45.5461
42.0379
41.9593
39.2167
39.2030
14.3878
14.0528
14.0299
-0.0404
CO2Et
CO2Et
EtO2C
EtO2C
3sa
f1 (ppm)

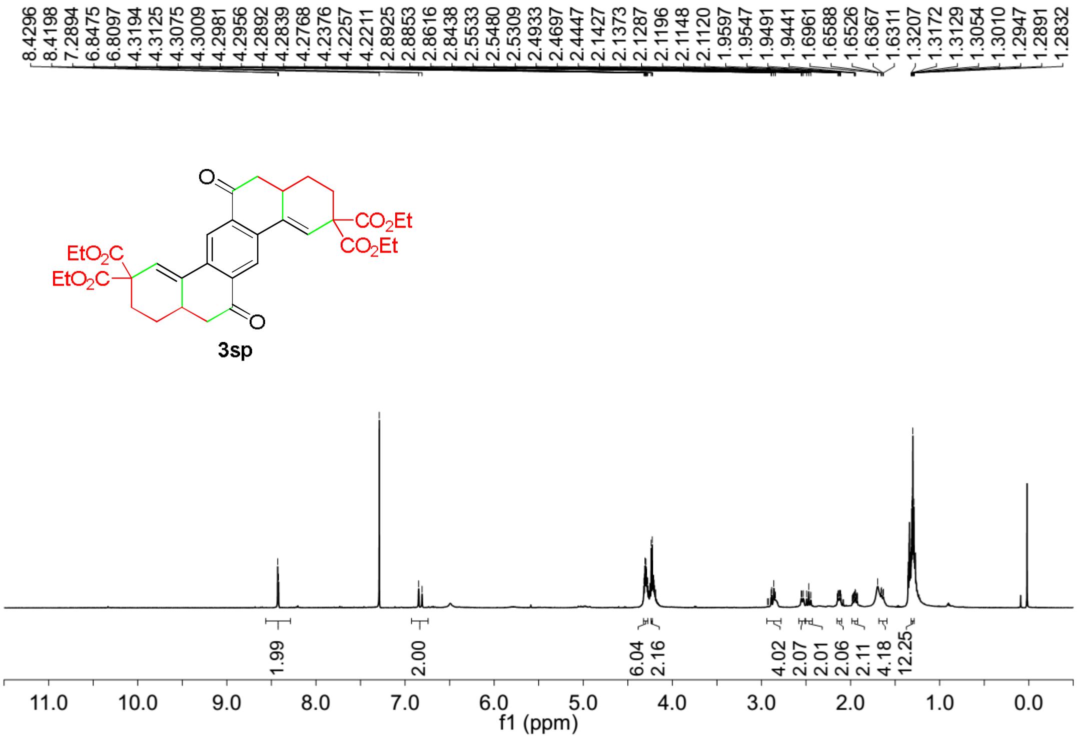

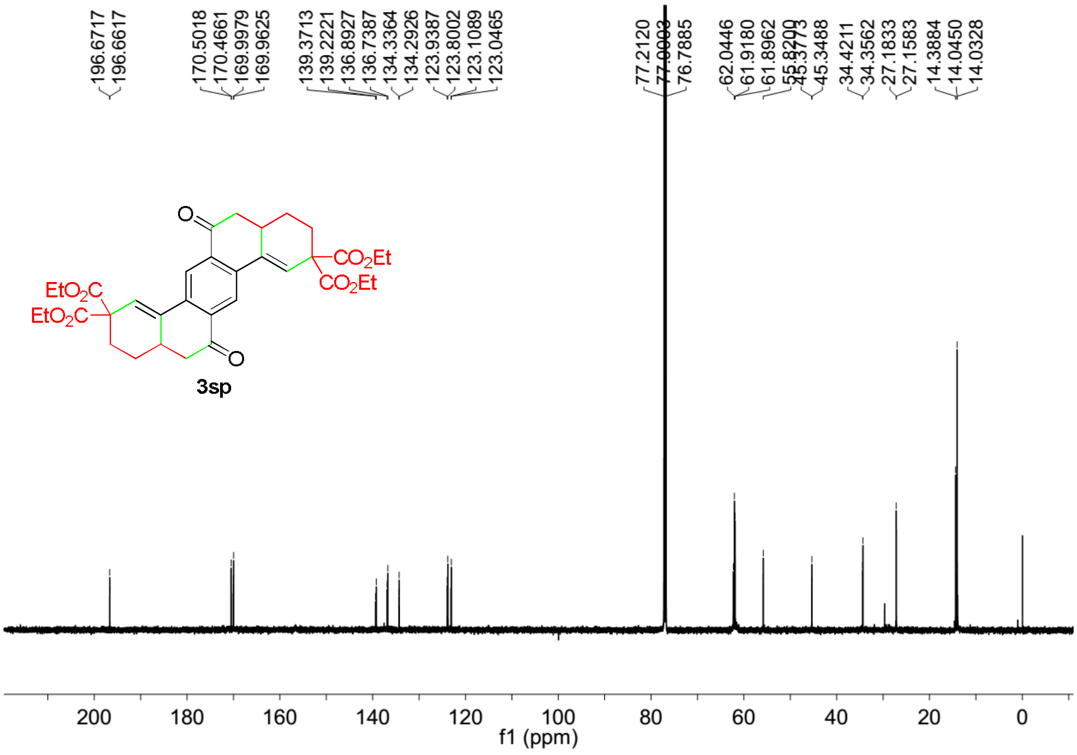

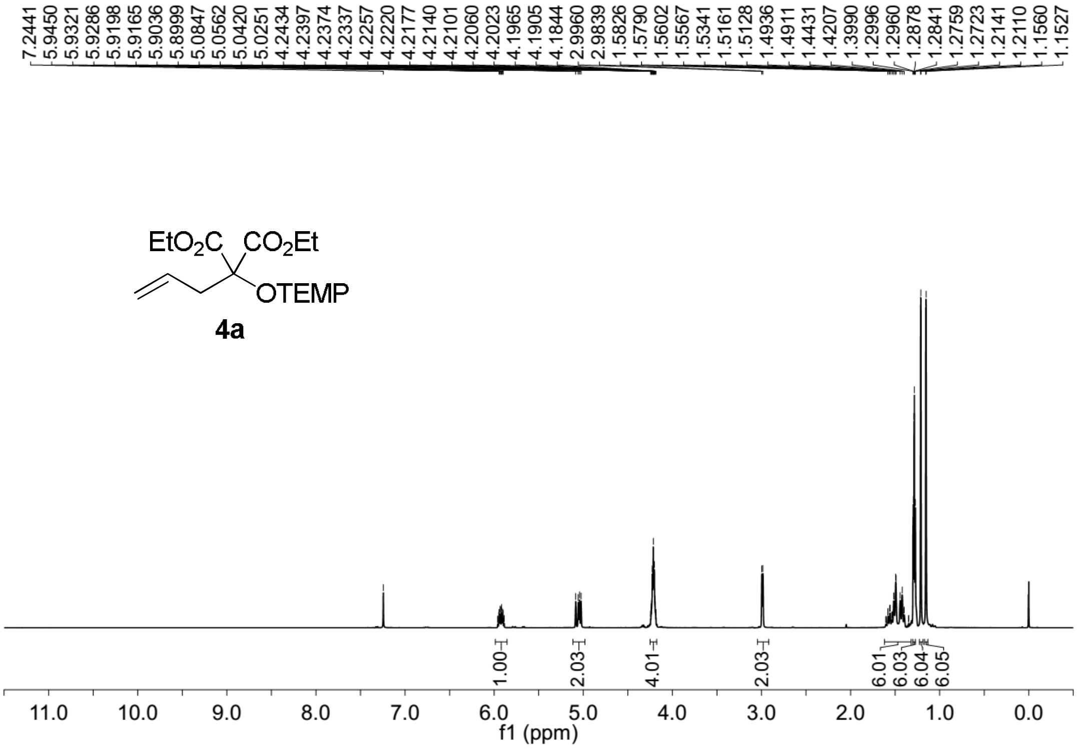
7.2441
5.9450
5.9321
5.9286
5.9198
5.9165
5.9036
5.8899
5.0847
5.0562
5.0420
5.0251
4.2434
4.2397
4.2374
4.2337
4.2257
4.2220
4.2177
4.2140
4.2101
4.2060
4.2023
4.1965
4.1905
4.1844
2.9960
2.9839
1.5826
1.5790
1.5602
1.5567
1.5341
1.5161
1.5128
1.4936
1.4911
1.4431
1.4207
1.3990
1.2996
1.2960
1.2878
1.2841
1.2759
1.2723
1.2141
1.2110
1.1560
1.1527
EtO2C CO2Et
OTEMP
4a
1.00
2.03
4.01
2.03
6.01
6.03
6.04
6.05
11.0 10.0 9.0 8.0 7.0 6.0 5.0 4.0 3.0 2.0 1.0 0.0
f1 (ppm)

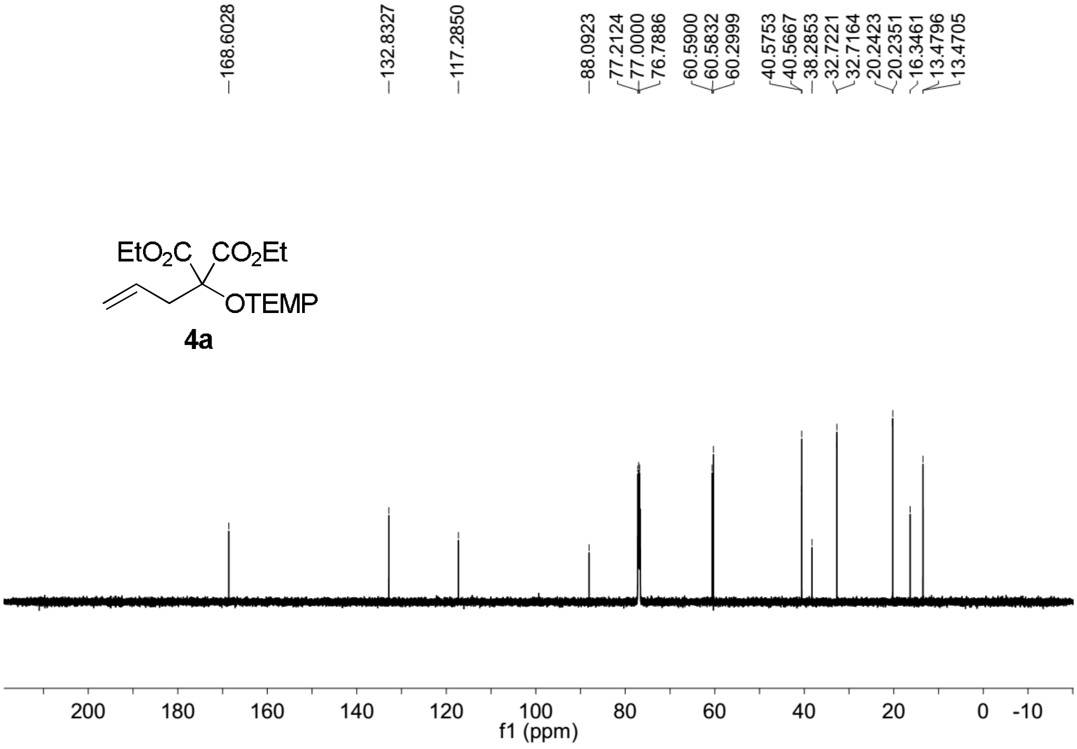
168.6028
132.8327
117.2850
88.0923
77.2124
77.0000
76.7886
60.5900
60.5832
60.2999
40.5753
40.5667
38.2853
32.7221
32.7164
20.2423
20.2351
16.3461
13.4796
13.4705
EtO2C CO2Et
OTEMP
4a
200 180 160 140 120 100 80 60 40 20 0 -10
f1 (ppm)

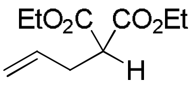
EtO2C CO2Et
H

4b

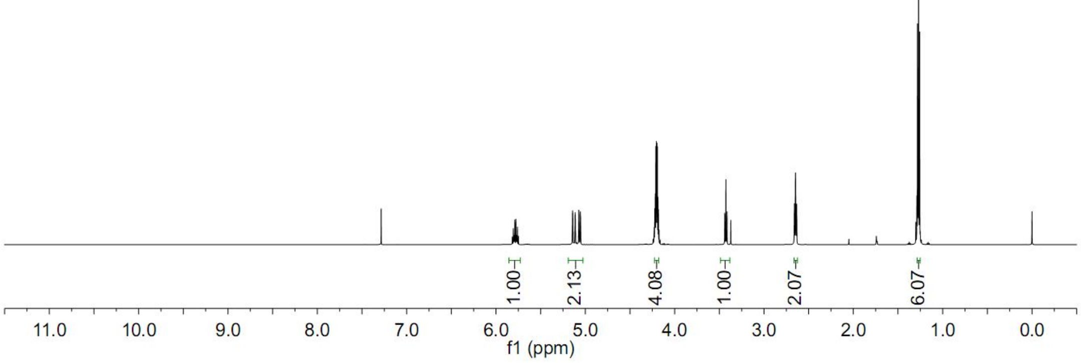
7.2851
5.8057
5.7943
5.7887
5.7773
5.7717
5.7658
5.7602
5.1427
5.0726
5.0556
4.2106
4.2049
4.1987
3.4391
3.4265
3.4139
2.6587
2.6471
2.6347
1.2813
1.2694
1.2575
1.00
2.13
4.08
1.00
2.07
6.07
11.0 10.0 9.0 8.0 7.0 6.0 5.0 4.0 3.0 2.0 1.0 0.0
f1 (ppm)

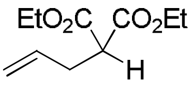
EtO2C CO2Et
H

4b

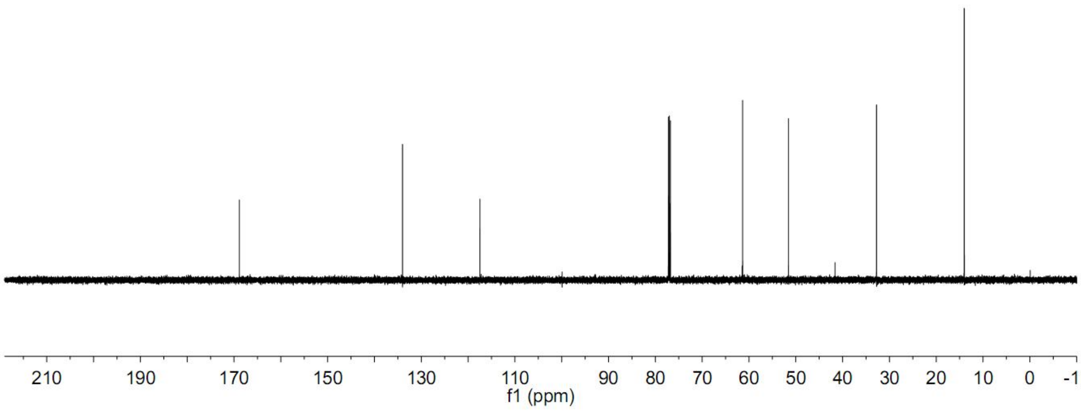
168.8715
134.0044
117.4725
77.2122
77.0004
76.7884
61.3564
51.5683
32.7584
14.0238
210 190 170 150 130 110 90 80 70 60 50 40 30 20 10 0 -1
f1 (ppm)

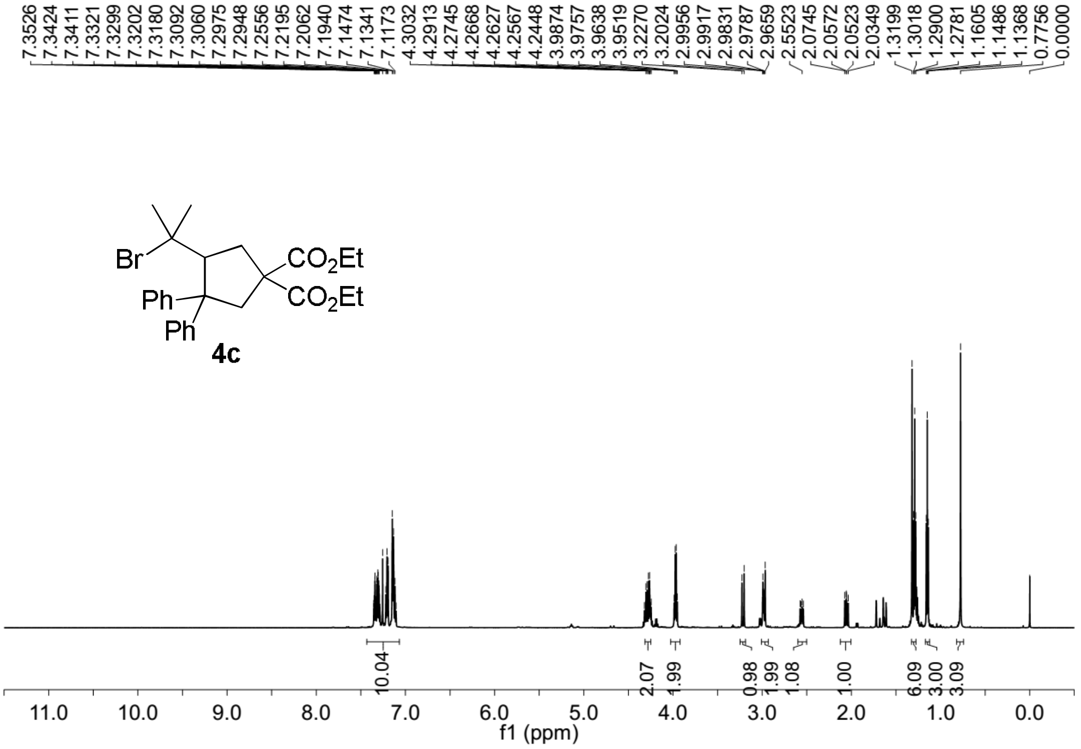

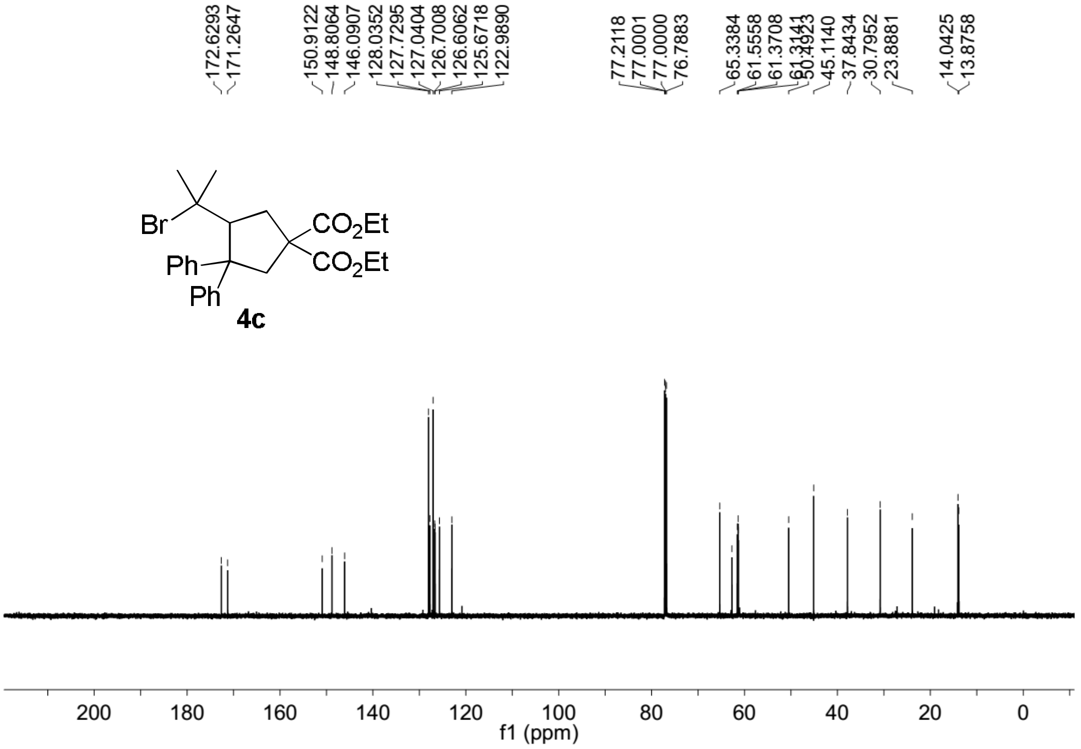

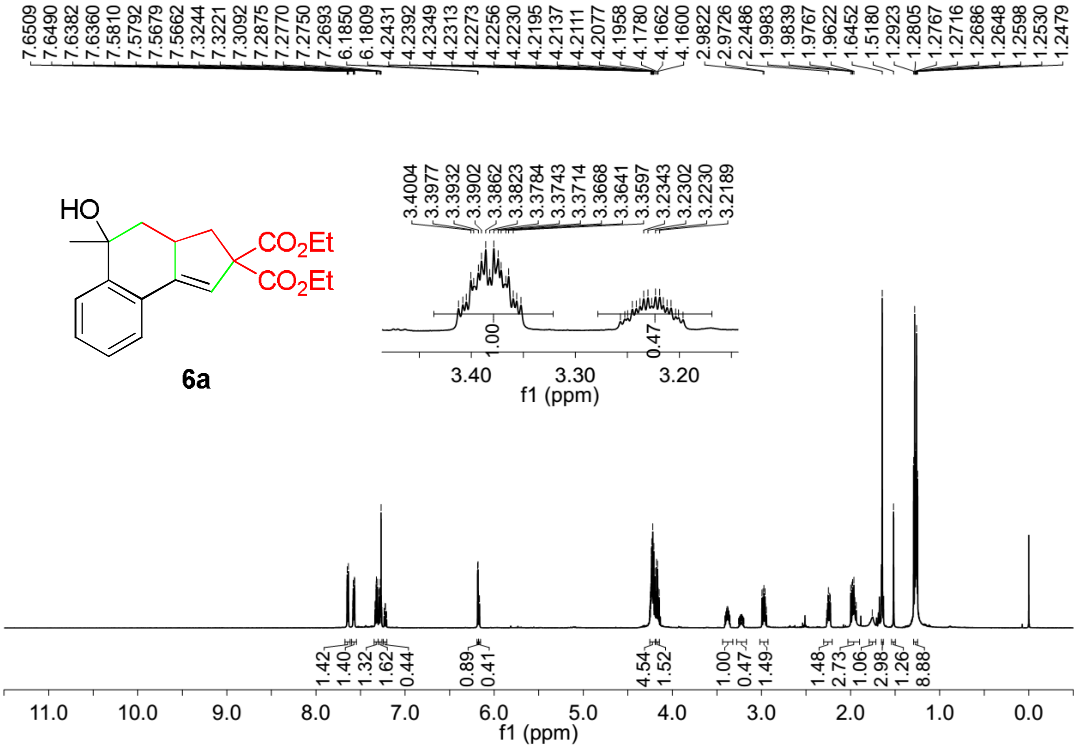
7.6509
7.6490
7.6382
7.6360
7.5810
7.5792
7.5679
7.5662
7.3244
7.3221
7.3092
7.2875
7.2770
7.2750
7.2693
6.1850
6.1809
4.2431
4.2392
4.2349
4.2313
4.2273
4.2256
4.2230
4.2195
4.2137
4.2111
4.2077
4.1958
4.1780
4.1662
4.1600
2.9822
2.9726
2.2486
1.9983
1.9839
1.9767
1.9622
1.6452
1.5180
1.2923
1.2805
1.2767
1.2716
1.2686
1.2648
1.2598
1.2530
1.2479
HO
CO2Et
CO2Et
6a
3.4004
3.3977
3.3932
3.3902
3.3862
3.3823
3.3784
3.3743
3.3714
3.3668
3.3641
3.3597
3.2343
3.2302
3.2230
3.2189
1.00
0.47
3.40
3.30
3.20
f1 (ppm)
1.42
1.40
1.32
1.62
0.44
0.89
0.41
4.54
1.52
1.00
0.47
1.49
1.48
2.73
1.06
2.98
1.26
8.88
11.0
10.0
9.0
8.0
7.0
6.0
5.0
4.0
3.0
2.0
1.0
0.0
f1 (ppm)

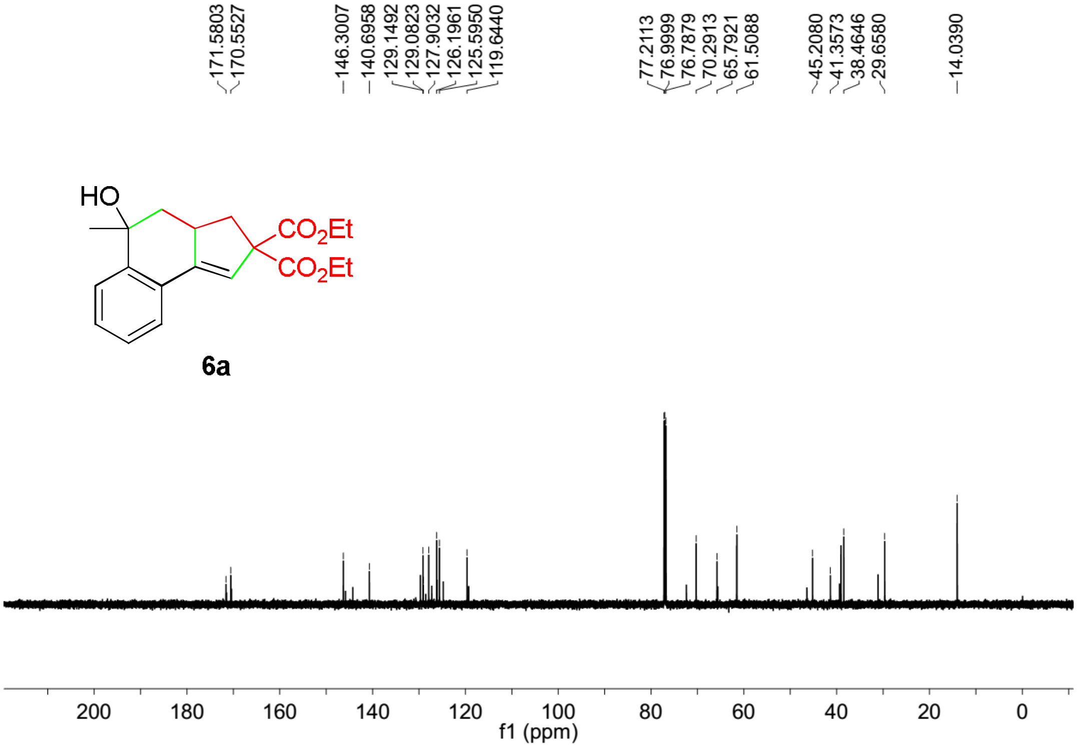
171.5803
170.5527
146.3007
140.6958
129.1492
129.0823
127.9032
126.1961
125.5950
119.6440
77.2113
76.9999
76.7879
70.2913
65.7921
61.5088
45.2080
41.3573
38.4646
29.6580
14.0390
HO
CO2Et
CO2Et
6a
200
180
160
140
120
100
80
60
40
20
0
f1 (ppm)

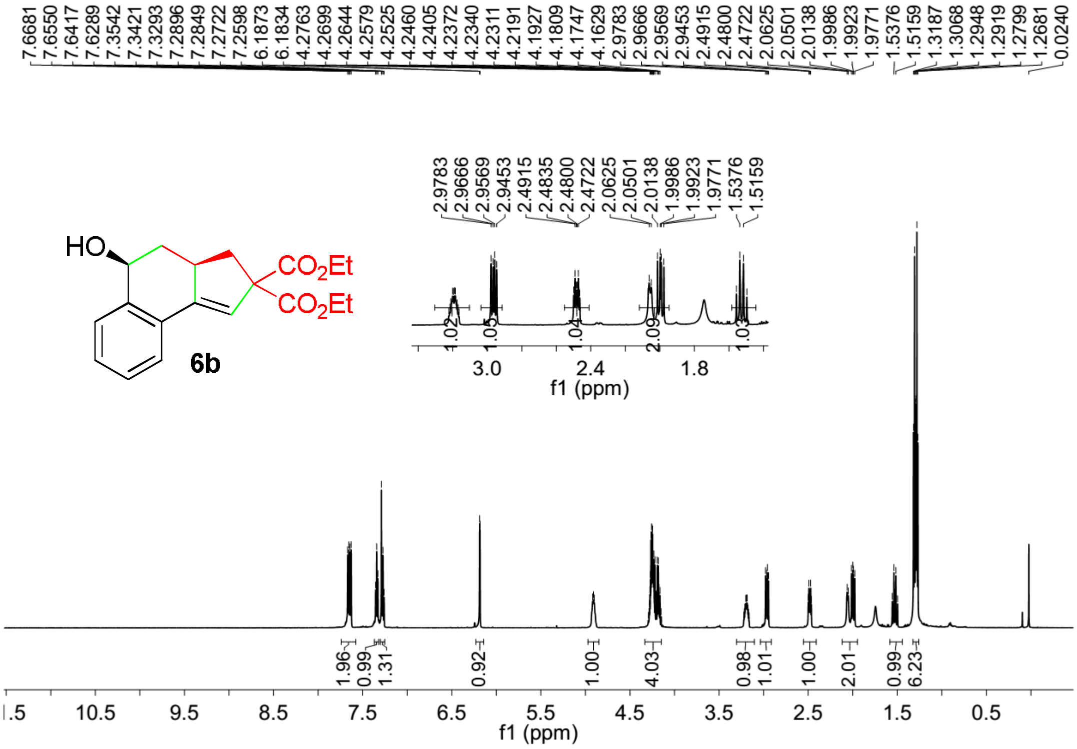

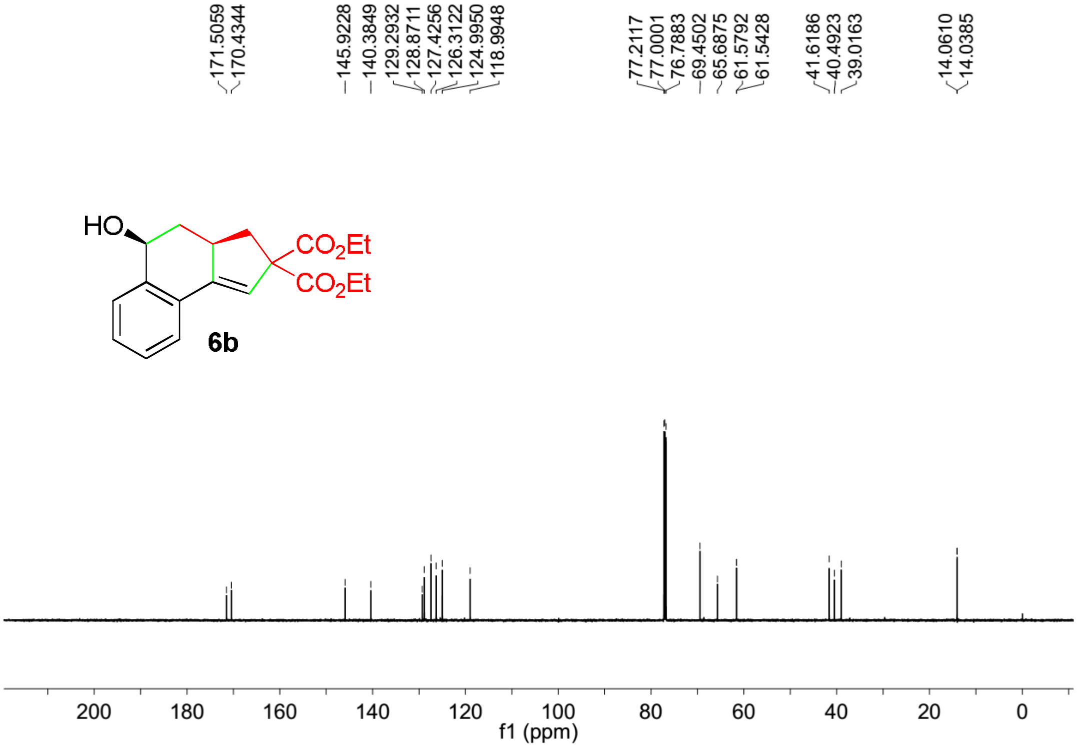

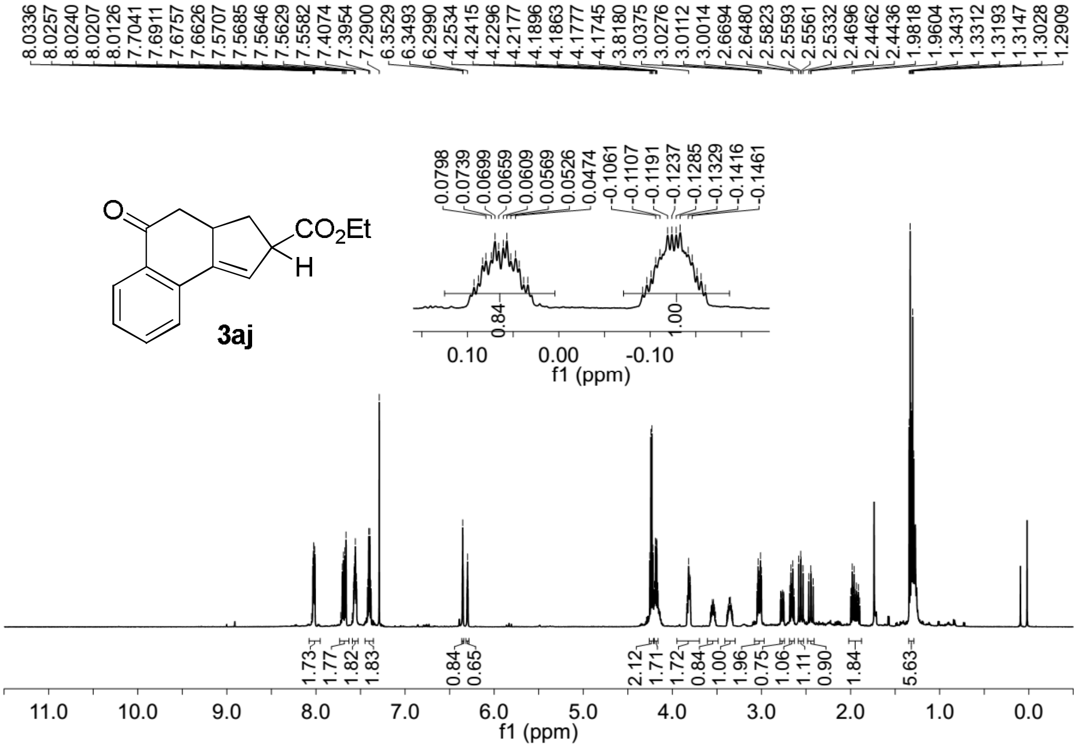
8.0336
8.0257
8.0240
8.0207
8.0126
7.7041
7.6911
7.6757
7.6626
7.5707
7.5685
7.5646
7.5629
7.5582
7.4074
7.3954
7.2900
6.3529
6.3493
6.2990
4.2534
4.2415
4.2296
4.2177
4.1896
4.1863
4.1777
4.1745
3.8180
3.0375
3.0276
3.0112
3.0014
2.6694
2.6480
2.5823
2.5593
2.5561
2.5332
2.4696
2.4462
2.4436
1.9818
1.9604
1.3431
1.3312
1.3193
1.3147
1.3028
1.2909
0.0798
0.0739
0.0699
0.0659
0.0609
0.0569
0.0526
0.0474
-0.1061
-0.1107
-0.1191
-0.1237
-0.1285
-0.1329
-0.1416
-0.1461
O
CO2Et
H
3aj
0.84
1.00
0.10
0.00
-0.10
f1 (ppm)
1.73
1.77
1.82
1.83
0.84
0.65
2.12
1.71
1.72
0.84
1.00
1.96
0.75
1.06
1.11
0.90
1.84
5.63
11.0
10.0
9.0
8.0
7.0
6.0
5.0
4.0
3.0
2.0
1.0
0.0
f1 (ppm)

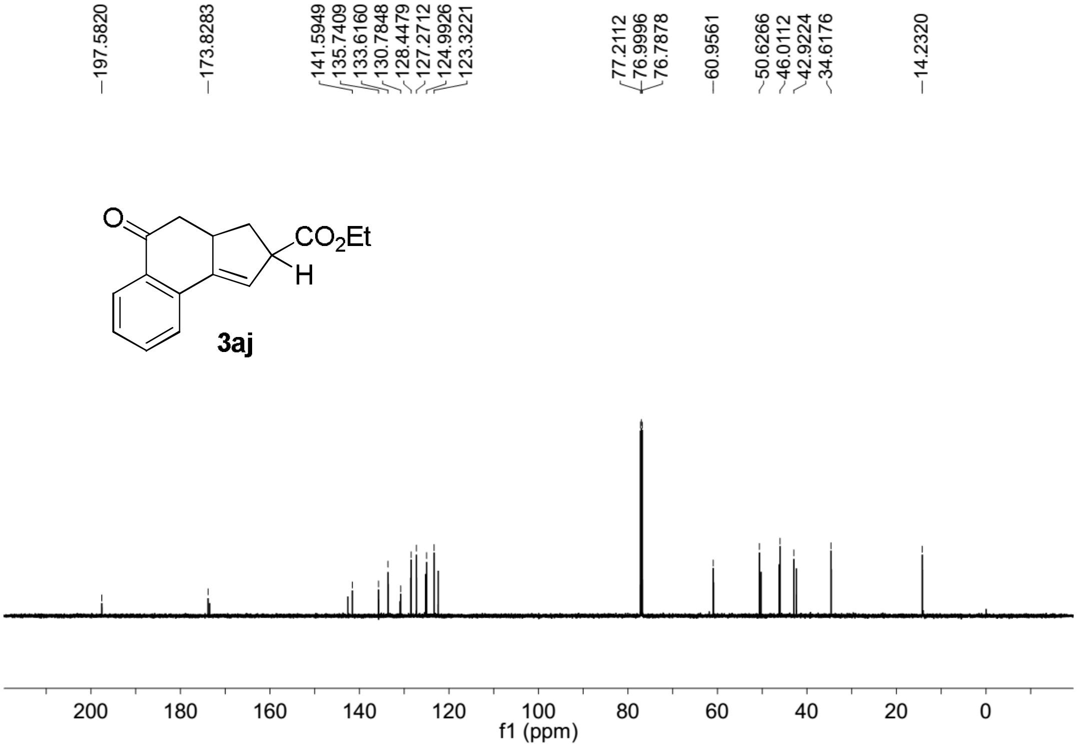
197.5820
173.8283
141.5949
135.7409
133.6160
130.7848
128.4479
127.2712
124.9926
123.3221
77.2112
76.9996
76.7878
60.9561
50.6266
46.0112
42.9224
34.6176
14.2320
O
CO2Et
H
3aj
200
180
160
140
120
100
80
60
40
20
0
f1 (ppm)

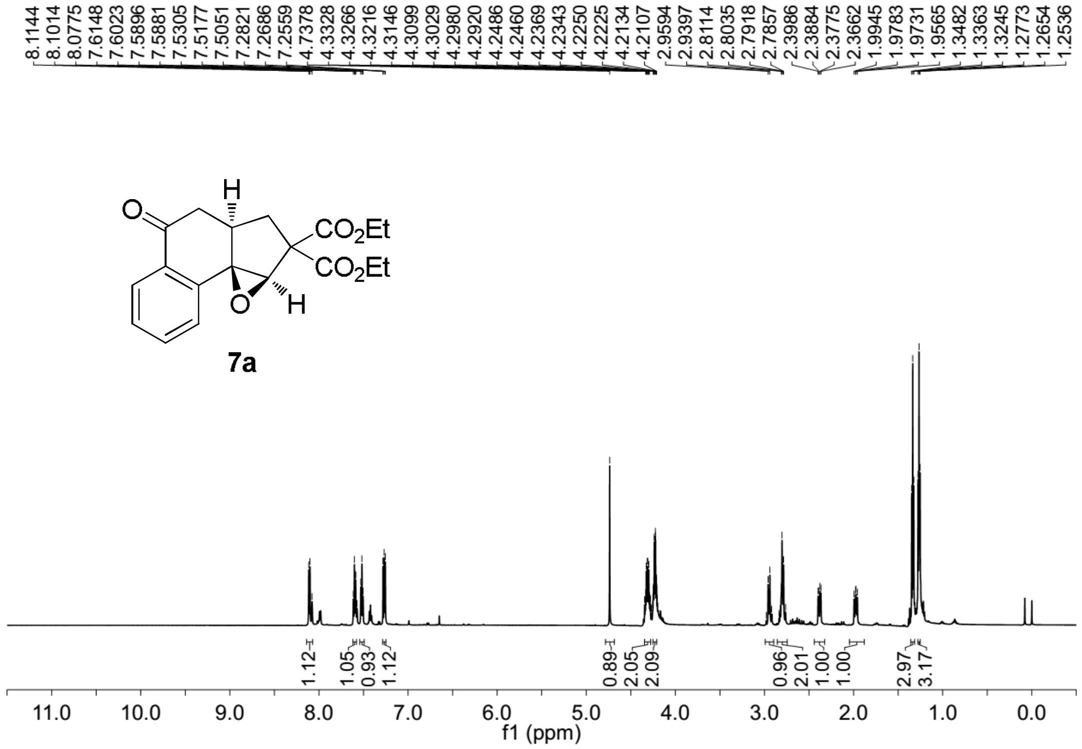
8.1144
8.1014
8.0775
7.6148
7.6023
7.5896
7.5881
7.5305
7.5177
7.5051
7.2821
7.2686
7.2559
4.7378
4.3328
4.3266
4.3216
4.3146
4.3099
4.3029
4.2980
4.2920
4.2486
4.2460
4.2369
4.2343
4.2250
4.2225
4.2134
4.2107
2.9594
2.9397
2.8114
2.8035
2.7918
2.7857
2.3986
2.3884
2.3775
2.3662
1.9945
1.9783
1.9731
1.9565
1.3482
1.3363
1.3245
1.2773
1.2654
1.2536
H
O
CO_2Et
CO_2Et
O
H
7a
1.12
1.05
0.93
1.12
0.89
2.05
2.09
0.96
2.01
1.00
1.00
2.97
3.17
11.0 10.0 9.0 8.0 7.0 6.0 5.0 4.0 3.0 2.0 1.0 0.0
f1 (ppm)

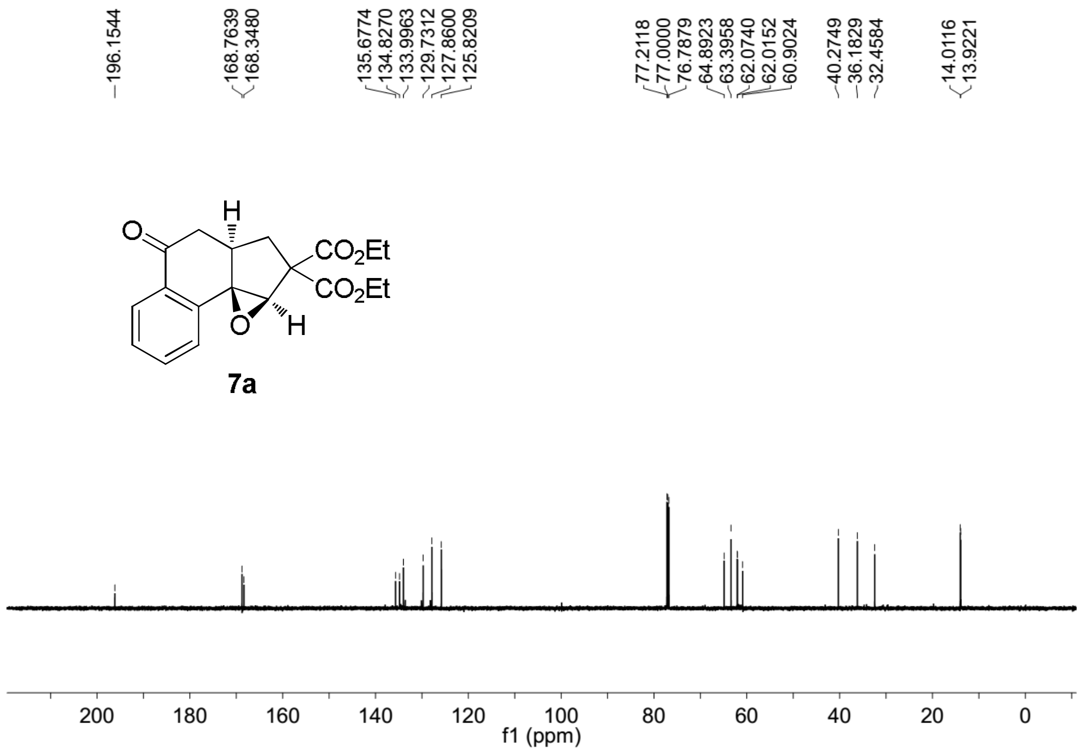
196.1544
168.7639
168.3480
135.6774
134.8270
133.9963
129.7312
127.8600
125.8209
77.2118
77.0000
76.7879
64.8923
63.3958
62.0740
62.0152
60.9024
40.2749
36.1829
32.4584
14.0116
13.9221
H
O
CO_2Et
CO_2Et
O
H
7a
200 180 160 140 120 100 80 60 40 20 0
f1 (ppm)

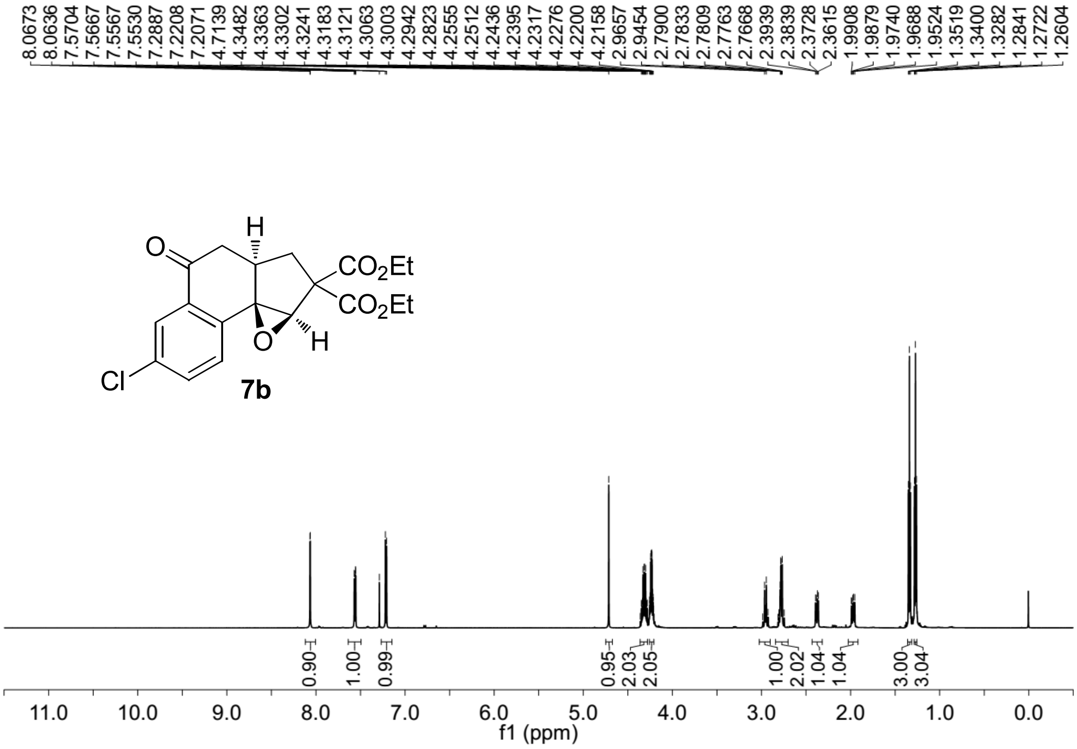
8.0673
8.0636
7.5704
7.5667
7.5567
7.5530
7.2887
7.2208
7.2071
4.7139
4.3482
4.3363
4.3302
4.3241
4.3183
4.3121
4.3063
4.3003
4.2942
4.2823
4.2555
4.2512
4.2436
4.2395
4.2317
4.2276
4.2200
4.2158
2.9657
2.9454
2.7900
2.7833
2.7809
2.7763
2.7668
2.3939
2.3839
2.3728
2.3615
1.9908
1.9879
1.9740
1.9688
1.9524
1.3519
1.3400
1.3282
1.2841
1.2722
1.2604
O
H
CO_2Et
CO_2Et
O
H
Cl
7b
0.90
1.00
0.99
0.95
2.03
2.05
1.00
2.02
1.04
1.04
3.00
3.04
11.0
10.0
9.0
8.0
7.0
6.0
5.0
4.0
3.0
2.0
1.0
0.0
f1 (ppm)

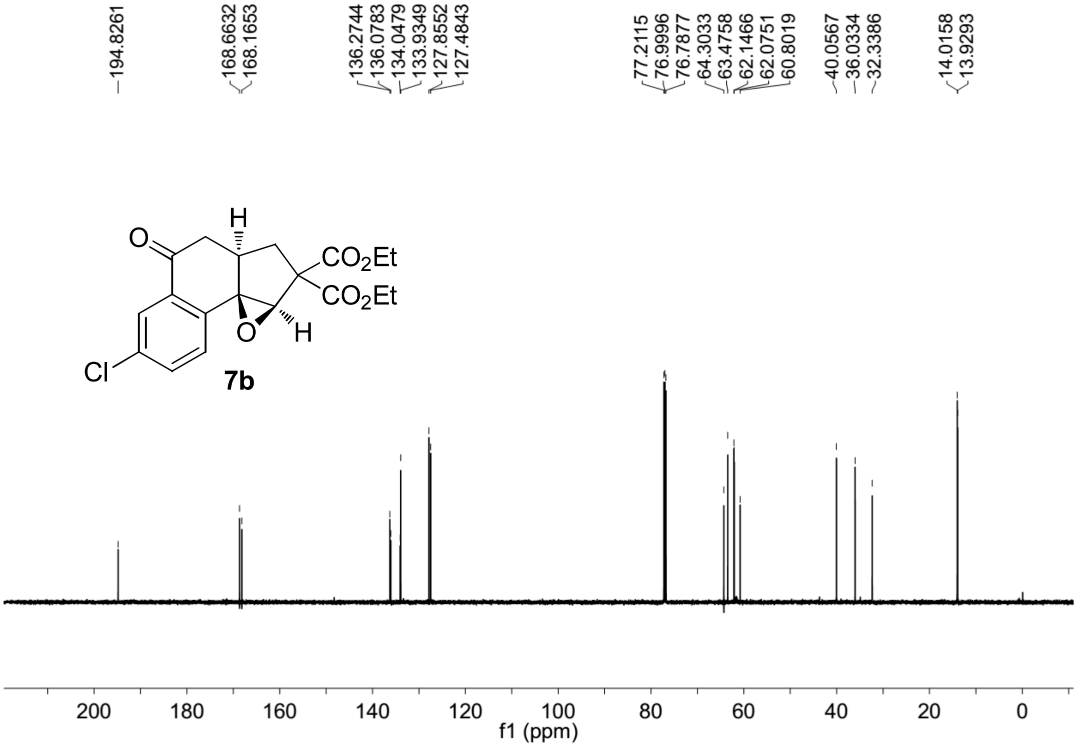
194.8261
168.6632
168.1653
136.2744
136.0783
134.0479
133.9349
127.8552
127.4843
77.2115
76.9996
76.7877
64.3033
63.4758
62.1466
62.0751
60.8019
40.0567
36.0334
32.3386
14.0158
13.9293
O
H
CO_2Et
CO_2Et
O
H
Cl
7b
200
180
160
140
120
100
80
60
40
20
0
f1 (ppm)

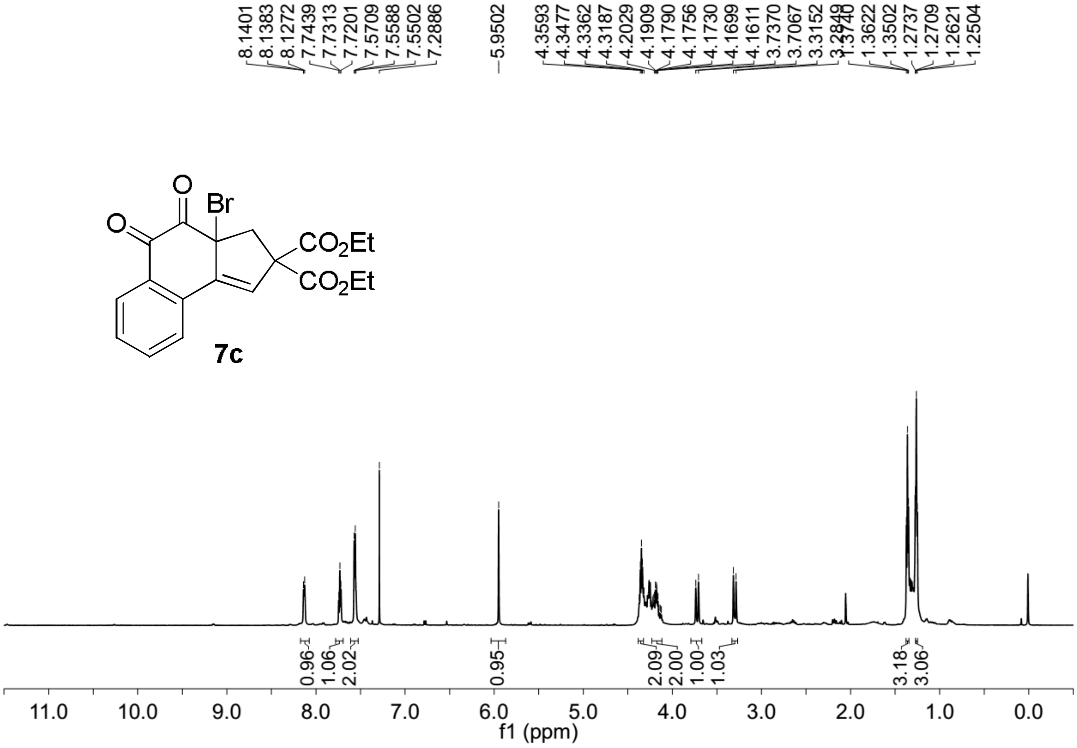

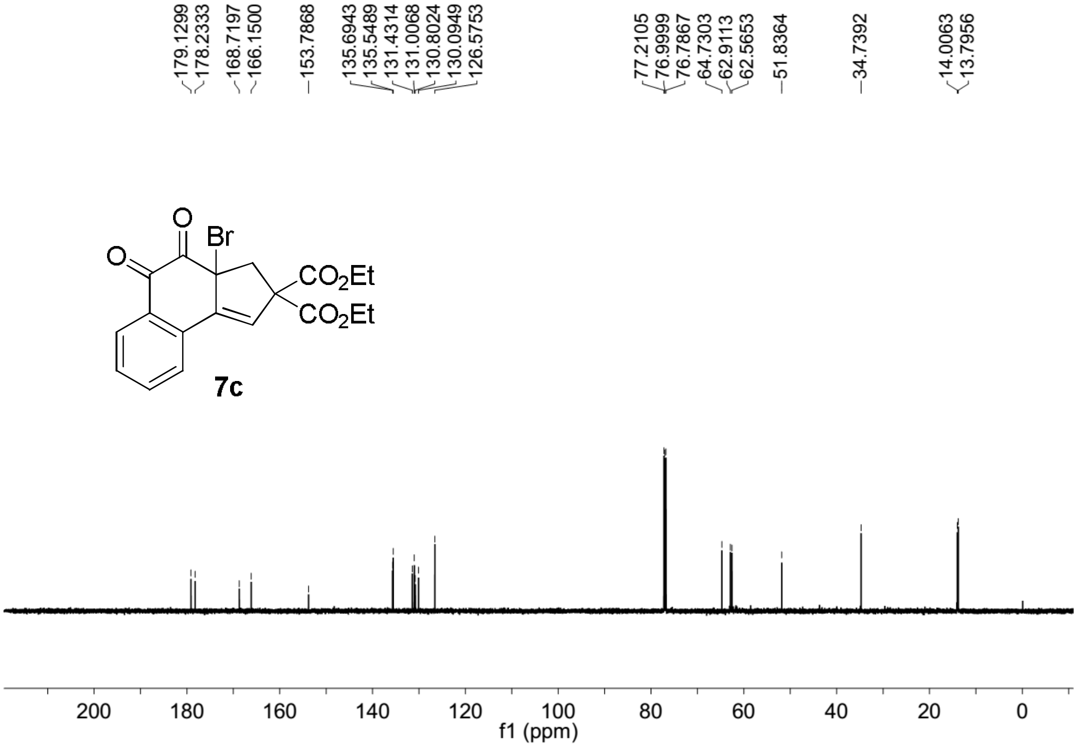

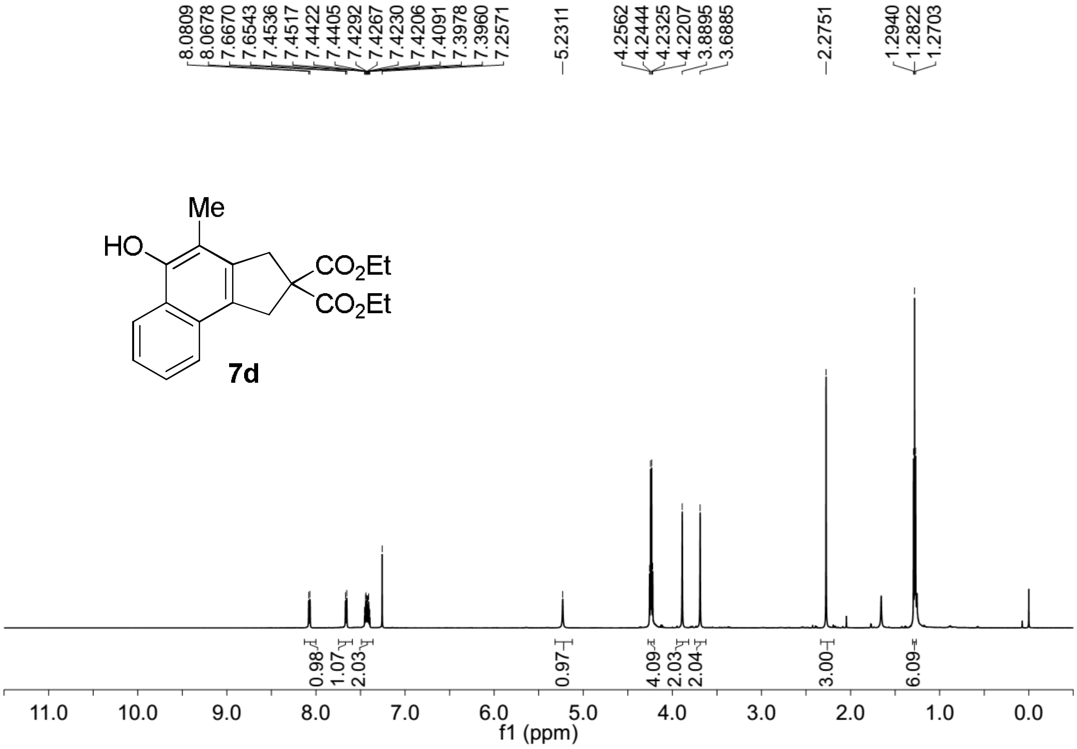
8.0809
8.0678
7.6670
7.6543
7.4536
7.4517
7.4422
7.4405
7.4292
7.4267
7.4230
7.4206
7.4091
7.3978
7.3960
7.2571
5.2311
4.2562
4.2444
4.2325
4.2207
3.8895
3.6885
2.2751
1.2940
1.2822
1.2703
Me
HO
CO_2Et
CO_2Et
7d
0.98
1.07
2.03
0.97
4.09
2.03
2.04
3.00
6.09
11.0 10.0 9.0 8.0 7.0 6.0 5.0 4.0 3.0 2.0 1.0 0.0
f1 (ppm)

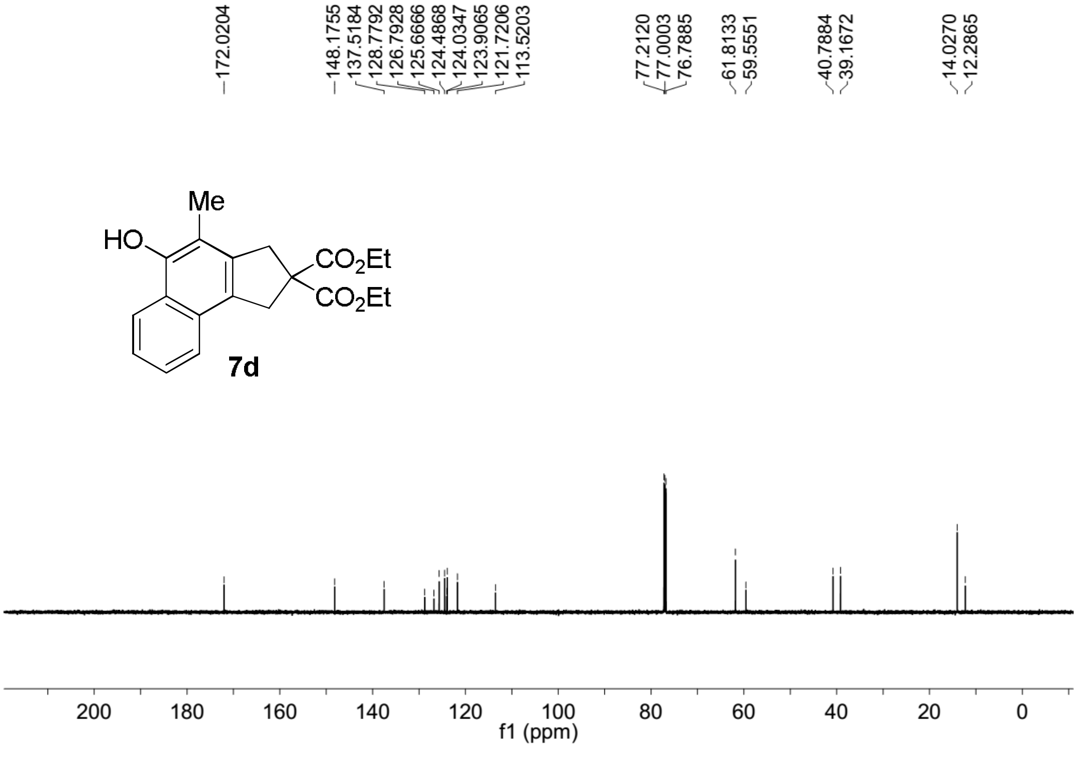
172.0204
148.1755
137.5184
128.7792
126.7928
125.6666
124.4868
124.0347
123.9065
121.7206
113.5203
77.2120
77.0003
76.7885
61.8133
59.5551
40.7884
39.1672
14.0270
12.2865
Me
HO
CO_2Et
CO_2Et
7d
200 180 160 140 120 100 80 60 40 20 0
f1 (ppm)

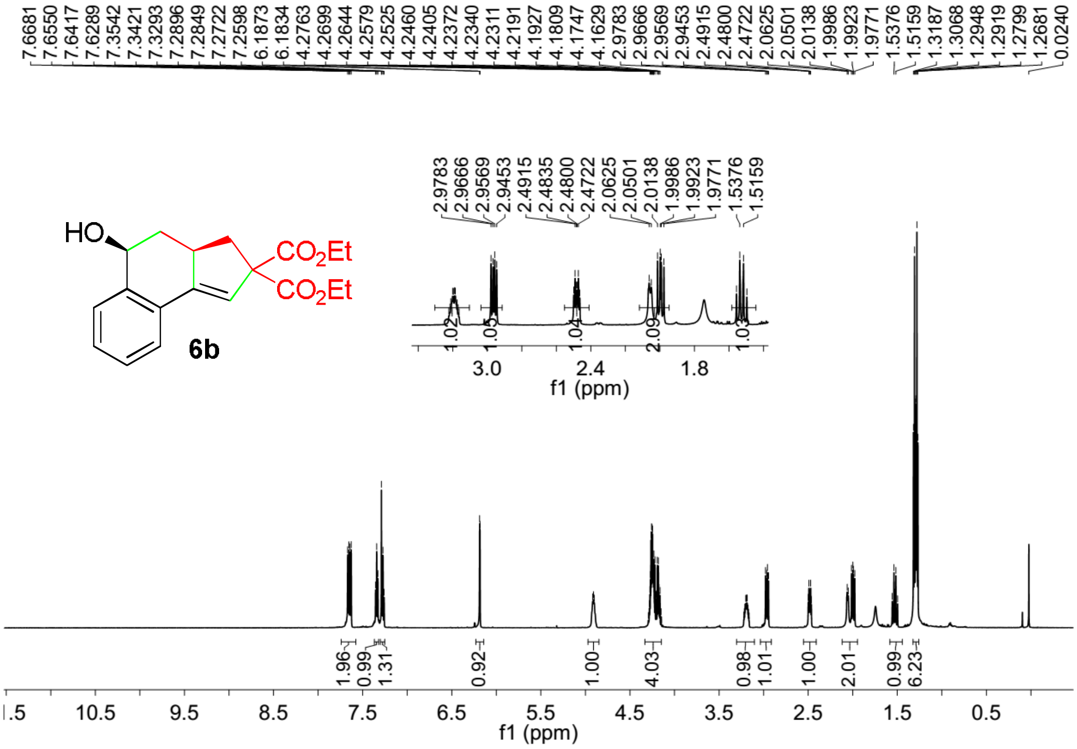
7.6681 7.6550 7.6417 7.6289 7.3542 7.3421 7.3293 7.2896 7.2849 7.2722 7.2598 6.1873 6.1834 4.2763 4.2699 4.2644 4.2579 4.2525 4.2460 4.2405 4.2372 4.2340 4.2311 4.2191 4.1927 4.1809 4.1747 4.1629 2.9783 2.9666 2.9569 2.9453 2.4915 2.4800 2.4722 2.0625 2.0501 2.0138 1.9986 1.9923 1.9771 1.5376 1.5159 1.3187 1.3068 1.2948 1.2919 1.2799 1.2681 0.0240
HO
CO2Et
CO2Et
6b
2.9783 2.9666 2.9569 2.9453 2.4915 2.4835 2.4800 2.4722 2.0625 2.0501 2.0138 1.9986 1.9923 1.9771 1.5376 1.5159
1.02 1.05 1.04 2.09 1.03
3.0 2.4 1.8
f1 (ppm)
1.96 0.99 1.31 0.92 1.00 4.03 0.98 1.01 1.00 2.01 0.99 6.23
1.5 10.5 9.5 8.5 7.5 6.5 5.5 4.5 3.5 2.5 1.5 0.5
f1 (ppm)

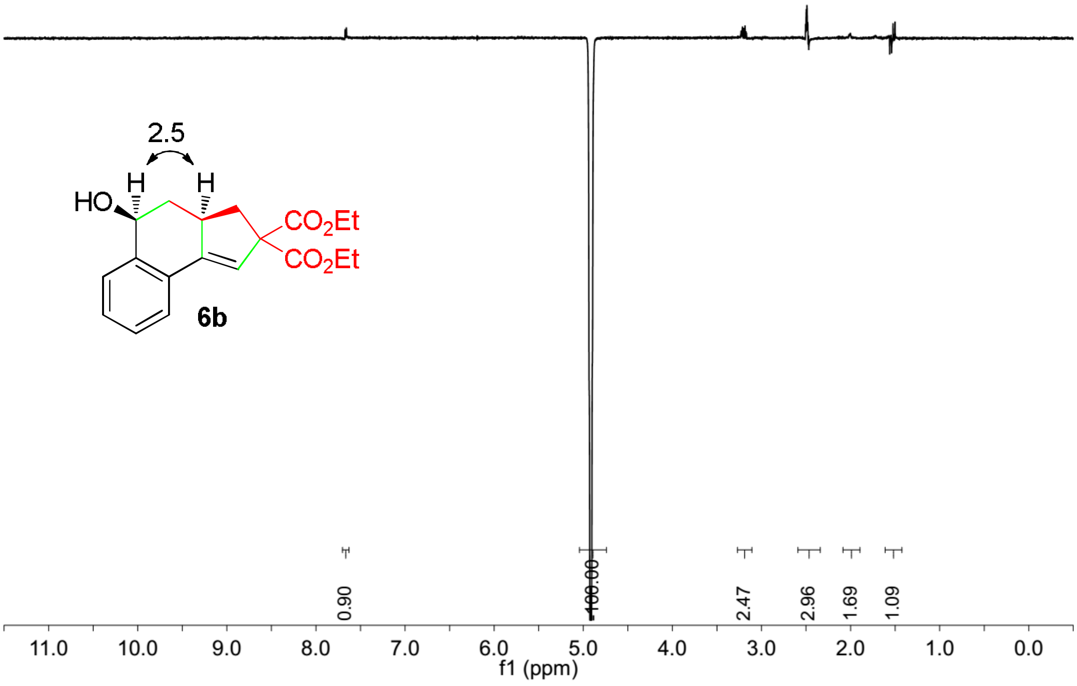
2.5
H
H
HO
CO2Et
CO2Et
6b
0.90 100.00 2.47 2.96 1.69 1.09
11.0 10.0 9.0 8.0 7.0 6.0 5.0 4.0 3.0 2.0 1.0 0.0
f1 (ppm)
